# Time-Resolved Transcriptome Analysis of *Bacillus subtilis* Responding to Valine, Glutamate, and Glutamine

**DOI:** 10.1371/journal.pone.0007073

**Published:** 2009-09-18

**Authors:** Bang-Ce Ye, Yan Zhang, Hui Yu, Wen-Bang Yu, Bao-Hong Liu, Bin-Cheng Yin, Chun-Yun Yin, Yuan-Yuan Li, Ju Chu, Si-Liang Zhang

**Affiliations:** 1 Lab of Biosystems and Microanalysis, State Key Laboratory of Bioreactor Engineering, East China University of Science & Technology, Shanghai, China; 2 Bioinformatics Center, Key Laboratory of Systems Biology, Shanghai Institutes for Biological Sciences, Chinese Academy of Sciences, Shanghai, China; 3 Shanghai Center for Bioinformation Technology, Shanghai, China; 4 College of Life Science and Technology, Tongji University, Shanghai, China; 5 Shanghai Center for Bioinformation Technology, Shanghai, China; Auburn University, United States of America

## Abstract

Microorganisms can restructure their transcriptional output to adapt to environmental conditions by sensing endogenous metabolite pools. In this paper, an Agilent customized microarray representing 4,106 genes was used to study temporal transcript profiles of *Bacillus subtilis* in response to valine, glutamate and glutamine pulses over 24 h. A total of 673, 835, and 1135 amino-acid-regulated genes were identified having significantly changed expression at one or more time points in response to valine, glutamate, and glutamine, respectively, including genes involved in cell wall, cellular import, metabolism of amino-acids and nucleotides, transcriptional regulation, flagellar motility, chemotaxis, phage proteins, sporulation, and many genes of unknown function. Different amino acid treatments were compared in terms of both the global temporal profiles and the 5-minute quick regulations, and between-experiment differential genes were identified. The highlighted genes were analyzed based on diverse sources of gene functions using a variety of computational tools, including T-profiler analysis, and hierarchical clustering. The results revealed the common and distinct modes of action of these three amino acids, and should help to elucidate the specific signaling mechanism of each amino acid as an effector.

## Introduction

Microorganisms constantly respond to the variations of external and internal conditions, and try to adapt to the environment for survival. Reconstruction of transcriptional output is found to play a crucial role during this event by means of metabolite-dependent transcriptional regulations [1]–[8]. Metabolites, as signal molecules, feed into the transcriptional regulatory systems, and bridge the gap between the metabolic and regulatory networks. The understanding of the concerted link between the genetic and metabolic components of a cell will be helpful for advancing towards an integrated network model for the understanding of cellular behavior. Such work will also help develop tools and concepts to handle and to analyze the inherent complexity of biological functions [9]. Both transcripts and metabolites change in response to external perturbation have demonstrated that the distinct alterations in transcript levels may lead to multiple changes at the level of the metabolite and vice versa, treatments with external metabolites lead to multiple changes in the abundance of transcripts, implying the bidirectional information exchange between genes and metabolites. Recently, Zaman *et al.* found that the five regulatory systems Ras/PKA, Gpr1/Gpa2, Sch9, Snf1 and Rgt2/Snf3 play different roles in responding to changes in glucose concentration and initiate cellular growth and division by using microarray gene expression profiles in conjunction with conditional mutations [10]–[12].

*B. subtilis* is a Gram-positive, spore-forming bacterium. It is one of the best-characterized bacteria, and proved to have various high levels of potential to incorporate external DNA; produce amino acids, antibacterial agents and industrially important enzymes; and secrete large amounts of proteins. The complete genome of *B. subtilis* 168, the type strain, was sequenced in 1997, and reported to encode 4,106 proteins [13]. It has been well accepted that metabolite pools of *B. subtilis* are strongly related in the regulation of the central metabolism, in agreement with the findings for *E. coli* [6]. At least 13% of 456 metabolites are involved in genetic regulation and 53% of the genes involved in metabolic pathways are directly regulated by a genetic regulator under the control of a metabolite [9]. Metabolites are also involved in the control of enzymatic activities in *B. subtilis* [9]. Thus, metabolites are key actors in the general regulation of metabolic pathways through these two levels of regulation (genetic and enzymatic) in *B. subtilis*. Recently, Abraham [14] has summarized that some global regulators are integrated into a larger regulatory scheme by which the cell coordinates the flow through key metabolic intersections in response to a small number of specific signaling metabolites in *B. subtilis*.

Herein, to better characterize the functional role of amino acids in transcriptional regulation in *B. subtilis*, we sought to systematically and quantitatively explore the effect of the amino acids on genome-wide gene expression. The comprehensive study of *B. subtilis* gene expression patterns in response to amino acid mixtures availability have been performed by proteomics and transcriptomics [15]. In these studies, Casamino Acid (CAA), a mixture of amino acids and oligopeptides and constitutes a rich nitrogen source, were used. The products of CAA-regulated genes are involved in various metabolic pathways, including nitrogen metabolism [16], [17], the development of competence [18], and chemotaxis [19]. The CodY protein has been found to play a key role in mediating the CAA-dependent regulation. However, the precise function of each amino acid as signal molecule in regulating gene expression is still unknown, and no study has attempted to comprehensively compare the genes responding to each single amino acid or the comprehensive time-course in response to these amino acids.

In the present work, we studied the effects of three amino acids (valine, glutamate, and glutamine) on gene expression in *B. subtilis* by using time-resolved transcriptome analysis. Valine is one of the branched-chain amino acids (isoleucine, valine and leucine) (BCAAs). BCAAs are the most abundant amino acids in proteins and form the hydrophobic core of the proteins. Moreover, these amino acids are precursors for the biosynthesis of iso- and anteiso-branched fatty acids, which in *B. subtilis* constitute ∼90% of membrane fatty acids during growth at 37°C. BCAAs seem to have a key regulatory role, by different mechanisms, in both gram-positive and gram-negative bacteria. The intracellular BCAA concentrations appear to serve as a gauge of the nutrient conditions, that is, a pacemaker of the synthesis of proteins and membranes. Thus BCAAs seem to be widely used by bacteria to monitor their physiological state and the nutrient conditions [20], [21]. Regulation of nitrogen metabolism in bacteria is closely connected with the intracellular levels of glutamine and glutamate, the main nitrogen donors in the cell. Glutamine is formed from glutamate and ammonium by glutamine synthetase (GS), which is a major way for the cell to assimilate ammonium. Glutamate is a central metabolite in all organisms since it provides the link between carbon and nitrogen metabolism. In *B. subtilis*, glutamate is exclusively synthesized from α-ketoglutarate, an intermediate of the tricarboxylic acid (TCA) cycle, by the glutamate synthase (GOGAT), which converts glutamine and 2-oxoglutarate into two molecules of glutamate, and it can be degraded by the glutamate dehydrogenase (RocG). Jorg Stulke has demonstrated that glutamate metabolism is subject to a variety of checks and balances that may ensure an optimal glutamate supply under all conditions [22]. It has been reviewed that the global regulators sensing some diverse intracellular metabolites (such as 2-oxoglutarate, glutamate, glutamine, and valine) provide a top layer of general nutritional regulation that determines the rate of expression of the central metabolic genes, the products of which determine the flux through the crucial metabolic intersections in *B. subtilis* [14]. Thus it is very important to uncover the functional divergence and modes of action of valine, glutamate, and glutamine on gene expression. The time-resolved transcriptional profiling in this study thereby provides an insight into the response of *B. subtilis* to different amino acids. The comprehensive comparison identified Val-, Glu and Gln-specific genes, which should help to elucidate the specific actions of each amino acid.

## Results and Discussion

### Global transcriptional in response to valine, glutamate, and glutamine pulses

We studied the changes in gene expression in the aerobic steady-state chemostat culture perturbed by the addition of valine, glutamate, or glutamine. Samples were taken at 5 min, 30 min, 2 h, 8 h, or 24 h after supplement with the amino acids and compared to a control sample before addition. While a single sample was prepared for the majority time-points, two independently cultured replicates were performed for the 5-min valine pulse experiment. These two independent pulses were highly reproducible and the median coefficient of variation (CV, S.D. divided by the mean) for the signals of red and green channels was below 6.1%. Pair-wise plots of log intensities in either channel revealed a high Pearson correlation coefficient (above 0.9, p<1e-15) (see Text S1). The M (log2 ratio) values also demonstrated significant correlation of 0.82 (for above threefold ratios), and low variation (CV) of 15.8%. All data were deposited in GEO database (Accession number: GSE17243).

**Figure 1:**
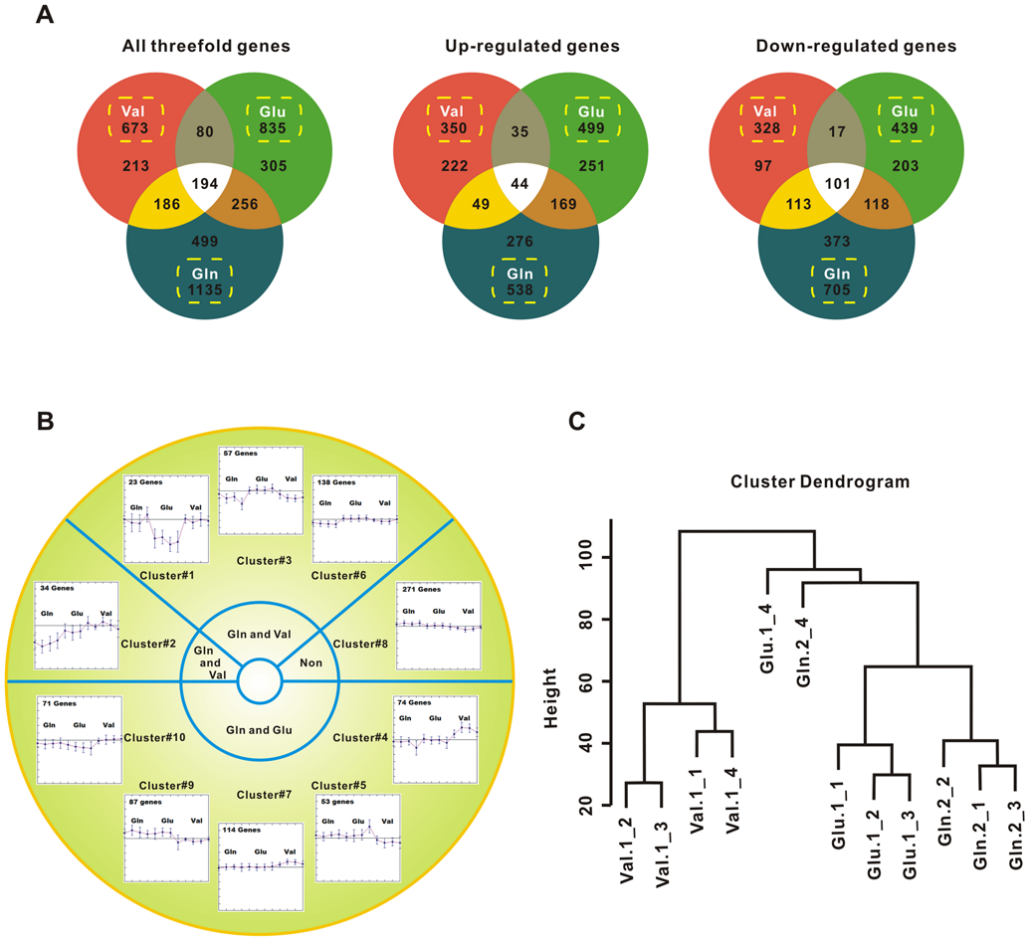
Amino Acid-responsive Genes. (A) Venn diagrams showing the overlap of genes that significantly changed after three amino acid pulses. The numbers represent a list of non-redundant genes with threefold induction or repression in response to valine (Val), glutamate (Glu), or glutamine (Gln). Numbers within boxes denote the total number of genes regulated by each amino acid. Some genes may be up-regulated at one time-point and down-regulated at another time-point. (B) Ten clusters and major functions of 922 between-experiment differentially expressed genes:Cluster #1, fatty acid β-oxidation (YsiA-regulon); Cluster #2, fatty acid biosynthesis (YlpC-regulon, des); Nitrogen metabolism (nasABC/nrgAB operon, glnA); sporulation (kipAI); transcriptional factor (GlnR, TnrA, KipR, PucR); LexA-regulated genes; Cluster #3, glutamine ABC transporter (glnQHMP); arg operon; sporulation and germination, Phage-related genes (PBSX prophage); Cluster #4, fatty acid biosynthesis (YlpC-regulon, bkd operon); bir operon; Cluster #5, PhoP-regulated genes; Mobility and chemotaxis, arg operon genes; capsular polysaccharide biosynthesis (yve operon); Cluster #6, amino acid ABC transporter genes; Phage-related genes (PBSX prophage); Specific pathways; sporulation and germination; Cluster #7, Cell wall; Fur-regulon genes; Cluster #8, Transport/binding proteins and lipoproteins; Membrane bioenergetics; Specific pathways; Metabolism of coenzymes and prosthetic groups; Phage-related functions; Cluster #9, Biosynthesis of teichuronic acid (tua operon); glutamine and BCAA ABC transporter; Cluster #10, oligopeptide ABC transporter (opp operon); surfactin synthetase srf operon. (C) Relationships among the Val, Glu, and Gln treatments:The dendrogram was calculated by hierarchical clustering using data on the expression of all Val-, Glu-, or Gln-regulated genes. The dendrogram represents the similarity of the gene expression profiles with the Val, Glu, and Gln treatments at each time point.

Gene expression upon amino acid pulses was found to be highly dynamic and involve a large number of genes. When a threefold change (pValueLogRatio<0.05) relative to the control was used as a cut-off value, 673, 835, and 1135 genes (approximately 16.4%, 20.3%, and 27.7% of all *B. subtilis* genes) were identified as significantly expressed at one or more time points in response to pulses of Val, Glu, and Gln, respectively. The identity of the up-regulated and down-regulated genes was compared among the Val-, Glu-, and Gln- pulses using Venn diagrams (Fig. 1A). The complete list of genes and expression ratios at the different time points are presented as supplementary material (Dataset S1). Given the function annotation from SubtiList Database, approximately 55.9%, 38.1%, and 40.7% of Val-regulated, Glu-regulated, and Gln-regulated genes can be assigned with known functions, respectively.

A total of 194 co-responsive genes (significantly induced/repressed at one and more time point by treatment with all three amino acids, Fig. 1A) from the three amino acid pulses were found. These mutual genes mainly involved in tryptophan biosynthesis, stress genes (*SigB* operon, *gspA, gsiB, mrgA, katA/E, cspC, csbB, nhaX*, *ctsR*), pyruvate metabolism (*alsSD* and *ldh-lctP*), purine biosynthesis, ABC transporters (*opuCABCD, pst, dpp operons* and *nrgA, csbC*), nitrogen metabolism (*ureABC, gltD*), rapA-phrA, TF (*paiA/B*), sulfate metabolism and methionine biosynthesis (*ywfG, metE, mtnA/K, sped, yxeKLMNOPQ*). There are 80 genes regulated by both Val and Glu, assigned to ciliary/flagellar motility/chemotaxis (*fla-che, mcp, motAB* and *yvyC-fliDST*), *tenAI-goxB-thiSGF-yjbV*, and *spx* (a global transcriptional regulator of the oxidative stress response); there are 186 genes regulated by both Val and Gln, assigned to threonine operon (*hom-thrC-thrB*), spore-related gene (*sps, cot, cgeABC, gerPABCDEF*), isoleucine, valine, and leucine synthesis (*ilvBHC-leuABCD*), fatty acid biosynthesis (*fapR-plsX-fabDG, fabHA, fabI*), methionine metabolism (*mtnWXB*), and *pdhA/B*; there are 256 genes regulated by both Glu and Gln, including arginine biosynthesis genes(*arg*), *kipIAR, glnMP, pucJKL, yxbB-yxbA-yxnB-asnH-yxaM* (*asnH* gene encoding asparagine synthetase), *lcfA* operon, *hpr, gltC, gltA, fur, sigM, sigW*, and many function-unassigned genes.

To reveal patterns of temporal gene expression and to identify potential coregulated genes, data were analyzed using a number of tools implemented in the Microarray Expression Viewer software (MEV-TIGR; http://www.tm4.org/mev.html), such as hierarchical clustering and *K*-means cluster analysis. Analysis of three sets of 673, 835, and 1135 genes by *K*-means clustering identified eight Val-responsive gene clusters, ten Glu-responsive gene clusters, and ten Gln-responsive gene clusters using the average linkage (weighted-pair group method using average linkages) clustering method and a Euclidian distance metric (Dataset S1). Clusters 1, 3, 5, and 6 of Val grouped genes the expression of which was decreased after Val addition, whereas clusters 2, 4, 7 and 8 showed the opposite trend. The Cluster 1 consisted of 42 significantly down-regulated genes, assigned to *trpEDCFBA, rsbRSTUVW-sigB-rsbX, ldh-lctP* operons and other sigma-B-controlled genes. The Cluster 3 consisted of 99 significantly repressed genes, assigned to *ilvBHC-leuABCD, tenAI-goxB-thiSGF-yjbV, ureABC*, ABC transporters (*opuCABCD* and *dppABCDE*) operons, some spore coat genes, and the transcriptional regulators (*parA, paiB, tenA, and tenI*). The Cluster 5 and 6 consisted of repressed 187 genes, assigned to ciliary/flagellar motility/chemotaxis (*fla-che, mcp, motAB and yvyC-fliDST*), *sps*, and some SigK-regulated genes. Groups 2 and 8 consisted of approximately 246 genes, were found to be up-regulated, assigned to *ptb-bcd-buk-lpdV-bkdAABB*, *pur*, *xpt-pbuX*, *bioWAFDBI* operons, and some SigW-regulated genes. The Cluster 4 and 7 mainly assigned to phosphate ABC transporter (*pst*) and YlpC-regulated genes.

Clusters 1 and 7 of Glu consisted of 186 genes the expression of which was greatly decreased after Glu addition, whereas overexpressed at late stages (24 hr following Glu pulse). These genes assigned to *tenAI-goxB-thiSGF-yjbV, rsbRSTUVW-sigB-rsbX, ldh-lctP*, ABC transporters (*opuCABCD, glnQHMF*, and *dppABCDE*) operons, and other sigma-B-controlled genes. 116 genes, such as *yhbIJ-yhcABCDEFGHI*, *lcfA-ysiAB-etfBA, yusMLKJ, ureABC, pucRJKLM* operons and some transcript regulators (*hpr, paiA, paiB, gltC, spxA*), were found to be repressed after glutamate pulse. Cluster 2 and 6 represented the transcripts with that were up-regulated at time point of 24 hr, including *fla-che, motAB, yvyC-fliDST, trpEDCFBA*, *kipIAR*, *eps* operons, Na^+^/H^+^ antiporter(nhaC), and many genes of unknown function, whereas clusters 3 and 8 included approximately 143 genes, found to be down-regulated at late stages. Most of these genes are unassigned genes excepting *srf, rap, sac, sped, comS, degQ, degU, lic, fur* genes. Approximately 100 (Cluster 4) genes were induced in the time frame between 5 min and 2 h after glutamate addition, including the genes involved in purines/pyrimidines metabolism and BCAA synthesis. Some operons, such as *argCJBD, pst, yxeKLMNOPQ, tuaABCDEFGH, phoB-ydhF, pheST, and gcv*, were found to be induced after approximately 30 min.

Clusters 1 of Gln consisted of 45 genes were remarkably downregulated after glutamine addition, assigned to TnrA- and GlnR-regulated nitrogen metabolism (*glnRA, nrgAB, nasABC, kipIAR, tnrA, pucR*) and ABC transporters (*opuBABCD* and *glnQHMF*). 278 genes (Cluster 2 and 5) were slightly repressed early, and prominently repressed at late stages (24 hr following Gln pulse). These genes are involved in gerE-, spoIIID-, spoVT- and comK-regulated sporulation/germination functional categories and the phage-related functions (*xtm* and *xkd* operons). Cluster 4 included 37 genes repressed after 2 h following glutamate addition, mainly related to purines/pyrimidines metabolism and some SigE-controlled genes. Cluster 6 presented the transcripts of which were up-regulated at time point of 24 hr, including *pst, alsSD, menBEC* operons, and many genes of unknown function. Approximately 245 genes, repressed after 30 min, divided into two clusters: cluster 7 including amino acid metabolism (*argCJBD, trpEDCFBA, rocBC, leuCD), ureABC, puc, dppABCDE, senS* and some SigW-controlled genes; cluster 8 comprising genes overexpressed at time point of 24 hr, assigned to *rsbRSTUVW-sigB-rsbX, ldh-lctP, opuBABCD* operons, ctsR transcript regulator, and other sigma-B-controlled genes. Approximately 241 (Cluster 10) genes were induced in the time from 5 min to 2 h after glutamine addition, including the genes involved in purines metabolism, S-box-regulated transcriptional units (*cysHP*-*sat*-*cysC*-*ylnDEF*, *yjcIJ*, *yoaDCB*, *ykrWXYZ*, *ykrTS*, and *yitIJ*), *motPS, narGHJI, gcv, pdhAB* operons.

In addition to examine the identity of genes regulated by different amino-acid pulses, we also compared the temporal expression log ratios between each pair of amino-acid pulses, yielding 922 between-experiment differentially expressed genes (briefed as DEGs below) that had different temporal expression patterns between some two amino-acid pulses. The complete list of genes and hierarchical clustering results are presented in Table S1. These DEGs were divided into ten clusters. The results clearly revealed the common and distinct modes of action of three amino acids (Fig. 1B).

The relationship between different time-points of the Val, Glu, and Gln treatments were explored using hierarchical clustering of the expression profiles of the 922 between-experiment DEGs. The dendrogram indicated that the Val, Glu, and Gln treatments clustered independently (Fig. 1C). In each cluster of the Val, Glu, and Gln treatments, the continuous experiments were related vicinally. In addition, the dendrogram suggests that Glu and Gln treatments induce more similar gene expression changes than Val treatment. However, Gln was considered more impactive in *B. subtilis* transcriptome, as it regulated many more genes than Glu (Fig. 1A).

### Identification of rapidly responding genes

**Figure 2:**
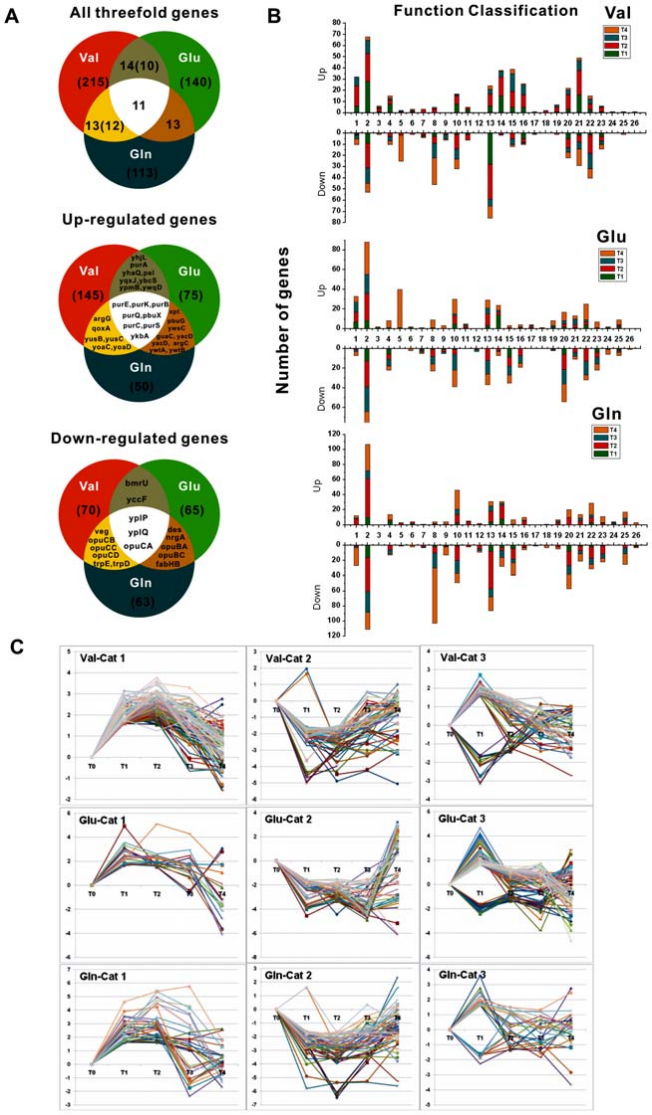
Rapidly AA-responsive Genes. (A) Venn diagrams about the number of rapidly Val-, Glu-, and Gln-responding genes: all three-fold responsive genes at 5 min; the three-fold induced genes at 5 min; the three-fold repressed genes at 5 min; There are four genes (yhgD, pel, cydA, and cydB) showing converse expression between Val and Glu; one gene ilvB, showing converse expression between Val and Gln. (B) Classification of all amino acid-responsive genes: All valine-(Val), glutamate-(Glu), and glutamine-(Gln) responsive genes with known-function were classified according Subtilist Database (http://genolist.pasteur.fr/SubtiList/). 1, Cell wall; 2, Transport/binding proteins and lipoproteins; 3, Sensors (signal transduction); 4, Membrane bioenergetics; 5, Mobility and chemotaxis; 6, Protein secretion; 7, Cell division; 8, Sporulation; 9, Germination; 10, Specific pathways; 11, Main glycolytic pathways; 12, TCA cycle; 13, Metabolism of amino acids and related molecules; 14, Metabolism of nucleotides and nucleic acids; 15 Metabolism of lipids; 16, Metabolism of coenzymes and prosthetic groups; 17, Metabolism of phosphate; 18, Metabolism of sulfur; 19, DNA recombination; 20, RNA synthesis; 21, Protein synthesis; 22, Adaptation to atypical conditions; 23, Detoxification; 24, Antibiotic production; 25, Phage-related functions; 26, Transposon and IS. (C) Three expression categories of rapidly responsive genes: In category 2 of valine (Val-Cat 2), cydBC genes, encoding cytochrome bd ubiquinol oxidase and ABC membrane transporter, were upregulated at 5 min, whereas downregulated after 30 min.

In order to understand the mechanism by which *B. subtilis* reprograms the transcription and the cellular processes after a sudden addition of amino acid, rapidly responding genes within the first 5 min were studied. Approximately 215, 140, and 113 genes were found to be threefold changed during the first 5 min after treatment with valine, glutamate, and glutamine, respectively (Fig. 2A). The complete list of genes and change folds are presented in Table S2.

Genes whose transcripts respond rapidly were good candidates for upstream transcriptional signaling components. These early responding genes were divided into three categories: the genes with the significant induction at both 5 min and 30 min (category 1); the genes with the significant repression at both 5 min and 30 min (category 2); the genes with transient change at 5 min and reversion at 30 min (category 3). About half of 215 rapidly valine-responsive genes (105) were in category 1, involved in cell wall, transport of nutritions (glucose, sugar, xanthine, and amino acids), ATP synthesis, pyruvate dehydrogenase *pdh*, synthesis of cysteine, biotin, purine, and ribosomal proteins, utilization and degradation of BCAAs. The most responsive genes were *ybcC, yrrT, purC* (7–8 folds).

There were 60 rapidly valine-responsive genes in category 2, including gene involved in dipeptide or oligopeptide ABC transporters, synthesis of amino acids and thiamine, and sporulation. The most responsive genes were *trp* operon (30-fold) and *mrgA* (metalloregulation DNA-binding stress protein). 50 genes in category 3 included gene encoding ABC transporters, oxidase genes related to membrane bioenergetics (Fig. 2C).

Above half of rapidly glutamate-responsive genes (75 genes) were in category 3. The most of these genes were involved in biosynthesis of nucleotides (*pyr* and *pur* operon), lactate metabolism (*lctP-ldh, cydAB*), and oligopeptide ABC transporters (Fig. 2C). The most responsive genes were *pur* operon and *xpt* gene (induction of 25–30 folds at 5 min).

Only 18 genes were in category 1, including main glycolytic pathways *gapB*, xanthine (*pbuX, xpt*), metabolism of phosphate *phoD*, and capsular polyglutamate biosynthesis genes. *gapB* gene was the most responsive (induction of 35 folds). Category 2 contained 47 genes, involved in transport (*opu and nrgA*), desaturation and β-oxidation of fatty acid, transcriptional regulation (*yhgD, yplP, ysiA, ydgG, nhaX*).

Interestingly, half of rapidly glutamine-responsive genes (57 genes) were in category 2, including genes, involved in the transports of ammonium, nitrate, and acids, *opu* operon, assimilation of other nitrogen resourses (*nrgB, nasBC*), synthesis of trp and gluamine, desaturation and biosynthesis of fatty acid, degradation of xanthine, transcriptional regulation (*glnR, senS, gltC, kipIAR, yplP, tnrA, pucR*). The most responsive genes were *des* and *nrgA* (repression of 55-40 folds). 31 genes were in category 1, involved in the transport and synthesis of nucleotides, capsular polyglutamate biosynthesis. Among them, ybgH, encoding sodium/proton-dependent alanine transporter, was most significantly induced (55-fold). Category 3 contained 25 genes, such as *narGHJI* (nitrate reductase), *ytrAB*, and *ssuB* (Fig. 2C).

Among the all significantly rapidly expressed genes, 145, 75, and 50 genes were identified as induced by Val, Glu, and Gln, respectively. These genes are involved in transport functions, metabolism, cell wall, energy, RNA synthesis, and protein synthesis (Table 1). The largest set of upregulated genes was those induced by valine (145 genes). Eight genes was upregulated by treatment with all three amino acids, including six purine biosynthesis genes, one xanthine permease, and one amino acid permease gene. 70, 65 and 63 genes were identified as downregulated by Val, Glu, and Gln, respectively. Only three genes were mutually repressed by three amino acids. The most of the rapidly responding genes did not share similar gene expression (degree of overlap, 10–20%), indicating the Val, Glu, and Gln displayed unique transcriptional regulation functions as effectors independently. Valine was more active in regulating the genes rapidly in this study, as it induced a total of 145 genes and repressed 70 genes at 5 min.

**Table 1:** The functional classification of rapidly responding genes.

Functional classification	Up (Val)	Down (Val)	Up (Glu)	Down (Glu)	Up (Gln)	Down (Gln)
Cell wall	6	1	6			
Transport and lipoproteins	28	9	8	13	9	16
Sensors (signal transduction)	1		1			
Membrane bioenergetics	8		1	6	5	
Mobility and chemotaxis			1			
Protein secretion			1		2	
Cell division; Sporulation; Germination		3	1	3	1	2
Transformation/competence			1			
Metabolism of carbohydrates/related molecules	11	2	6	1	3	1
Metabolism of amino acids/related molecules	6	28	3		4	8
Metabolism of nucleotides/nucleic acids	15		14		9	3
Metabolism of lipids	5			9		4
Metabolism of coenzymes/prosthetic groups	5	5	1			
Metabolism of phosphate			1			
DNA recombination	1		2			
RNA synthesis	3	5	2	6		8
protein synthesis	14					
protein modification and folding	2	2	1			1
Adaptation to atypical conditions	3	2	4		3	2
Detoxification	1	3		5		1
Antibiotic and phage			3		2	
Transposon and IS						1
Miscellaneous and unassigned function	35	10	18	22	12	16

One of the earliest significantly overrepresented gene ontology groups encoded proteins involved in transport of various molecules. It was found that the large uptakes of ions, amino acids, sugars, nucleotides, phosphate were activated. The results suggested that transporters maybe play a key role after amino acid addition. Dipeptide transport system *dpp* operon, expressed early during sporulation or nutrient deficiency conditions, and osmotically regulated *opu* operon were repressed. An other prominent group of genes that were overrepresented at 5 min after amino acid addition encoded proteins involved in metabolism, including 13 (val), 7 (glu), 4 (gln) genes of metabolism of carbohydrates; 33 (val), 3 (glu), 11 (gln) genes of metabolism of amino acids; 15 (val), 14 (glu), 12 (gln) genes of metabolism of nucleotides; 5 (val), 9 (glu), 4 (gln) genes of metabolism of lipids; 10 (val), 1 (glu), 0 (gln) genes of metabolism of coenzymes/prosthetic groups. From the analysis of overrepresented gene ontology terms, it was suggested three amino acids result in different reprograming of the cell processes during the early stages (at 5 min). Valine rapidly activated the transport system of various molecules, electron transport chain and ATP synthesis, utilization of carbohydrates, salvage and biosynthesis of purine, utilization and degradation of BCAA, biotin and cysteine synthesis, and translational process, and inactivated the biosynthesis of amino acids (Phe, Tyr, Trp, Ile, Val, Leu, and Glu), NAD, and thiamine. Glutamate activated the uptake and biosynthesis of nucleotides, and the capsular polyglutamate biosynthesis, and inactivated theββ-oxidation of fatty acid and the electron transport. Glutamine significantly induced the uptake and biosynthesis of nucleotides, and the capsular polyglutamate biosynthesis, and repressed the assimilation and utilization of other nitrogen source (ammonium, nitrate), degradation of pyrimidine, uptake and utilization of acetoin, biosynthesis of Gln, Trp and fatty acid.

To overview the functional overlap and divergence of Val-, Glu-, and Gln-inducible genes, the genes were classified into 26 categories based on their established or putative functions (Subtilist Database). The numbers of Val-, Glu-, and Gln-inducible genes are shown in Fig. 2B. The largest group of regulated genes was the genes related to transport proteins. This histograms represented the general trend of valine-, glutamate-, and glutamine-regulated genes well, i.e. valine function as an effector was quicker than glutamate and glutamine for reconstructing the transcriptional output. Compared with valine, a few genes responded to glutamate and glutamine at 5 min, and the number of glutamate/glutamine-regulated genes increased continuously at 30 min, especially genes related to transport system and amino acid metabolism. A clear mechanism for the difference in induction response is unknown. The difference in induction response suggested that valine maybe directly regulate the transport system and amino acid metabolism, whereas glutamate/glutamine displayed indirect effects on them through mediation of other regulators. These results may also reflect the different modes of action in the valine, glutamate, and glutamine signal transduction.

#### Transport

A large number of genes involved in transport of various molecules were found to be overrepresented within the first 5 min. This group included phosphotransferase system (PTS), ABC transporters, and permeases. These transporters are devoted to the transport of ions, amino acids, sugars, nucleotides, phosphate, and other organic compounds. While the most transport genes were rapidly activated after addition of valine. A few genes (8 and 10 genes) were overexpressed at 5 min, whereas many genes (28 and 51 genes) were activated at 30 min after addition of glutamate and glutamine (Fig. 2B). The immediate initiation of transport functions is likely to be necessary to rapidly supply the cell with the essential elements (amino acids, carbohydrates, and nucleosides) for rapid growth. The transporters induced by glutamate mainly were the nucleoside transporter genes (*pyrP, pbuX, pbuG*), putative amino acid permease gene *ykbA*, and cation-efflux system gene *czcD*. Fujita *et al* revealed that glutamine in the medium is able to trigger expression of the *ybgH* (*glnT*) gene, which encodes the glutamine transporter, through signal transduction via a two-component system YcbA-YcbB (GlnK-GlnL)[23]. The observation was further confirmed by our data. The *ybgH* gene was greatly induced (25 folds) at 5 min after glutamine addition. However, the induction of *ycbA-ycbB* genes expression was not found. Valine repressed 9 ABC transporter genes at 5 min, including the dipeptide ABC transporter operon (*dppBCDE*), glycine betaine/carnitine/choline ABC transporter operon (*opuC*), and the putative oligopeptide ABC transporter gene *ykfD*. CodY activated by valine obviously repressed *dppBCDE* operon. The simultaneous repression of expression of the glycine betaine transport protein by glutamate (*opuBAC, opuCA*) and glutamine (*opuBABC, opuCABCD*) may suggest that osmotic defense is suppressed early in response to amino acid treatment. If the favoured nitrogen source, glutamine, is available, TnrA is inactivated, as it represses genes such as ammonium transporter genes *nrgAB* and nitrate transporter/assimilation genes *nasABC*, the products of which mediate the uptake of ammonium ion, nitrate and nitrite. Indeed, *nrgAB* (30 and 9 folds) and *nasABC* (7, 9, and 6 folds) were immediately repressed after glutamine addition.

### Metabolism

A large number of genes involved in metabolism were also found to be overexpressed during the early stages of treatment with amino acids. This group included genes for the metabolism of carbohydrates and related molecules, amino acids and related molecules, nucleotides and nucleic acids, lipids, coenzymes and prosthetic groups, and phosphate. Valine activated expression of genes *pdhAB*, encoding pyruvate dehydrogenases at 5 min, whereas significantly repressed transcription of the *gapA-pgk-tpiA-pgm-eno* operon encoding five key glycolytic enzymes that catalyse the five steps of the central carbon metabolism at 5–30 min. Meanwhile, transcription of *gapB* gene in gluconeogenic pathway was obviously induced by glutamate (34 folds) and glutamine (5 folds) at 5–30 min. Valine exhibited the rapid and strong effect on metabolism of amino acids: it induced 6 genes, and repressed 28 genes related to amino acid biosynthesis at 5 min. Phenylalanine and tyrosine biosynthesis genes *tyrA* and *aroABEFH*, tryptophan biosynthesis genes *trpABCDEF* (30 folds), isoleucine, valine and leucine biosynthesis genes *ilvABCDH* and *leuABCD*, glutamate synthase gene *gltB*, and urea cycle and metabolism of amino groups genes *ureAB* were found to be repressed. Only three genes (*trpDE, glnA*), involved in amino acid biosynthesis, were repressed at 5 min by glutamine. The aspartate aminotransferase gene *aspB*, arginine biosynthesis gene *argG*, the thiolation pathway gene *cysH* and reverse transsulfuration pathway (homocysteine-to-cysteine conversion) genes *yrhAB* of two major cysteine biosynthetic pathways were found to be overexpressed at 5 min after valine addition. Glutamate activated three AA-metabolism genes at 5 min: *argC* (arginine biosynthesis), *dapB* (lysine biosynthesis), and *bcsA* (phenylalanine metabolism). The *argCG* genes were also induced at 5 min by glutamine.

Fifteen, 14, 9 genes, involved in metabolism of nucleotides and nucleic acids, were rapidly induced by valine, glutamate, and glutamine, respectively, including *purA xpt, gua genes* and *pur, pyr* operons encodes the enzymes which catalyze de novo biosynthesis of purine and pyrimidine. The glutamine obviously repressed the expression of *pucABC* genes immediately, encoding xanthine dehydrogenases as first step of hypoxanthine and xanthine degradation pathway.

Valine led within 5 min the clear induction of *bkd* operon, involved in the control of isoleucine and valine utilization and degradation for biosynthesis of branched chain fatty acid, as well as *accD* gene for long-chain fatty acid biosynthesis. Glutamate displayed the strongest effect on fatty acid metabolism. Different with valine, it significantly repressed nine genes, mainly involved to the fatty acid β-oxidation pathway, includes four operons (*lcfA-ysiA-B-etfB-A, yhfL, yusM-L-K-J*, and *ywjF-acdA-rpoE*). These genes are *YsiA*-regulon. The *ysiA*, as a negative regulator gene, itself was repressed (15 folds). The fatty acid desaturase gene *des*, involved in the formation of unsaturated fatty acids for membrane phospholipid, was repressed by glutamate (14 folds) and glutamine (55 folds). The *fabHB* and *fabI*, involved in biosynthesis of fatty acid, were repressed at 5 min by glutamine. Three amino acids exerted the different instant effects on fatty acid metabolism: valine activated BCAA degradation pathway to provide some precursor molecules for biosynthesis of branched chain fatty acid; glutamate inhibited the fatty acid β-oxidation pathway; glutamine restrained the fatty acid biosynthesis pathway.

### Bioenergetics

Valine induced eight bioenergetics-related genes, including 4 ATP synthase genes (*atpAEFH*) and 4 cytochrome oxidase genes (*qoxAC*, *cydAB*). Glutamine activated nitrate reductase *narGHJI* operon, which are generally induced by Fnr under anaerobic conditions. However, the overexpression of transcriptional regulator Fnr and the two-component system ResDE, involved in the adaptation of the bacterium to low oxygen tension and anaerobiosis, was not found. The flavoprotein genes *etfB, etfA*, and putative iron-sulphur-binding reductase gene *ywjF* of *YsiA*-regulon related to fatty acid β-oxidation pathway were significantly repressed (14–16 folds) by glutamate. Contrary to valine, glutamate inhibited the expression of cytochrome oxidase genes *cydAB*.

### Gene transcription and regulation

Some genes involved in transcription and the regulation of transcription were found to be overrepresented during the early stages of amino acid addition. Copper is an essential element for *B. subtilis* aerobic respiration because it is a cofactor for haem-copper oxidases, the terminal enzymes in the respiratory pathway. Recently the mechanisms underlying copper homeostasis of *B. subtilis* has been elucidated [24]–[25]: *ycnJ* gene, involved in copper acquisition, and *copZA* (*yvgXY*) operon encoding Cu-specific efflux system were regulated by YcnK and CsoR(YvgZ). The *ycnKJ* genes were induced at 5 min, and *yvgYZ* genes (3 and 5 folds) were repressed at 30 min after valine addition. As a result, copper ion levels will be changed in cells. Valine also induced the expression of transcriptional regulator gene birA, eliciting the synthesis of biotin, an essential cofactor for a class of important metabolic enzymes.

Five transcriptional regulator genes were repressed at 5 min after valine pulse, including general stress factor sigma *sigB*, sigma-L modulating factor *yvyD*, positive regulator of extracellular enzyme genes *tenAI*, and *yplP*. Interestingly, *yplP* was only transcriptional regulator gene which of expression was mutually threefold regulated at 5 min by three amino acids. We suggest that *yplP* may be involved in amino acid metabolism, and *yplQ* coexpressed with *yplP* formed an operon structure. Beside *yplP* and *ysiA*, two putative TF genes *yhgD* (9 folds) and *ydgG* (15 folds), *nhaX*, and *yhcB* were repressed by glutamate. NhaX (YheK) plays a role regulating NhaC (YheL), a Na+/H+ antiporter thought to aid pH homeostasis under alkaline conditions. No TF gene was induced at 5 min by glutamine. Eight transcriptional regulators were found to be repressed in this stage, several of which are believed to be involved in nitrogen metabolism, such as TnrA, GltC, and GlnR. It is not surprising that glutamine exhibited significant effects on the expression of these genes. These results demonstrated that the metabolite flux from 2-oxoglutarate to glutamate to glutamine was reduced. Other transcriptional factors at lower expression level are SenS involved in secretion of degradative enzymes, the regulator PucR of purine degradation, the regulator KipR of the *kip* operon, and also the regulator YtrA of *ytrABCDEF* operon, which has been shown to be involved in acetoin utilization. The *kipR* and *pucR* were regulated by TnrA. The *ytrB* and *ytrC* genes of *ytrABCDEF* operon were repressed by glutamine to affect acetoin utilization. The *kipA* and *kipI* genes of the *kip* operon, involved in sporulation, were also downregulated.

A prominent group of genes that were upregulated at 5 min following valine addition were involved in protein synthesis. This group of genes included the 50S ribosomal protein genes; 30S ribosomal protein genes, threonyl-tRNA synthetase genes *thrSZ*, elongation factor gene *tsf*, and ribosome recycling factor gene *frr*. This major induction of ribosome proteins is indicative of a rapid synthesis and recruitment of the translational machinery. Glutamate and glutamine did not display rapid induction of translational machinery. Recently, Brauer *et al.* reported that a subset of the nutrient-regulated genes changes expression in response to nutritional status strictly dependent on growth rate but independent of the particular nutrient that limits the growth of the cells [26]. The expression levels of some of the genes, such as ribosome biogenesis genes, are directly proportional to the instantaneous growth rate. The levels of expression of members of this collection of genes provide a ‘growth rate signature’ that is highly predictive of the instantaneous growth rate of the cells from which the sample was taken. Based on Brauer's reseach, the induction of genes involved in ribosome biogenesis by valine is indicative of a rapid growth of *B. sublitis*. From data, we found that only valine revealed a strong inducible effect on the instantaneous growth rate of *B. sublitis*.

### Sig-regulons responding to Val, Glu, and Gln

Sigma factors play a central role to restructure the transcriptome responses to environmental signals. The microarray data were analyzed using T-profiler to identify some sigma factors in response to amino acids. T-profiler is a computational tool that uses the *t*-test to score changes in the average activity of predefined groups of genes based on Gene Ontology categorization, upstream matches to a consensus transcription factor binding motif, or KEGG pathway. It can be employed to determine significantly regulated gene groups. T-profiler, developed originally for the analysis of *S. cerevisiae* transcriptome data [27], was adapted for the analysis of genome-wide expression data for *B. subtilis* by Alex Ter Beek *et al.* [28]. All microarray data were uploaded online for T-profiler analysis (http://www.science.uva.nl/~boorsma/t-profiler-bacillusnew/). T-profiler transforms transcriptional data of single genes into the behavior of gene groups, reflecting biological processes in cells (TF model, KEGG model, and Subtilist model). All gene groups with significant *T* values (E-value<0.05, TF model, KEGG model, and Subtilist model) in any time point are presented in Tables S3–5.

**Figure 3:**
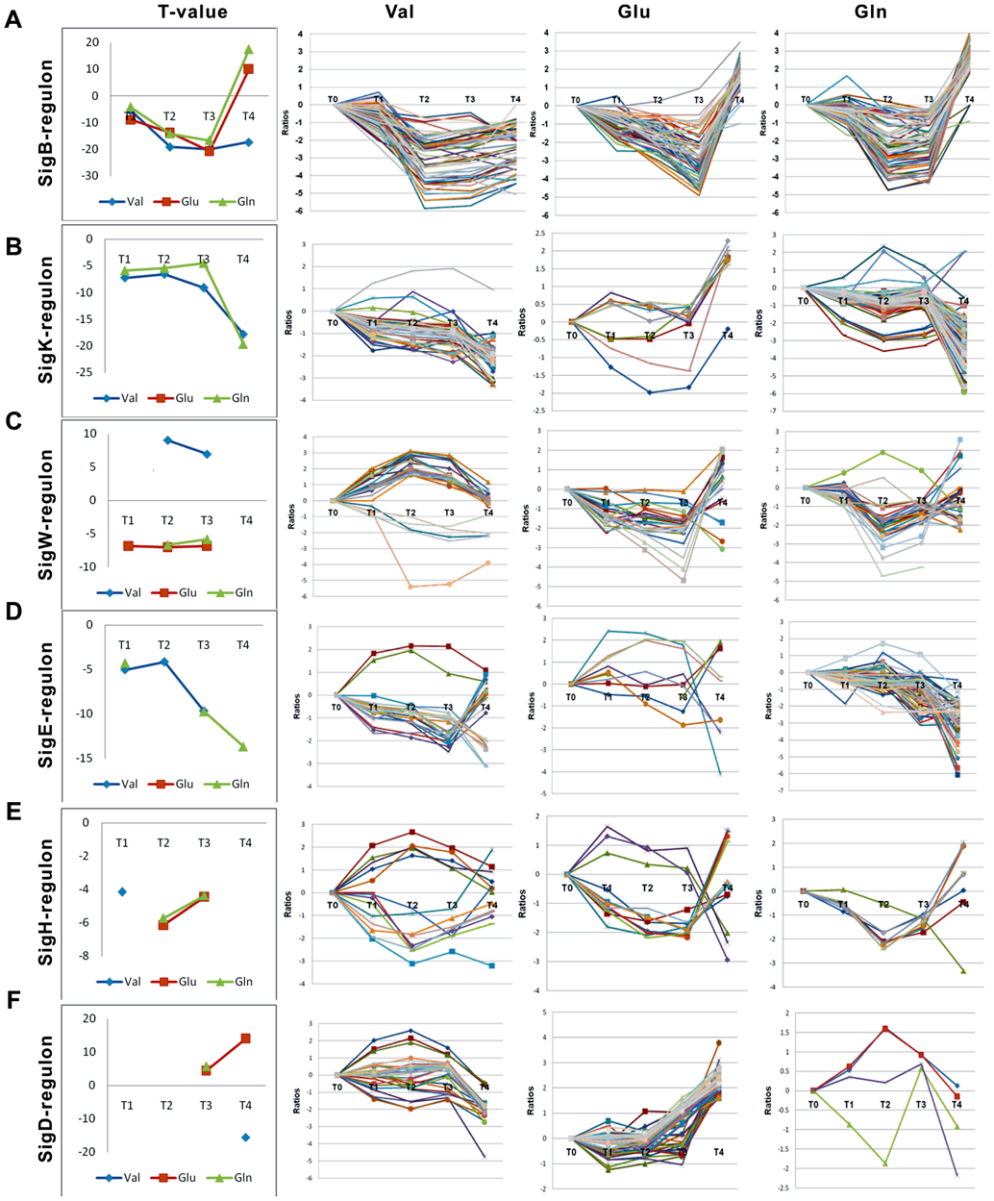
Expression patterns of genes in six sigma factor regulons. SigB-, SigD-, SigK-, SigW-, SigE-, and SigH-regulated genes were overrepresented at one and more time points after addition of valine, glutamate, and glutamine. The *T* values of gene groups regulated by SigB, SigK, SigW, SigE, SigH, and SigD are also shown, which have the significant *T* value (*E*<0.05) in the time course analyzed.

Groupwise analysis using T-profiler yielded the significant values for the Sig-regulated gene groups (Table 2 and Fig. 3) to three amino acid pulses. T-value data revealed that SigB-, SigW, and SigD-controlled genes had similar temporal expression pattern after Glu and Gln exposure, the expression of genes dependent on SigK and SigE were significantly repressed by Val and Gln. Indeed, this observation is further supported by threefold-regulated genes of these Sig-regulons (Fig. 3). SigB, known to control general stress response, coordinates the expression of at least 150 genes in *B. subtilis*. Members of this regulon are transiently induced following heat shock; salt, ethanol, or acid stress; or limitation of glucose, phosphate, or oxygen [29]. In this study, we found the 52 genes of SigB-regulon were significantly repressed by valine. The *gsiB* gene, which code for a general stress protein, displayed the strongest repression ratio (ca. 58 folds). The 61 and 69 genes of the SigB-regulon were differently down-regulated following Glu and Gln pulse, respectively. The repression of SigB-regulon may be explained by the counteracting the nutrient limitation after amino acids addition as carbon and nitrogen source.

**Table 2:** Sigma factors that regulate operon expression (three-fold) in response to amino acid pulses.

Sigma factor	No. (%) of differentially expressed operons by Valine	No. (%) of differentially expressed operons by Glutamate	No. (%) of differentially expressed operons by Glutamine
sigA	99/335 (29.6%)	111/335 (33.1%)	130/335 (38.8%)
sigB	34/67 (50.7%)	46/67 (68.7%)	50/67 (74.6%)
sigW	17/34 (50%)	16/34 (47.1%)	20/34 (58.8%)
sigD	11/30 (36.7)	18/30 (60%)	3/30 (10%)
sigX	5/15 (33.3%)	5/15 (33.3%)	5/15 (33.3%)
sigE	15/83 (18.1%)	8/83 (9.6%)	48/83 (57.8%)
sigF	8/30 (26.7%)	7/30 (23.3%)	16/30 (53.3%)
sigG	6/61 (9.8%)	8/61 (13.1%)	32/61 (52.4%)
sigH	7/24 (29.2)	9/24 (37.5%)	7/24 (29.2%)
sigK	23/59 (39.0%)	5/59 (8.5%)	45/59 (76.3%)
sigL	2/6 (33.3%)	0/6 (0)	2/6 (33.3%)

In *B. subtilis*, the sigma factors SigE, SigF, SigG, SigK, and SigH are related to sporulation and germination [30], [31]. The significant negative *T*-values of the regulons of these sigma factors after Val and Gln addition showed the clear repress of the genes dependent on the sigma factors. Upon considering threefold-regulated genes, as is done in Fig. 3, same results were found. Indeed, most genes of SigK- and SigE-regulons were down-expressed. Partial repression of the sporulation regulons indicated that the full sporulation programme was inhibited by valine and glutamine. We also found a clear downregulation of the KipR-regulated genes by valine and glutamine, which may indicate the release of the “brake” on the sporulation-regulatory cascade. KipR is the negative regulator of the *ycsFGI-kipIAR-ycsK* operon. KipI is an inhibitor of KinA, the primary kinase in the phosphorelay necessary for the phosphorylation of the key transcription factor Spo0A that regulates the initiation of sporulation. The response of SigH-regulated genes to valine has two groups: one group of up-expressed genes (*spo0M*, *minC*, *mre* operon) was regulated only by SigH; one group of down-expressed genes (*racA, dacF, ytx* and *ure* operon) was regulated by SigH and other transcript factor jointly. Similarly, genes (*phrF, rapF, rapC*) regulated by the combination of SigH and ComA exhibited the distinct expression pattern with other genes of SigH regulon after glutamate addition.

Amino acid availability can repress the onset of sporulation and the development of competence [15]. Indeed, the gene groups regulated positively by the key competence regulators ComA and ComK show the significant *T* values at late stage after treatment with glutamate and glutamine. The *comK* gene encoding the competence transcription factor that activates the expression of late competence genes involved in DNA-binding and uptake and in recombination. This observation is further supported by the significant repression of 21 genes in ComK-regulon at 24 h after glutamine addition, including *comK* itself, *comFAB, comEABC, comGA*, and *comC*. Furthermore, all genes of ComA*-*regulon (*comS, pel, degQ, rapC, srfA, rapA-phrA, rapF-phrF*), involved in late competence genes and surfactin production exhibited an approximately 8-fold decreased mRNA level at 24 h after glutamate addition. Two-component signal transduction systems of Rap-Phr were the quorum-sensing systems of *B. subtilis*. [32]. *RapA-phrA* operon controlled by ComA and Spo0A was significantly down-regulated by three amino acids in this study, and inactivated post-exponential-phase gene expression and sporulation indirectly through Spo0A. These results are in agreement with previous observations that competence is repressed by the addition of amino acid mixtures during exponential growth of *B. subtilis* in minimal medium [33].

There are 153 genes related to sporulation in DBTBS database, 37, 17, and 83 genes of among them showed clear repession after Val, Glu, and Gln treatments, respectively (See Text S2). Some genes that encode products with functions in spore and sporulation (*sps, cot, spoVAABCDEF, spoIIIAABCDEFGH, cge, ssp* operons) exhibited significantly lower mRNA levels after Val and Gln addition. The sporulation master regulator Spo0A plays a central role in the initiation of sporulation [34]. The Spo0A-regulated genes with threefold change were very different in response to Val, Glu, and Gln. Two operons *skf* (sporulation killing factor) and *sdp* (sporulation delay protein) were activated by Spo0A. The *skf* operon, *dlt* operon, and *yvaX* gene exhibited significantly higher expression levels immediately after valine addition. The *dlt* operon (D-alanyl-lipoteichoic acid) is responsible for D-alanine esterification of both lipoteichoic acid (LTA) and wall teichoic acid (WTA). However, *sdp* operon was repressed after Gln addition. The *fla-che* operon regulated negatively by Spo0A, involved in motility/chemotaxis, also displayed significantly different expression levels: repression with Val and induction with Glu. These results indicated that three amino acids have the distinct effect and mechanism on sporulation. The data demonstrated that the effect of amino acid preventing *B. subtilis* to enter the sporulation process was glutamine>valine>glutamate. We found the KipR-regulated genes were repressed by Val and Gln, induced by Glu, which may indicate the different effects on the sporulation-regulatory cascade. In agreement with these results, Ulrike Mäder *et al.* [15] described a negative effect of amino acids on expression of early-stage sporulation genes. They reported that addition of CAA (Casamino Acids) to minimal medium affects the transcription of genes of competence, early-stationary-phase and sporulation.

The expression of genes dependent on GerE, involved in late spore coat genes and germination, also produced significant *T-* values after 24 h of valine and glutamine addition. The repression of the GerE-dependent genes (20 genes and 31 genes with respect to Val and Gln) was shown in Text S2. Only ribonuclease III gene (*rnc*) and one spore coat protein gene (*cotC*) were induced by valine and glutamine, respectively. Three genes (*cotA, cgeD, cgeE*) were overrepresented by glutamate. Furthermore, glutamate displayed less effect on the process of sporulation and germination compared with valine and glutamine.

**Figure 4:**
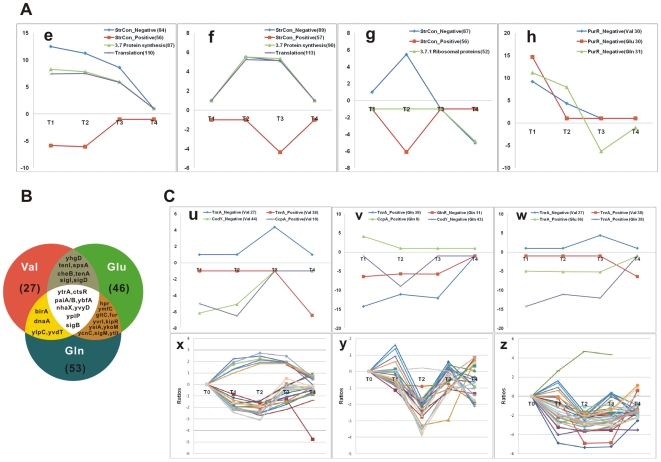
The TFs responding to amino acid pulses. (A) Repression of the stringent response in B. subtilis as revealed by T-profiler. The T values of gene groups negatively (diamond) and positively (square) controlled by RelA and involved in translation (cross) and protein synthesis (triangles) are shown after Val (e), Glu (f), and Gln (g) pulses. (h) Induction of purine and pyrimidine synthesis gene groups. The T values of gene groups regulated negatively by PurR after Val (diamond), Glu (square), and Gln (triangles) treatments. A distinction is made between groups of genes that are positively or negatively regulated by the transcription factor mentioned (indicated by Pos. and Neg., respectively). The T values were set to 1 or −1, if E-values>0.05. (B) Venn diagrams showing the number of regulated TF genes after treatment with Val, Glu, and Gln. The numbers represent a list of non-redundant genes with threefold induction or repression in response to Val, Glu, or Gln. (C) (u) The T values of gene groups regulated by TnrA positively (squares) and negatively (diamonds), by CodY negatively (triangles), and by CcpA positively (crosses) are shown after Val addition. (v) The T values of gene groups regulated by TnrA positively (diamonds), by CodY negatively (crosses), GlnR negatively (squares), and by CcpA positively (triangles) are shown after Gln addition. (w) The T values of gene groups regulated by TnrA after Val (squares and diamonds), Glu (triangles), and Gln (crosses) treatments. A distinction is made between groups of genes that are positively or negatively regulated by the transcription factor mentioned (indicated by Pos. and Neg., respectively). CodY-regulated genes after Val (x) and Gln (y), TnrA-regulated genes after Gln (z) are shown. The T values were set to 1 or −1, if E-values>0.05.

T-profiler analysis also showed a strong repression of genes involved in RelA-dependent after amino acid additions (Fig. 3). The significant *T-*values for both (RelA-dependent) positive and negative stringent control gene groups were found (Table S3). The (RelA-mediated) stringent response was restrained by these amino acids as nutrients. The significant upregulation of gene groups involved in translation and protein synthesis (Fig. 4A), as well as purine and pyrimidine synthesis, further reflects the repression of the stringent response.

The SigW regulon of *B. subtilis* has been functionally implicated in cell-wall-associated processes and the adaptation to alkaline shock, salt shock, phage infection and certain antibiotics that affect cell wall biosynthesis [30], [35]. The genes were mainly involved in transport processes and detoxification. SigW-regulon to Val showed the positive *T*-value. Based on the definition of the SigW regulon structure of Huang *et al.* [36] and Wiegert *et al.* [37], we found 22 of the 62 SigW regulon members to be significantly induced (at 30 min and 2 h) after Val addition, whereas *yfh* operon, *yxjJ, spxA* genes to be repressed; 23 and 28 genes to be down-expressed after Glu and Gln addition. Most of these overrepresented genes are unassigned-function genes excepting *xpaC, pspA, yndN, sppA, pbpX, spxA, pbpE, racX, sigW* genes. We also observed the significant induction of genes regulated by YvrH after treatment with valine and glutamate in T-profiler analysis. The YvrG-YvrH two-component system appears to be related to cell membrane and cell wall function. Furthermore, SigX sigma factor relating to the cell surface homeostatic functions, was regulated by this system. Masakuni Serizawa *et al*. noted that the *yvrGHb* null mutant showed the unusual autolysis and higher susceptibility to the four kinds of antibiotics (aztreonam, cefepime, bacitracin, and fosfomycin) [38]. Indeed, *sunT, sunA*, *wapA-yxxG*, and *dlt* genes were upregulated, while *yvrI, sigX*, and *lytA* genes were downregulated (Text S2). On the other hand, *sigI* gene, which is a member of the class *VI* heat shock genes of the *B. subtilis* heat shock stimulon, encodes an alternative sigma factor of the σ^70^ family. Recent study reported that one function of SigI is related to the maintenance of cell envelope integrity and homeostasis during heat stress [39]. In this study, *sigI* gene was also induced by Val and Glu. These data demonstrated that valine and glutamate activated some genes involved in cell membrane and cell wall function.

SigD regulates genes related to flagellar synthesis, motility, chemotaxis, and autolysis. 31 genes of SigD-regulon were differently expressed at one or more time points after Val pulse. 24 of these genes, involved in motility/chemotaxis (*motAB, hag, fliDST, fla/che* operon, *mcpABC*), were significantly repressed; three genes, involved in autolysis (*dlt* operon), were immediately up-expressed in response to Val. 52 genes of SigD-regulon were differently induced at 24 h following Glu addition, related to motility/chemotaxis (*motAB, hag, fliDST, fla/che* operon, *mcpABC*). Only four SigD-regulated genes (*hag, dltC, dltB, lytA*) displayed significant changes after Gln addition (see Text S2). It was previously found that *B. subtilis* showed chemotaxis toward many amino acids [40]–[42]. Recently, Márquez-Magaña *et al.* demonstrated that the expression of flagellar genes was repressed by the addition of CAA (a complete hydrolysate of casein) or a mixture of mono-amino acids in *B. subtilis*. The authors suggested that CodY, binding specifically to *hag* and *fla/che* promoter, was a nutritional repressor of the gene expression by sensing intracellular GTP levels [43], [44]. However, the effect of the different mono-amino acid on motility is yet to be known.

The data analysis in this study demonstrated that one of the major classes of genes differentially expressed under the treatment with valine and glutamate were those involved in motility, flagellar synthesis, and chemotaxis. Indeed, the results suggested that the amino acids exert their different effects on the expression of genes involved in motility: valine showed a strong repression of ciliary/flagellar genes (adjusted P-value 10^−14^) and chemotaxis genes (adjusted P-value 10^−6^); glutamate showed a clear induction of ciliary/flagellar genes (adjusted P-value 10^−25^) and chemotaxis genes (adjusted P-value 10^−11^). In agreement with the *hag-lacZ* study, transcription of the *hag* gene was three-fold reduced in cells grown after BCAA (Val, Leu, or Ile) addition. The GTP-binding protein CodY appears to repress flagellin gene expression in response to the availability of amino acids [44].

No obvious expression changes of *codY* itself after valine, glutamate, and gltamine treatment were observed. CodY-mediated reduction of SigD-regulon expression might be mainly attributed to the function of its effectors BCAAs (not GTP) in response to valine. As described above, the amino acids additions significantly induced PyrR-regulon and PurR-regulon genes, and reduced PucR-regulon genes. The results adjusting these genes expression levels maybe lead to biosynthesis of cellular nucleotide pools. Although intracellular GTP concentration was not determined, possible increase of GTP can be expected after three amino acids addition. The availability of three amino acids did not displayed similar impact on expression of SigD-regulon. These results revealed the obvious CodY-mediated reduction may result from its effector BCAA, not from its effector GTP. Strikingly, the response of these genes to glutamate differed from the pattern of valine in that their expressions were clearly induced. To our knowledge, glutamate represents the first example showing induction of genes involved in motility after amino acid addition. The exact roles of glutamate in conferring transcriptional regulation of motility remain mysterious, and should be further investigated.

Interestingly, we observed major changes in *motAB* and *ytxDE (motPS)* genes involved in rotary motor of bacterial flagellum upon amino acids addition. In bacterial setting the dual motility systems appear to optimize motility under different conditions. Two stator-force generators (MotAB and MotPS) competitively combine with their single FliG partner in the flagellar rotor switch element, to create a proton channel that drives rotation of the flagellum. Recently, It is hypothesized that the interactions of MotAB are optimized for interactions with the single FliG rotor protein relative to those of MotPS, the MotPS make a contribution to motility at high pH, Na^+^, and viscosity when MotAB is also present [45]–[47]. Inspection of the response pattern of *motAB* and *ytaDE* genes revealed two hints: (i), MotAB and MotPS not only competitively combine with their single FliG partner at protein level, but also display antagonistic relationship at transcriptional level; (ii) valine and glutamine stimulated transcription of Na^+^-coupled MotPS, and suppressed transcription of H^+^-coupled MotPS, whereas glutamate exert the converse effect on transcription of motor proteins.

The *B. subtilis* genome probably encodes 317 transcription factors or transcriptional regulators (http://dbtbs.hgc.jp/). TFs are of special interest since they are capable of coordinating the expression of several downstream target genes and, hence, entire metabolic and developmental pathways. Of the approximately all potential TFs on the BSU microarray, 27, 46, and 53 showed marked (three-fold) changes in transcript abundance (Text S2) in response to three amino acids (Val, Glu, and Gln). The largest set of regulated TF genes was those changed by Gln (53 TF genes; Fig. 4B). 20 TFs were changed by both Glu and Gln, and 16 TF genes were regulated by both Val and Glu. A group of 9 TF genes was mutually overrepresented by treatment with all three amino acids, including *ytrA, ctsR, paiA, paiB, yplP, ybfA, nhaX, yvyD, sigB*. A more detailed discussion of the transcription factors is provided in the Text S2.

Genomic sequencing of various microorganisms has revealed the presence of many two-component regulatory systems in every species. In *B. subtilis*, 36 sensor kinases and 35 response regulators have been found, among which each of 30 kinase-regulator pairs resides in an operon on the genome. The addition of amino acids displayed slight effect on the expression of these genes. We observed repression of chemotaxis TCS (*cheA, cheB, cheW*) and *citST*, and *ybdKJ* and *yocF* were clearly induced by valine, whereas *resE* and *cheY* also exhibited a moderate induction, but did not fulfil the threefold threshold. The *citST* was involoved in transport of divalent metal ions/citrate complexes (*citM*), and was repressed in the presence of glucose by the general transcription factor CcpA. The signals regulating the kinases *ybdK* and *yocF* are still unknown. It was reported that *ybdK* of the putative *ybdJK* two-component system was regulated by ComK, which suggests that the YbdJ response regulator might affect competence-specific gene transcription [48]. Yasutaro Fujita *et al.* found that YbdJ upregulated *ybdJ*, (*ybdK*), *purC, purN, purH, ald*, and downregulated *ykoM, ysfC, ysfD* [49]. The YocFG (DesKR) system was reported to be involved in thermosensing and signal transduction at low temperatures, and DesR was responsible for cold induction of the *des* gene coding for the Δ5-lipid desaturase [50]. The glutamate clearly induced chemotaxis TCS (*cheA, cheB, cheY, cheV*, and *cheW*) gene and quorum-sensing sytem *comX* gene. None of the genes encoding TCS systems induced or repressed after treatment with Gln was found using threefold criterion.

### Effects of three amino acids on metabolism

The CodY-, CcpA-, TnrA- and GlnR-regulated gene groups displayed the significant *T* values (Fig. 4C). CodY, CcpA, and TnrA are three important global regulatory proteins involved in carbon and nitrogen metabolism in *B. subtilis* [51]–[52]. In this study, the cell adjusted carbon and nitrogen metabolism to redistribute the fluxes of carbon and nitrogen after addition of Val, Glu, and Gln. Valine as a BCAA activated the global regulator CodY, and negatively regulated gene expression of its regulon. The hypothesis is supported by the significant *T*-value of CodY_Negative gene group (Fig. 4C–u). The conclusion is further confirmed by the significant repression of CodY-regulon genes immediately after treatment with Val (Fig. 4C–x), including *ilvB, ilvA, dpp, ureABC* operons, *ilvD*, and *acsA* genes. However, *bkd* operon regulated by CodY, TnrA, and BkdR was greatly induced, involved in the control of isoleucine and valine utilization and degradation. As a result, valine via regulation of CodY activated genes of the carbon-overflow and BCAAs-degradation pathways, it repressed citric acid cycle, reutilization pathway of acetate, and BCAA biosynthesis. Glutamine revealed the delayed-regulation of CodY-regulon, exhibited the significant *T*-value of CodY_Negative gene group only after 30 min of Gln pulse (Fig. 4C–v). Indeed, *ilvB, ilvA, dpp, ureABC* operons, *ilvD, gabP, ybgE, citB*, and *acsA* genes were repressed at 30 min (T2) (Fig. 4C–y). The significant *T*-values of CcpA_Positive gene group (only *ilvB* and *alsSD* operons, *ackA* gene) showed negative values at 5 min and 30 min following valine addition, and positive value at 5 min following glutamine addition. Upon considering only threefold-regulated genes, some CcpA_negative genes were found. The utilization of carbohydrate pathways, including beta-glucosidic compounds (*licAC, bglPH* operon), xylose (*xylAB*), ribose (*rbsDB*), and gluconate (*gntKP*), were activated immediately after treatment with valine. The *cydABCD* operon, a branched electron transport chain, was induced transiently in the first 5 min, and repressed after 30 min following valine stress. The *lcfA* operon (*lcfA-ysiAB-etfBA*), as a group of genes apparently encoding proteins involved in the putative functions linked to fatty acid metabolism, was down-regulated by Glu and Gln.

The significant *T* values for both TnrA_positive and TnrA_negative gene groups were observed. These results indicated that three amino acids inactivated the regulator TnrA. When glutamine was added, TnrA significantly repressed operons such as *nrgAB, nasBC* and *nasDEF*, the products of which mediate the uptake of ammonium ion and the use of secondary nitrogen sources such as nitrate and nitrite, also down-regulated glutamine ABC transporter (*glnQHMP*), *kipA* operon, *glnAQ, gltA* genes, only activated a function-unassigned gene (*yccC*) (Fig. 4C–z).

#### Central carbon pathway

**Figure 5:**
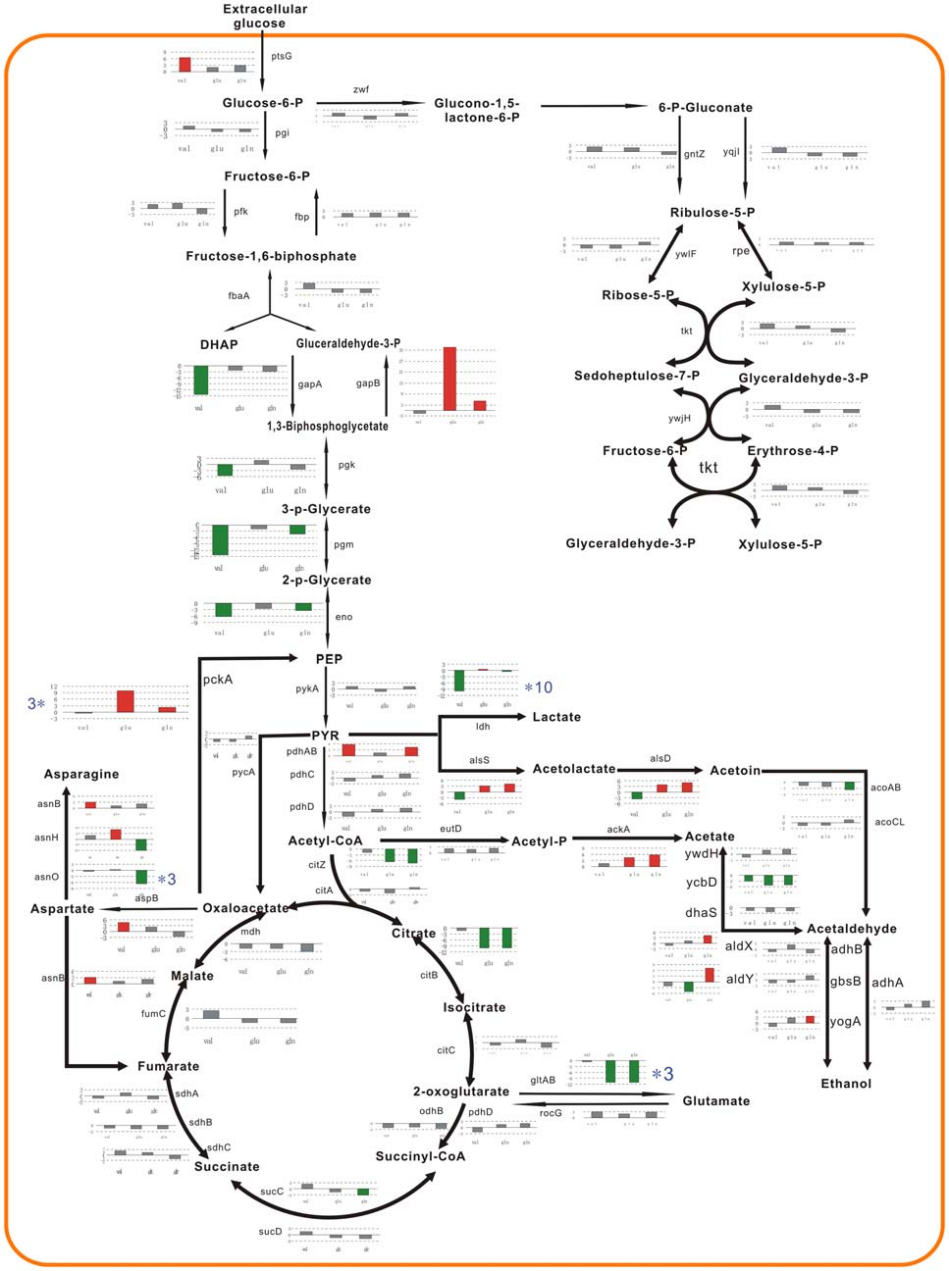
Effects of three amino acids on central carbon pathway. The expression of the genes related to central carbon pathway is schematically presented. Three bars from left to right represent the fold changes of gene expression in response to valine, glutamate, and glutamine, repectively. Red bars represent an upregulation, green bars represent a downregulation, and grey bars refer to messages that are not considered to be significantly changed by our cutoff (three-fold).

According to SubtiList functional classification, the central carbon pathway includes the specific pathways (2.1.1), main glycolytic pathways (2.1.2), and TCA cycle (2.1.3). As shown in Fig. 3B, a total of 30, 48, and 58 genes, involved in the specific pathways were identified as significantly expressed at one or more time points in response to additions of Val, Glu, and Gln, respectively. These genes are mainly involved in the glycolysis, TCA, the overflow pathway (acetoin, lactate, and acetate syntheses), and utilization of carbohydrates and related molecules (Fig. 5).

Valine activated the transport of glucose, whereas inhibited the production of pyruvate. The *gapB* gene encoding the anabolic glyceraldehyde-3-phosphate dehydrogenase in gluconeogenesis pathway was subject to a strong induction of glutamate and glutamine. The pyruvate dehydrogenase encoded by the *pdhABCD* operon links glycolysis to the TCA and overflow metabolism. The addition of valine and glutamine resulted in a fourfold induction of this operon. Under the addition of valine, the *alsSD* operon encoding a-acetolactate synthase and decarboxylase as well as the *lctEP* operon coding for lactate dehydrogenase and permease were obviously repressed, suggesting that pyruvate is specifically catabolized to acetyl-CoA. While, glutamate and glutamine activated acetoin formation, one of the overflow metabolism pathways that serve to excrete excess carbon from the cell. Acetyl-CoA is then the starting point of two alternative pathways: it may be used as a substrate of citrate synthase (*citZ*) to initiate TCA, or it can be converted to acetate. Two CcpC-controlled genes *citZ* and *citB* (encoding aconitase) were repressed by glutamate and glutamine. It suggested that the TCA cycle was downregulated after addition of these two amino acids. Induction of CcpA-controlled *ackA* (encoding acetate kinase) expression was significant under these conditions, and resulted in acetate formation, additional one of the overflow metabolism.

The expression of *alsSD* operon is induced by gluamate and gltamine, and is repressed by valine. *ldh-lctP* operon, encoding L-lactate dehydrogenase and L-lactate permease, some acetate metabolism genes (*ydaP, acsA, ycbD*) were repressed by valine, glutamate, and glutamine. It was reported that the strong induction of YvyD was caused by amino acid starvation [53]. Our transcriptional data appeared to show that *yvyD* and *ytrA* (involved in acetoin utilization) displayed reverse correlation of expression in response to amino acid addition. An intriguing explanation for this result could be that YvyD may be somehow involved in the re-utilization of by-products of carbon-overflow pathways. It is known that the compounds pyruvate and 2-oxoglutarate are particularly important to the main glycolytic pathways and TCA cycle in the cell respectively. Several potential fates of pyruvate, such as the biosynthesis of amino acid, fatty acids, lactate, acetate, and acetyl-CoA for the citric acid cycle, determine the distribution of carbon flux among these pathways. Valine and glutamine obviously activate the flux from pyruvate to acetyl-CoA through overexpression of pyruvate dehydrogenase genes (*pdhAB*) at 5 min and 30 min, and suppress the production of pyruvate from glucose through underexpression of the *gap* operon of CggR-regulon at 30 min. We did not observe an effect of glutamate on the expression of *pdhAB* genes and *gap* operon. The *pckA* and *gapB* genes involved in gluconeogenesis are repressed by CcpN in glycolysis conditions. The two genes repression is released at 30–120 min by glutamate (30–34 fold) and glutamine (6-5 fold). It would be interesting to bridge the linkage between derepression of CcpN and glutamate/glutamine. The CcpA- and CcpC-controled gene citZ and CcpC-, AbrB-, CodY-, TnrA-controled gene citB, encoding the first two enzymes genes of the citric acid cycle (citrate synthase and aconitase), were also repressed at 30-120 min by glutamate and glutamine. *citB* will be repressed strongly by the combined activities of CcpC and CodY, and induced by AbrB and TnrA. It is intriguing that the addition of valine did not display repression effect on the expression of *citB* gene. The genes (*mdh, ywaK*, and *yjmC*), encoding three malate dehydrogenase isoenzymes, seem to be differently regulated by glutamine. Three amino acids exerted the different effects on the central carbon pathway. The results were shown in Fig. 5.

#### Glutamate/Glutamine metabolism and nitrogen metabolism

In *B. subtilis*, two transcriptional factors, TnrA and GlnR, control gene expression in response to nitrogen availability. TnrA activates and represses gene transcription when nitrogen is limiting for growth, while GlnR represses gene expression during growth with excess nitrogen. If glutamine as the favoured nitrogen sources is available, ThrA represses operons such as *nrgAB* (encoding GlnK-AmtB system), *nasA, nasBC, nasDEF*, the products of which mediate the uptake of ammonium ion and the utilization of the secondary nitrogen sources nitrate and nitrite. As expected, the addition of glutamine significantly downregulates TnrA-controled genes *nrgAB*, *nasA*, *nasBC*, and *nasD* immediately. However, *narGHJI* operon of Fnr-regulon, encoding the differentially regulated nitrate reductase is slightly induced (about 3.5 folds) at 5 min by glutamine. Fnr is a global transcription regulator that activates the expression of genes encoding many of the enzymes required for the anaerobic environment. The phenomena suggest that the low oxygen tension resulted from rapid growth maybe generate the anaerobic induction of Fnr-regulon genes.

**Figure 6:**
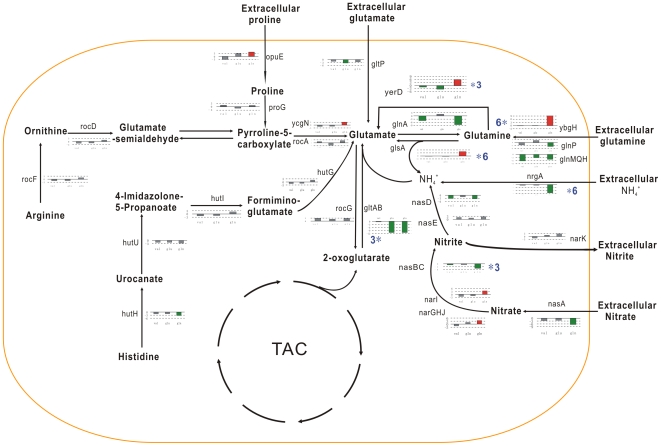
Effects of three amino acids on metabolism of nitrogen. The expression of the genes related to the utilization and metabolism of nitrogen is schematically presented. Three bars from left to right represent the fold changes of gene expression in response to valine, glutamate, and glutamine, repectively. Red bars represent an upregulation, green bars represent a downregulation, and grey bars refer to messages that are not considered to be significantly changed by our cutoff (three-fold).

As shown in Fig. 6, the glutamate synthase GOGAT (*gltAB* operon), glutamine synthase GS (encoded by *glnA*), and glutamate dehydrogenase GDH (RocG) play an important role in glutamate/glutamine metabolism and nitrogen metabolism, and tightly are dual control by the carbon and nitrogen signalling systems. The regulation of the *gltAB* operon is complex and involves directly two regulatory proteins TnrA and GltC. Valine, glutamate, and glutamine display the different repressive effect on the expression of *gltAB* gene with 3-fold (at 5 min), 11/48-fold (at 30 and 120 min), and 20-fold (at 30 and 120 min), respectively. Expression of *gltC* and *tnrA* also is down regulated after glutamine addition.

The expression of *glnA* gene is regulated by two transcriptional factors, TnrA and GlnR. The signal molecule, glutamine, is an effector of TnrA and GlnR. Recently the molecular mechanism of modulation of TnrA and GlnR activity mediated by glutamine has been elucidated [54], [55]. It is observed that expression of *glnRA* operon immediately is repressed after glutamine addition. Glutamine is now known to be metabolized not only by glutamate synthetase encoded by *gltA* but also by glutaminase encoded by *glsA*. The *glsA*-*glnT* (*ybgJH*) operon is greatly upregulated with 25/50-fold instantly after glutamine addition. Besides GlnT involved in the uptake of glutamine, *B. subtilis* contains an ATP-binding cassette transporter system for glutamine, encoded by *glnQHMP* operon. Although *glnT* gene is significantly induced by glutamine, *glnQHMP* operon controlled by TnrA is repressed. *B. subtilis* maybe possess some unidentified glutamine transport and utilization systems other than the GlnQHMP/GlnT and GlsA/GltT.

#### Amino acid metabolism

Previous studies suggested regulation by amino acid availability for the amino acid biosynthesis operons [15]. Indeed, the addition of valine, glutamate, and glutamine exhibited the strong effect on the amino acid metabolism. There are 129 genes related to metabolism of amino acids and related molecules in DBTBS database, 57, 42, and 84 genes of among them showed significant expression after Val, Glu, and Gln treatments, respectively.

The operons (*aroA, trp, aroFBH*), involved in the aromatic amino acids synthesis from erythrose-4-phosphate and phosphoenolpyruvate through chorismate, were repressed at 5–30 min after valine addition. Glutamate and glutamine only suppressed the tryptophan biosynthesis genes *trpABCDEF* from chorismate with 30 min-delay. Many genes regulated by Glu and Gln displayed the similar phenomenon of delay. It demonstrates that valine has more direct and straightaway function on gene expression compared with glutamate and glutamine. The transcription of the *trp* operon is regulated by transcription attenuation with MtrB (TRAP) and AT proteins, in response to the availability of tryptophan, and charged tRNA^Trp^ [56]. The T-profiler analysis clearly revealed the significant *T* values for MtrB_Positive gene groups after Val, Glu, and Gln additions. It is intriguing that only tryptophan biosynthesis operon was accordantly downregulated by all three amino acid. However, the significant change on expression of *at* operon and *mtrB* gene was not observed. Besides transcription attenuation, *trp* operon might be rigorously regulated and possibly linked with other amino acids availability.

*B. subtilis* has four transcription units that are devoted to BCAA biosynthesis: *ilvA*, *ilvD*, *ybgE* genes and *ilv-leu* operon. It is known that the *ilv-leu* operon is elaborately regulated by the global regulators of cellular metabolism (CcpA, CodY, TnrA and RelA) according to the cellular energetic and nutritional conditions through the intracellular concentrations of the signal compounds, such as FBP, GTP, BCAAs, and glutamine. Thus, the regulation of BCAA biosynthesis most likely plays a central role to link catabolism to anabolism in the overall metabolism of *B. subtilis* [57]. As expected, valine activated CodY, and immediately repressed the *ilv-leu* operon at 5–30 min. Glutamine, as the favoured nitrogen source, inactivated TnrA, and maybe resulted in derepression of the *ilv-leu* operon [58]. However, we observed that glutamine represses the expression of the *ilv-leu* operon at 30 min in this study. *ilvA*, *ilvD*, *ybgE* genes are also repressed by valine and glutamine, probably by indirect mechanisms. *hom* operon, involved in the biosynthesis of aspartate (the precursor molecule of isoleucine) from L-aspartate semi-aldehyde, is downregulated at 30 min by valine and glutamine. The *bkd* operon, involved in the degradation of BCAAs and biosynthesis of lipids, is rigorously regulated by BkdR, CodY, and TnrA. Valine activates the expression of *bkd* operon through CodY at 5–120 min. Some genes, involved in the arginine systhesis and urea cycle, were regulated by three amino acids: the arginine systhesis from glutamate to ornithine to arginine was activated at 5 min, whereas urease genes (*ureABC*) were suppressed.

In summary, the BCAAs, Phenylalanine, tyrosine, tryptophan, and glutamine biosynthesis gene were immediately downregulated, while asparagine synthetase gene (*asnB*) and the S-box-regulated transcriptional units, involved in sulfur and methionine metabolism, were immediately upregulated after addition of Val. Arginine biosynthesis, S-box-regulated units, and glycine cleavage system were induced, whereas glycine oxidase gene and glutamate synthase genes were repressed after treatment with glutamate. However, arginine biosynthesis and utilization (*argCJB, rocB*), BCAAs, phenylalanine, tyrosine and tryptophan biosynthesis, histidine metabolism (hutH), asparagine synthetase (*asnOH*), glutamine synthetase *(glnA)*, and glutamate synthase (*gltA*) gene were repressed, whereas S-box-regulated units, and glycine cleavage system genes were induced after treatment with glutamine. Fig. S1 depicted the effects of valine, glutamate, and glutamine on the metabolism of twenty amino acids.

#### Cysteine/Methionine metabolism and sulfur metabolism

**Figure 7:**
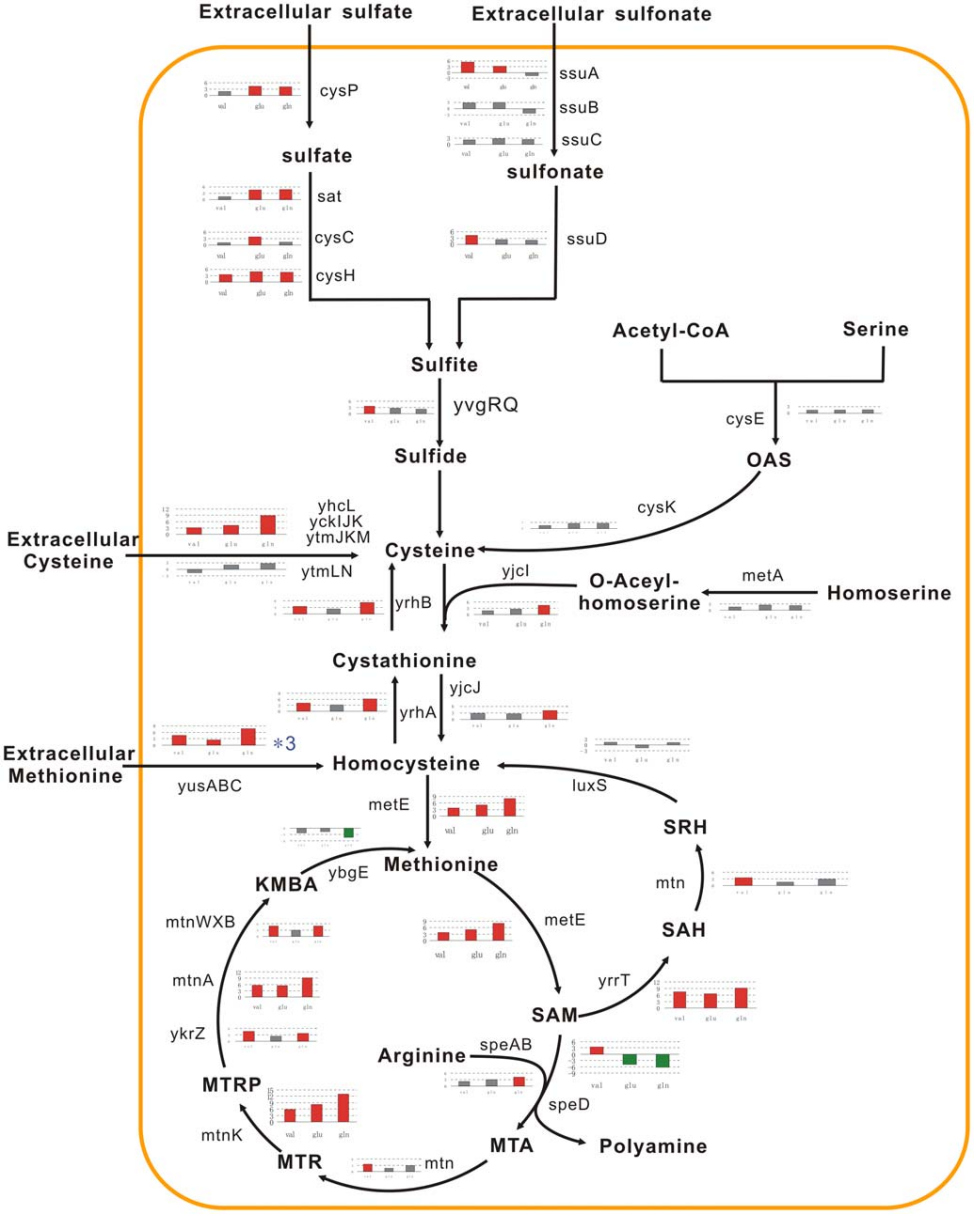
Effects of three amino acids on metabolism of sulfur. The expression of the genes related to transport and metabolism of sulfur is schematically presented. Three bars from left to right represent the fold changes of gene expression in response to valine, glutamate, and glutamine, repectively. Red bars represent an upregulation, green bars represent a downregulation, and grey bars refer to messages that are not considered to be significantly changed by our cutoff (three-fold).

Sulfur is a crucial atom in cysteine and methionine (Met), as well as in several coenzymes and cofactors such as thiamine, biotin, or coenzyme A (CoA). In this study, we observe that three amino acids all activate the sulfur metabolism, including the assimilation of sulfur, biosynthesis of cysteine and methionine, and methionine salvage process (Fig. 7 and Text S2). The genes, involved in the assimilation of sulfur, mostly are upregulated a certain extent after addition of the three amino acids. The *yusABC* and *yxeMNO* genes, related to the methionine uptake system, is most significantly activated (10–20 folds). Among three amino acids, glutamine exhibits the strongest active effect on assimilation of sulfur.

*B. subtilis* has two major cysteine biosynthetic pathways: the thiolation pathway requiring sulfide and the reverse transsulfuration pathway, which converts homocysteine to cysteine with the intermediary formation of cystathionine. Although the production of sulfide from the uptake and reduction of sulfate (*cysP, cysC, cysH, sat genes*) is activated, three amino acids do not significantly induce the thiolation pathway (*cysK, cysE*). The genes *yrhA* and *yrhB*, involved in reverse transsulfuration pathway from homocysteine to cysteine, are upregulated by valine (3.5–4 folds at 5–30 min), glutamate (3 folds at 30 min), and glutamine (6 folds at 30 min).

*B. subtilis* has three major Met bioavailable pathways: the biosynthesis by transsulfuration pathway, Met salvage pathway, and SAM recycling pathway. The genes *metI* (*yjcI*) and *metC* (*yjcJ*), involved in transsulfuration pathway are induced at 30–120 min by only glutamine. Three amino acids all activate Met salvage pathway (*speD, speE*, *ykrTS* (*mtnKA*) and *ykrWXYZ (mtnWXBD)* operons, and SAM recycling pathway (*mtn, yrrT, metE*) at 30 min.

YrzC (now renamed by CymR) has been identified as a master transcription regulator in cysteine metabolism and sulfur metabolism. YrzC regulator negatively controlled the expression of *ytmIJKLMNO-ytnIJ-ribR-ytnLM, yrrT-mtn-yrhABC, yxeKLMNOPQ, ssuBACD-ygaN* operons, and *ytlI, cysK, yhcL(tcyP), ydbM* genes [59], [60]. In this study, the significant T-values of YrzC_Negative group genes indicated that three amino acids may have the effect upon cysteine metabolism and sulfur metabolism. Indeed, this observation is further supported by the significant induction of 4 out of 7 operons (*yrrT-mtn-yrhABC, yxeKLMNOPQ, ssuBACD-ygaN, yhcL*) by valine and the induction of 6 operons (but *ydbM*) by glutamine and glutamate (Fig. S2). The YrzC-dependent genes shared the similar expression pattern (clear and transient induction at 30 min) after treatment with Glu and Gln, indicating same regulatory effect of the two amino acids. Valine displayed the different effect on expression of YrzC-regulon: the *yxe* operon (encoding products resembling proteins that function in organic sulfonate and methionine uptake) and *yrrT-mtn-yrhABC* operon (involving in cysteine synthesis from S-adenosylmethionine through intermediates S-adenosylhomocysteine, ribosylhomocysteine, homocysteine, and cystathionine), were immediately and obviously up-regulated; *ydbM* gene, encoding a protein similar to butyryl-CoA dehydrogenase, was repressed. YrzC may be considered as a repressor controlling several pathways leading to cysteine biosynthesis, including the OAS-thiol-lyase (CysK), L-cystine transporters (YhcL and YtmJKLMN), sulfonate assimilation (SsuABCD), and the methionine-to-cysteine conversion involving YrhA and YrhB [61]. As revealed from these data in Fig. S2, expression of genes related to sulfur, methionine and cysteine metabolism was affected by three amino acids availability. The amino acids upgraded the sulfur flux from sulfite (including sulfite assimilation and uptake) and homocysteine to cysteine as well as the sulfur flux from SAM and homocysteine to methionine. Two major cysteine biosynthetic pathways both were intensified: the thiolation pathway requiring sulfite and the reverse transsulfuration pathway, which converts homocysteine to cysteine. Glutamine presented the induction of both pathways on the conversion of methionine-cysteine each other.

#### Phosphate metabolism

*B. subtilis* has two major systems of phosphate transport: a low-affinity phosphate uptake system (*pit* gene) and a high-affinity phosphate transport system (*pst* operon). In some organisms, a high expression of the PstS protein occurs under stress conditions, including alkali-acid conditions, the addition of subinhibitory concentrations of penicillin, and the response of pathogenic bacteria to the eukaryotic intracellular environment. These observations suggest that PstS would be one of the multi-emergency proteins that help cells to adapt to growth in different environments [62]. Valine, glutamate, and glutamine induced the expression of *pst* operon (Text S2) at 5 min, 30 min, and 24 h, respectively. In some organisms, such as *S. lividans* and *S. coelicolor*, certain carbon sources (fructose, galactose or mannose) can activate the expression of *pst* operon [63]. The *pit* gene also was up-regulated by three amino acids. In addition, all the *ybcPST* and *ybdABDE* genes of *skf* operon regulated by PhoP, Spo0A, and AbrB diplayed significant and transient (5–30 min) induction immediately after valine pulse, and produced the sporulation killing factor to lyze sister cells; all the *tuaABCDEFGH* genes of *tua* operon diplayed significant and transient (30–120 min) induction after glutamate pulse, and produced teichuronic acid. In *B. subtilis*, *ybhH* gene of SigE-regulon, encoding a sporulation protein, was induced during phosphate deprivation [64]. In this study, *yhbH* gene was clearly repressed after treatment with valine and glutamine. These combined data revealed the obvious induction of some genes of PhoP-regulon may not result from phosphate starvation, may exist a connection between phosphate and amino acid regulation, which should be further investigated.

#### Nucleotide metabolism

There are three transcriptional regulators PyrR, PucR, and PurR, involved in nucleotide metabolism. The pathway responsible for the de novo synthesis of pyrimidines was encoded by PyrR/PurR-controlled *pyr* operon. The *pyr* operon, containing 10 genes, was slightly induced at 5 min after amino acid addition (Fig. S3). The expression of these genes was regulated by PyrR, PurR and cellular nucleotide pools.

The de novo synthesis of purine nucleotides is carried out by pathways encoded by *pur* operon. PurR, as a repressor of purine biosynthesis, inhibits transcription of its regulon genes *purR*, *pur*, *glyA*, *guaC*, *ytiP(pbuO)*, and *pbuG*, and *pur*, *nusB-folD*, *pyr*, and *xpt-pbuX* operons. The *pbuX*, *pbuE* (*ydhL*), *nupG* (*yxjA*), and *pbuG* genes encode purine transporter. The *purR* gene did not exhibited obvious change of expression. However, *pur* operon and *pbuX* gene of its regulon were significantly induced immediately at 5 min after treatment of three amino acids. Indeed, T-profiler analysis showed a strong induction of genes of purine biosynthesis at 5 min after amino acid additions (Fig. 5A–h). The significant *T* values for Pur_Negative gene groups were found. Interesting, these genes exhibited the different expression pattern at 30–120 min in response to valine, glutamate, and glutamine. The hypoxanthine-guanine permease *pbuG* was also induced by glutamate and glutamine. Purine efflux pump gene *pbuE* was repressed. Similarly, the results adjusting these genes expression levels leads to enlargement of cellular nucleotide pools for rapid growth.

**Figure 8:**
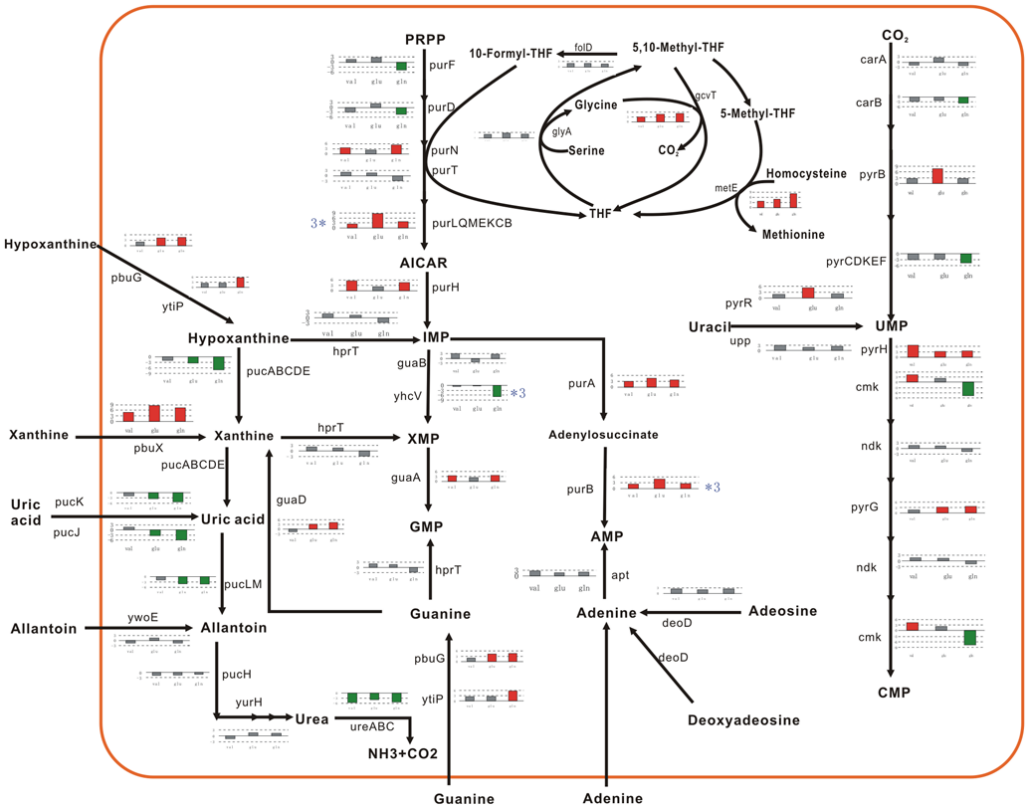
Effects of three amino acids on metabolism of nucleotide. The expression of the genes related to the salvage and metabolism of nucleotides is schematically presented. Three bars from left to right represent the fold changes of gene expression in response to valine, glutamate, and glutamine, repectively. Red bars represent an upregulation, green bars represent a downregulation, and grey bars refer to messages that are not considered to be significantly changed by our cutoff (three-fold).

When the preferred nitrogen sources, e.g., glutamate plus ammonia or glutamine, are not present in the growth medium, *B. subtilis* may activate PucR-regulon to utilize alternative nitrogen sources, such as purine, allantoic acid, allantoin, and uric acid. PucR is a transcriptional activator involved in the purine degradation pathway. PucR induces the expression of *yurHG*, *pucH*, *ywoE*, *pucJKLM*, *ureABC*, and *guaD* while it represses the expression of *pucR* and *pucABCDE*. The purine degradation in *B. subtilis* is subjected to a two-level positive control mechanism involving TnrA and PucR. The addition of glutamine, as the favoured nitrogen source, *pucR* gene and most genes of *pucR*-regulon were significantly repressed promptly (Fig. S3). The glutamine inactivated TnrA, and repress the the expression of *pucR*. The *pucABC* genes, encoding xanthine dehydrogenases as first step of hypoxanthine and xanthine degradation pathway, were obviously repressed immediately. Meanwhile guanine and xanthine permease genes *pbuX, pbuG, pbuO (ytiP)* controlled by PurR were induced at 5–120 min, and to mediate the uptake of hypoxanthine, guanine and xanthine. Consequently, the integration of the expression of these genes leads to enlargement of cellular nucleotide pools for rapid growth. Valine displayed no effect on the purine degradation pathway. Only *pbuX* was significantly induced. Fig. 8 depicted the effects of valine, glutamate, and glutamine on the salvage and metabolism of nucleotides.

#### Fatty-acid metabolism

In *B. subtilis*, two transcriptional global regulators, FadR (YsiA), a TetR family member, and FapR (YlpC), a DeoR family member, are involved in fatty acid degradation and biosynthesis respectively. FadR represses the expression of genes of fatty acid degradation [65], whereas FapR represses almost genes involved in all the processes in the biosynthesis of saturated fatty acids and phospholipids. 20 genes that are most correlated with Fap-regulon expression pattern (Fatty Acid Biosynthesis) were found across 116 microarray experiments by Hans Peter Fischer *et al.* [66], including *yjaX, yjaY, yhfB, gltT, fabD, accB, yhdO, ylpC, fabG, yxlF, panD, yjbW, panB, ylmB, plsX, yraK, azlD, ykoK, yobL*, and *yrhI*. Indeed, these genes were induced by valine, whereas were repressed by glutamate and glutamine (Fig. S4).

**Figure 9:**
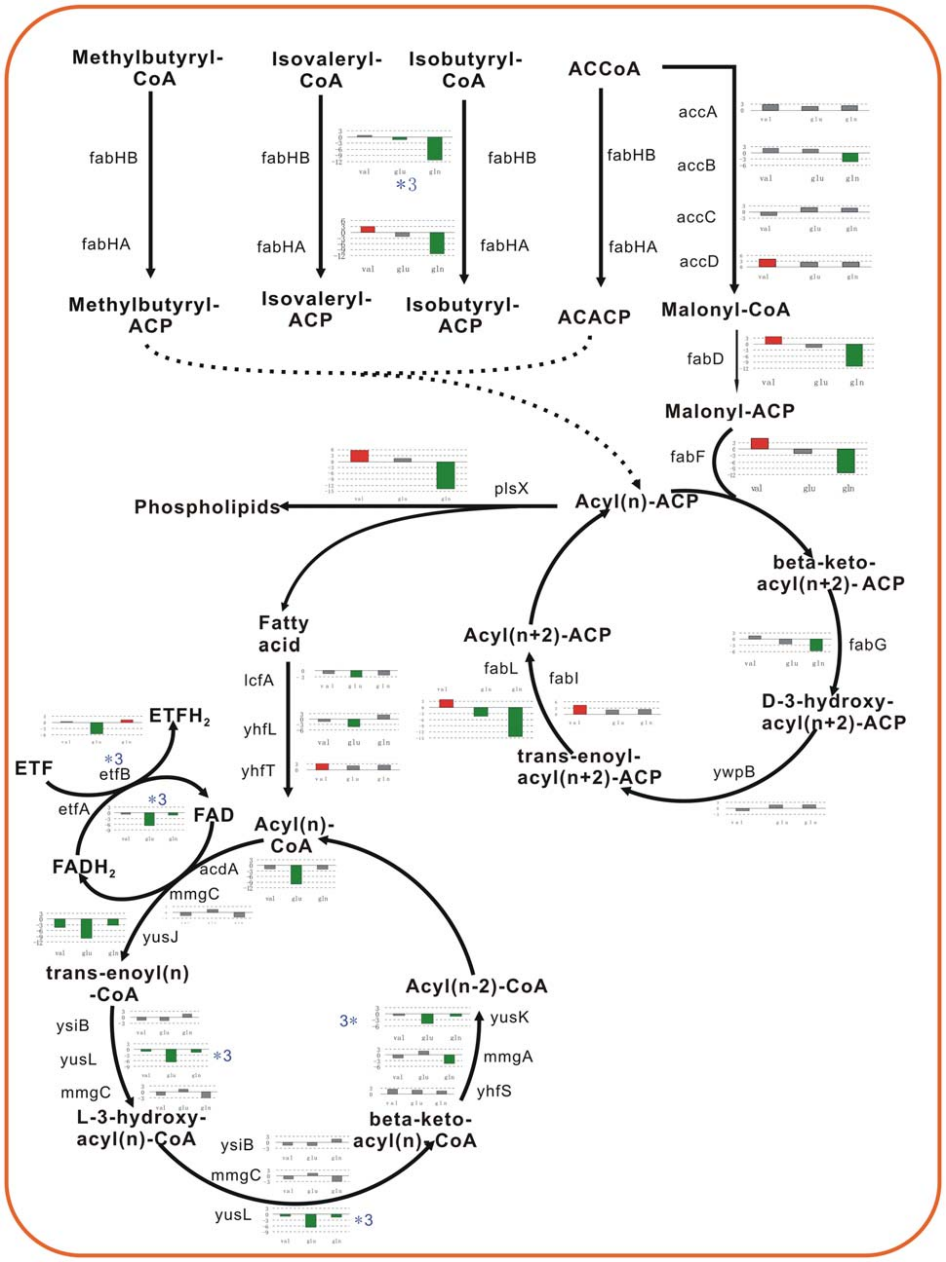
Effects of three amino acids on metabolism of fatty-acid. The expression of the genes involved in biosynthesis and degradation of fatty-acid is schematically presented. Three bars from left to right represent the fold changes of gene expression in response to valine, glutamate, and glutamine, repectively. Red bars represent an upregulation, green bars represent a downregulation, and grey bars refer to messages that are not considered to be significantly changed by our cutoff (three-fold).

A total of 25, 14, and 25 genes, involved in the metabolism of lipids were identified as significantly expressed at one or more time points in response to additions of Val, Glu, and Gln, respectively. Valine significantly induces *bkd* opreon immediately, involved in the utilization and degradation of isoleucine and valine for biosynthesis of branched chain fatty acid. As expected, T-profiler showed a clear induction of *bkd* operon genes to valine addition. The change on expression of the *bkd* operon was not observed after Glu and Gln addition. In addition, valine exhibits the moderate induction of FapR regulon, *uppS*, and *accD* genes for the biosynthesis of fatty acids and phospholipids, and repression of *yusJKL* operon (*fadN-A-E*) related to fatty acid β-oxidation. Glutamate exerts immediate and strong effect on the fatty acid β-oxidation. The operons *fadF* (*ywjF*)*-acdA-rpoE, yusJKL, lcfA-fadR-fadB-etfB-etfA*, *fadH-fadG* (*ykuF-ykuG*) are significantly downregulated after the addition of glutamate. Glutamine exhibits the moderate repression of *fapR* regulon genes for the biosynthesis of fatty acids and phospholipids, and *yusJKL*, *lcfA-fadR-fadB-etfB-etfA*, *fadH-fadG* operons related to fatty acid β-oxidation (Fig. S4). The availability of three amino acids did not displayed impact on expression of *mmgABC* operon. It is noteworthy that *ywiE* gene, encoding cardiolipin (CL) synthase, is downregulated by three amino acids, and *des* gene, encoding fatty acid desaturase, are obviously downregulated by glutamate and glutamine. Two genes are both involved in the membrane components. The adjustment of membrane components maybe change the biophysical state of the membranes, which plays a key role in the regulation of some transporters [67]. *B. subtilis* has three putative anionic phospholipid cardiolipin (CL) synthases (*ywnE, ywiE* and *ywjE*). The transcriptional microarray analysis of stress responses has indicated that the *ywiE* gene is induced during ethanol or NaCl stress, its promoter being sigma B-dependent, as for most of the genes responding to general stress effectors [68]. Three amino acids repress the expression of *ywiE* gene at 30–120 min with 10–27 folds, and reduce the synthesis of anionic phospholipid cardiolipin. As a result, the decrease of negatively charged lipids in the lipid bilayer of the cells would reduce the negative charge of the surface of the membrane. *B. subtilis* does not produce unsaturated fatty acids via the de novo biosynthetic pathway, but rather forms *iso*- and *anteiso*-branched chain fatty acids. However, when the bacteria are subject to an abrupt shift to a lower growth temperature, existing phospholipids are desaturated to increase the fluidity of the membrane bilayer. This adaptive reaction is carried out by Δ5-fatty acid desaturase (Δ5-Des), a membrane-bound acyl desaturase that introduces a double bond at the 5-position of the fatty acid chain on intact phospholipids [69]. The *des* gene is tightly regulated by a two-component regulatory system DesK-DesR (YocF-YocG) encoded by the *desK-desR* genes located in an operon adjacent to the *des* gene. Des desaturation increases the fluidity of the membrane leading to reduces DesK kinase activity and reduced *des* expression. Diego de Mendoza *et al* proposed one model of *des* transcriptional control by DesK-DesR [70]. DesK is a bifunctional enzyme with both kinase and phosphatase activities that could assume different signaling states in response to changes in membrane fluidity. DesR is activated by DesK in response to a decrease in membrane lipid fluidity, and to induce *des* gene expression. Glutamate and glutamine repress immediately the expression of *des* gene with 14–55 folds. The significant change of *desK-desR* genes expression is not observed after addition of glutamate and glutamine. It is unclear that whether the repression of *des* gene by glutamate and glutamine is mediated through DesK-DesR or the unknown regulation mechanism of *des* gene seems to exist. Fig. 9 depicted the effects of valine, glutamate, and glutamine on the fatty-acid metabolism.

#### Metabolism of coenzymes and prosthetic groups

Thiamine is an essential cofactor comprising a sulfur heterocycle. Its biosynthesis needs two separated pathways. The first synthesizes the thiazole group (5-methyl-4-(-hydroxyethyl) thiazole), and the second a pyrimidine (4-amino-5-hydroxymethylpyrimidine pyrophosphate). These two molecules are combined to yield thiamine pyrophosphate. The pyrimidine moiety is derived from 5-aminoimidazole ribotide. Thiazole is derived from tyrosine, cysteine, and 1-oxy-D-xylulose-5-phosphate. The pathways involve several operons and genes such as *tenAI-goxB-thiSGF-yjbV (thiD)*, *ywbI-thiME*, *thiC, thiL*, *pdxK* (*ywdB*), *yuaJ* and *ykoFEDC (thiUVWX)*. Valine inactivates immediately the biosynthesis and transport of thiamine by downregulating *tenAI-goxB-thiSGF-yjbV* operon. Glutamate inactivates strongly the biosynthesis and transport of thiamine at 30–120 min by downregulating *tenAI-goxB-thiSGF-yjbV* operon (ca. 100-fold), *ykoFEDC* operon (ca. 10-fold), and *thiC* (ca. 9-fold). Glutamine displays the reverse effect on the biosynthesis of thiamine: moderately induce the *ykoFEDC* operon (ca. 3-fold) and *thiC* (ca. 4-fold). Recently Jenkins *et al*. [71] have identified a new pathway involved in the salvage of base-degraded forms of thiamin. Thiamin hydrolysis products such as N-formyl-4-amino-5-aminomethyl-2-methylpyrimidine (formylaminopyrimidine) are transported into the cell using the Thiamine transport system, Formyl aminopyrimidine can then be converted to aminopyrimidine by the *ylmB*-encoded amidohydrolase that in turn is hydrolyzed to hydroxypyrimidine by TenA (thiaminase II). Hydroxypyrimidine is then used as a building block for the biosynthesis of thiamine phosphates and in particular the cofactor thiamine diphosphate. We find that *ylmB* gene is downregulated (ca. 5-fold) at 30–120 min after glutamate addition. Valine also activated the biosynthesis of biotin (*bio* operon) and linear heptaprenyl unit (heptaprenyl−diphosphate synthase genes *hepS* and *hepT*). *B. subtilis* was reported to produce the catecholic siderophore itoic acid (2,3-dihydroxybenzoate (DHB)-glycine) encoded by the *dhb* operon in response to iron deprivation (Fur-regulon). In our case, *dhb* operon is induced by valine, whereas repressed by glutamate.

### Conclusion

In the current study, we exploit the accurate control of chemostat *B. subtilis* cultures to generate reproducible perturbation experiments with amino acids. Although the comprehensive transcriptional profiling of *B. subtilis* in response to amino acid mixtures availability has been studied [15], to our knowledge, this is the first comparative investigation of the effects of different mono amino acid on global gene expression over time. In addition, this is the first report to explore the relationship between the actions of valine, glutamate, and glutamine using a comprehensive expression profiling approach. We found that the addition of different amino acids resulted in a distinctive massive restructuring of the transcriptional output. The data about dynamics of transcript levels in response to each amino acid revealed overlap and divergence between the actions of these three amino acids. A total of 673, 835, and 1135 genes (approximately 16.4%, 20.3%, and 27.7% of all *B. subtilis* genes) were identified as significantly expressed at one or more time points in response to pulses of valine, glutamate, and glutamine, respectively, including genes, involved in cell wall, cellular import, metabolism of amino-acids, fatty acid, and nucleotides, transcriptional regulation, flagellar motility, chemotaxis, phage proteins, sporulation, and many genes of unknown function (40–62%). 213 genes were induced by both Glu and Gln, and 93 genes were upregulated by both Val and Gln. A group of 44 genes was upregulated by treatment with all three amino acids. A total of 219 genes were repressed by both Glu and Gln, and 214 genes were repressed by both Val and Gln. Only 118 genes were down-regulated by both Val and Glu. A total of 101 genes were mutually repressed by three amino acids. T-profiler analysis revealed that SigB-, SigW, and SigD-controlled genes had similar temporal expression pattern after Glu and Gln addition, the expression of genes dependent on SigK and SigE were significantly repressed by Val and Gln. The amino acids responsive genes with known-function were classified based on their established or putative functions according Subtilist database. This classification of genes is important for understanding the functional divergence and modes of action of amino acids on gene expression, and will be useful for obtaining a system view of the regulation effect of amino acids to provide insight into the activities of amino acids as effectors. The results revealed the different and sequential cellular processes in response to a sudden addition of various amino acids. Approximately 215, 140, and 113 genes, as rapidly responding genes, were found to change threefold during the first 5 min after treatment with valine, glutamate, and glutamine, respectively. The number of the rapidly valine-regulated genes was about twice than the rapidly glutamate/glutamine-regulated genes. Valine immediately and significantly regulated the expression of genes, involved in transports of nutrients, metabolism of amino acid, and ribosome biogenesis at 5 min, whereas most of these genes were regulated at 30 min after addition of glutamate/glutamine. This suggested that valine exerted the more direct effects on gene expression for the rapid growth. Glutamate and glutamine exhibited delayed regulation of the expression of these genes. We have also analyzed the effect of exogenous amino acids on gene expression of the major metabolic pathways in cell to better understand the role of amino acid in metabolic network. Our analysis demonstrated that the metabolism of *B. subtilis* was reprogrammed following amino acid pulses. The data revealed that three amino acids all resulted in induction of biosynthesis of purines and pyrimidines, sulfur assimilation, and biosynthesis of cysteine and methionine, whereas displayed different effects on metabolism of carbohydrate and nitrogen, biosynthesis and β-oxidation of fatty acid, metabolism of coenzymes and prosthetic groups. Our data comfirmed that amino acids significantly affected expression of sporulation, competence, chemotaxis, and motility genes, whereas three amino acids had different effects on expression of these genes.

Our study provide new insight into the mechanism of amino acid function as effectors to transcriptional regulation, and to better elucidate the specific actions of each amino acid. In view of the diversity and complexity of signaling network underlying gene expression responses to valine, glutamate, and glutamine, many fundamental mechanisms remained to be resolved. In future investigations, it will be interesting to uncover the regulatory interplay between transcriptional network, metabolic network, and the metabolites that serve as effectors for bridging the the gap between the metabolic and regulatory networks in *B. subtilis*. These studies will be essential to meet the longstanding challenge of systems biology: to reconstruct integrated model of transcriptional and metabolic networks including regulation mediated by metabolites, and to come to an integral understanding of responses of living cells to environment.

## Materials and Methods

### Strain, media and growth condition

In this study, *Bacillus subtilis* 168 was used. Bacteria was inoculated in LB medium (10 g tryptone, 5 g yeast extract, and 10 g NaCl per liter of distilled H_2_O), grown for 16 h at 37°C and 200 rpm. The seed culture was used to inoculate in a an aerobic chemostat culture in a 5-L fermentor with a working volume of 2 L on the adapted medium (2 g (NH_4_)_2_SO_4_, 7g K_2_HPO_4_, 3 g KH_2_PO_4_, 1 g sodium citrate, 0.2 g MgSO_4_·7H_2_O, 5 g glucose per liter of distilled H_2_O). The culture was allowed to grow in batch mode for 7–8 h before the feed pump and effluent pump were turned on to start the chemostat. The concentration of glucose was increased to 20 g per liter of distilled H_2_O in feed medium. Chemostat experiments were performed under aerobic conditions at a dilution rate of 0.1 h^−1^ based on growth rate of the strain. The pH was measured using a glass electrode and controlled at 7.0 using ammonia water. Other fermentation parameters are a temperature controlled at 37°C, an inlet airflow of 0.6 L/min, stirrer speed of 500 rpm and dissolved oxygen higher than 30%. Optical density (OD_600_), remaining sugar concentration and colony morphous were measured every one or two hours during fermentation, the continuous culture was considered as steady state if these fermentation parameters were stable.

At the age of 70 h, the steady-state chemostat culture was perturbed by the addition of amino acid (valine, glutamate, or glutamine) to the fermentor so that amino acid concentration was suddenly increased to about 1.8 g/l, 3.3 g/l, or 4.6 g/l (15.4 mM, 22.4 mM, or 31.5 mM). The amino acid was rapidly injected by a pneumatic system (<1 s). The samples were harvested prior to the amino acid pulse (steady-state samples, t = 0, as reference samples) and at different time points (5 min, 30 min, 2 h, 8 h, 24 h) after the perturbation. Samples drawn for RNA isolation were immediately quenched in liquid nitrogen, and stored at −80°C. The whole procedure took no longer than 50 s. Two independently cultured replicates were performed for the valine pulse experiment. The results for glutamate and glutamine were derived from a single culture.

### Microarray construction

The *B. subtilis* DNA microarrays (BSU v1.0) were customized using Agilent eArray 5.0 program according to the manufacturer's recommendations (https://earray.chem.agilent.com/earray/). Each customized microarray (8 X15K) contained spots in triplicate with 4,106 gene-specific 60-mer oligonucleotides representing the 4,106 protein-coding genes in *B. subtilis* (as reported for the *B. subtilis* genome at http://genolist.pasteur.fr/SubtiList/).

### RNA isolation, labeling, hybridization, and scanning

30 mL of culture broths from chemostat culture were sampled, rapidly filtered within seconds, and quenched in liquid nitrogen, subsequently stored at −80°C until further use. Total RNAs were isolated from culture samples by using the Trizol reagent (Gibco BRL, Cleveland, OH) according to the standard protocols. The RNAs were subsequently purified by QIAGEN RNeasy Mini Kit. The quality and quantity were determined by nanodrop UV spectroscopy (Ocean Optics) and analysis on a RNA 6000 Nano LabChip (Agilent Technologies) using a 2100 bioanalyzer (Agilent Technologies).

Agilent Quick Amp Kit (Agilent Technologies) was used to synthesize cDNA from total RNA samples of 2 µg and subsequently produce the amino allyl modified cRNA according to the supplier's manual, except using amino allyl-dUTP (Ambion). The amplified cRNAs were purified using Qiagen's RNeasy mini spin columns, and quantitated by Nanodrop ND-1000. In the next step, the amino allyl labeled cRNA of 4 µg is coupled with the monoreactive succinimide ester derivative of a Cy dye (GE healthcare, PA13105 or PA15100). The fluorescently labeled cRNA is purified with a RNeasy mini spin column, to remove any unreacted dye. The resulting fluorescently labeled cRNA is fragmentated in fragmentation buffer (Agilent). The prepared-as cRNA of 55 µl was mixed in 55 µl GEx Hybridization Buffer HI-RPM (Agilent). Hybridization was performed in an Agilent Microarray Hybridization Chamber (G2534A) for 16 h at 65°C at rotation of 10 rpm. After the hybridization, the slides were washed in Gene Expression Wash Buffer (Agilent). Microarrays were scanned using an Agilent G2565BA scanner.

### Microarray data extraction, processing, and analysis

Feature extraction and image analysis software (Feature Extraction Software; Agilent Technologies) was used to locate and delineate every spot in the array, to integrate each spot's intensity, and to normalize data using the Lowess method [72]. After masking data points with flag value of non-zero or a signal-to-noise ratio smaller than 2.6, the remaining data were log transformed and averaged for each gene and each time-point. All microarray data reported in the manuscript is described in accordance with MIAME guidelines. A threefold change and a less than pValueLogRatio (pValueLogRatio<0.05) were used as thresholds for selection of amino-acid-regulated genes. Clustering analyses were performed using Microarray Expression Viewer software MeV 4.0 (MEV-TIGR; http://www.tm4.org/mev.html). All amino-acid-regulated genes were distributed over MIPS functional categories as a function of time (s) (http://mips.gsf.de/projects/funcat) with a threshold *p*-value of 0.01 with Bonferroni correction.

To identify different gene expressional reponses to different amino acid pulses, the probe-level log ratios in each two pulse experiments were tested for difference using the paired t-test, and DE probes were determined with thresholds of *p*-values adjusted by the BH method [73] (adjusted *p*-value< = 0.2 for Val vs. Glu and Val vs. Gln; nominal *p*-value <0.05 for Glu vs. Gln). Combining these between-experiment DE probes led to 2508 DE probes, or 922 DEGs. A hierarchical clustering of the 922 DEGs was also performed using MeV 4.0.

To assess the contribution of the expression of genes from specific gene classes to the total gene expression of all 4,003–4,091 genes, all genes were analyzed with T-profiler [74]. T-profiler was adapted for the use of *B. subtilis* transcriptome data by implementing predefined gene groups from the following sources: the Database of Transcriptional Regulation in *Bacillus subtilis* (DBTBS) (release May 2006), the Kyoto Encyclopedia of Genes and Genomes database (KEGG) (release May 2006), the SubtiList database, and the stringently controlled genes by Alex Ter Beek *et al.* [28]. All microarray data were online performed for T-profiler analysis (http://www.science.uva.nl/~boorsma/t-profiler-bacillusnew/). Gene groups regulated positively or negatively by a specific transcription factor were named accordingly. For transcription factors acting both as an activator and as a repressor, separate gene groups were made.

## Supporting Information

Dataset S1 Clusters and List of Genes That Significantly Changed upon AA Additions. Each worksheet contains amino acid-responsive gene clusters (three-fold changes). Columns in each worksheet indicate the following (from left to right): Heatmaps of clusters; Gene name; Log2 ratios (expression of samples of T = 5 min, 30 min, 2 h, 8 h, or 24 h versus reference sample of T = 0); Sig: sigma factor; Reg: transcriptional regulator; Description.
(3.26 MB XLS)

Figure S1 Effects of Three Amino Acids on Biosynthesis of Amino Acids. The effects of valine (A), glutamate (B), and glutamine (C) on the metabolism of twenty amino acids are shown. Red arrows: activation of pathway; Green symbal T: repression of pathway.
(0.93 MB TIF)

Figure S2 The expression profiles of genes involved in metabolism of sulfur. Expression pattern of genes of YrzC-regulon after treatments with Val (A), Glu (B), and Gln (C), other genes related to sulfur metabolism after treatments with Val (D), Glu (E), and Gln (F), yjcIJ (metIC), ykrV (mtnV), ykrZ (mtnZ, mtnD).
(1.65 MB TIF)

Figure S3 The expression profiles of genes related to nucleotide metabolism. Expression pattern of PyrR operon, PucR-regulon, and PurR-regulon in response to Val (ADG), Glu (BEH), and Gln (CFI).
(2.72 MB TIF)

Figure S4 The expression profiles of genes involved in fatty acid metabolism. Expression pattern of the genes related to fatty acid biosynthesis in response to val(A), Glu (B), and Gln (C). Expression pattern of the genes related to fatty acid degradation in response to val(D), Glu (E), and Gln (F).
(1.33 MB TIF)

Table S1 Between-experiment Differentially Expressed Genes. Columns indicate the following (from left to right): Heatmaps of clusters; Gene No; Gene name; Sig: sigma factor; Reg: transcriptional regulator; Description; and Functional category (Subtilist Database).
(0.62 MB XLS)

Table S2 Rapidly AA-responsive Genes (at 5 min). All rapidly AA-responsive genes and change folds were listed by the induction, repression, and functional classifications (Subtilist Database).
(0.07 MB XLS)

Table S3 T-values of Gene Groups using Subtilist-pathway Model. The expression values of all genes after amino acid treatments (Dataset S1) were performed with Subtilist-pathway Model for T-profiler analysis in-line (http://www.science.uva.nl/boorsma/t-profiler-bacillusnew/). T-value, E-value (>0.05), Mean, and ORFs are listed.
(0.03 MB XLS)

Table S4 T-values of Gene Groups using Transcript Regulators Model. The expression values of all genes after amino acid treatments (Dataset S1) were performed with transcript regulators model for T-profiler analysis in-line (http://www.science.uva.nl/boorsma/t-profiler-bacillusnew/). T-value, E-value (>0.05), Mean, and ORFs are listed.
(0.04 MB XLS)

Table S5 T-values of Gene Groups using KEGG-pathwayModel. The expression values of all genes after amino acid treatments (Dataset S1) were performed with KEGG-pathway model for T-profiler analysis in-line (http://www.science.uva.nl/boorsma/t-profiler-bacillusnew/). T-value, E-value (>0.05), Mean, and ORFs are listed.
(0.04 MB XLS)

Text S1 Replicate and Reproducibility. Reproducibility of replicate experiments was discussed.
(0.14 MB DOC)

Text S2 Expression Analysis of Some Genes in Response to Val, Glu, and Gln. The genes, involved in sporulation/germination, motility, cell-wall, cysteine/methionine metabolism, phosphate metabolism, and transcriptional factors, were discussed.
(3.92 MB DOC)
