# Supplementary figures and images for: Time-Resolved Transcriptome Analysis of Bacillus subtilis Responding to Valine, Glutamate, and Glutamine
Source: PLoS One. 2009 Sep 18;4(9):e7073. doi: 10.1371/journal.pone.0007073 (PMC2743287; doi:10.1371/journal.pone.0007073)

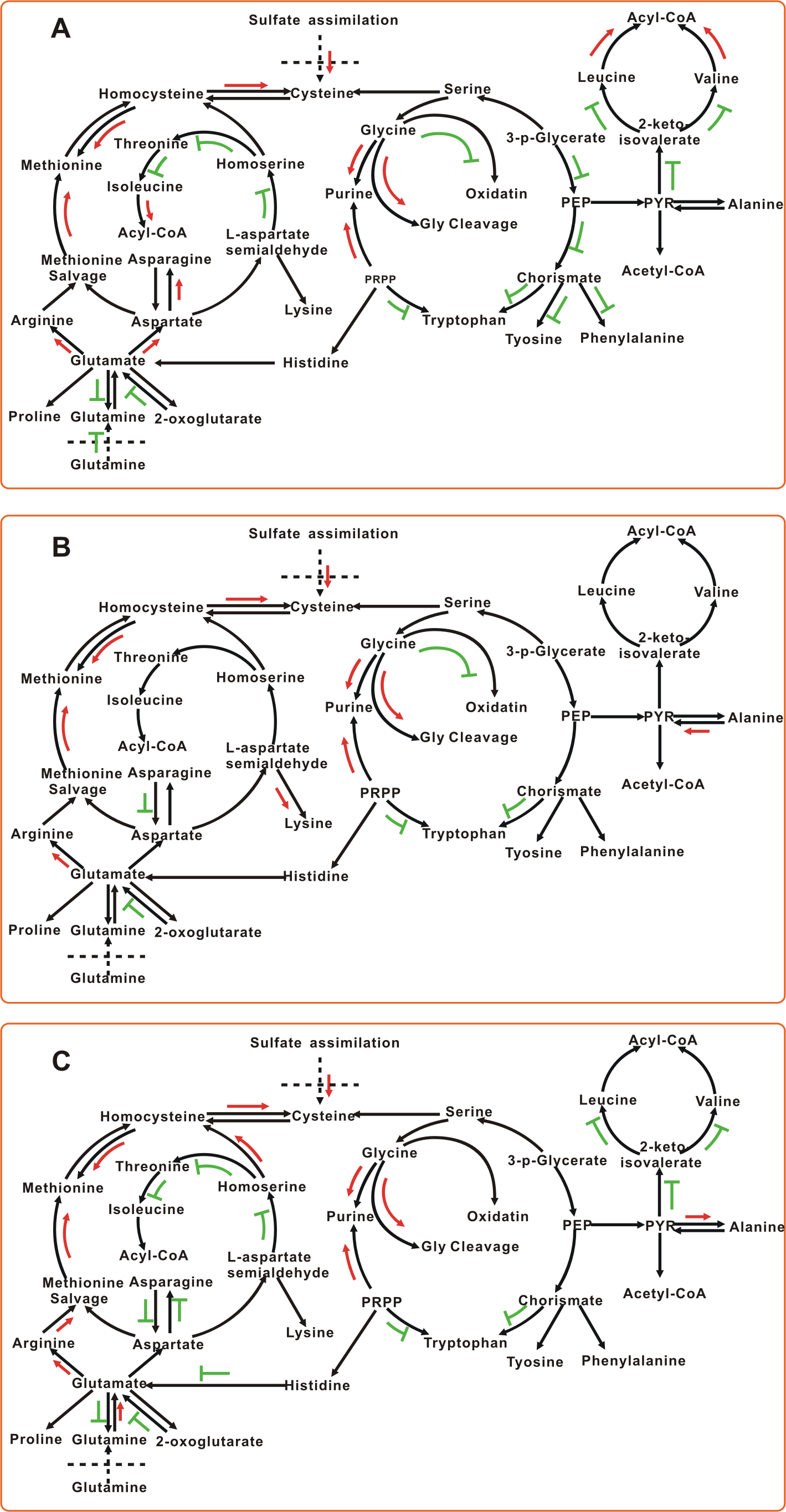

Supplement: Figure S1 — Effects of Three Amino Acids on Biosynthesis of Amino Acids. The effects of valine (A), glutamate (B), and glutamine (C) on the metabolism of twenty amino acids are shown. Red arrows: activation of pathway; Green symbal T: repression of pathway. (0.93 MB TIF) [file pone.0007073.s002.tif]

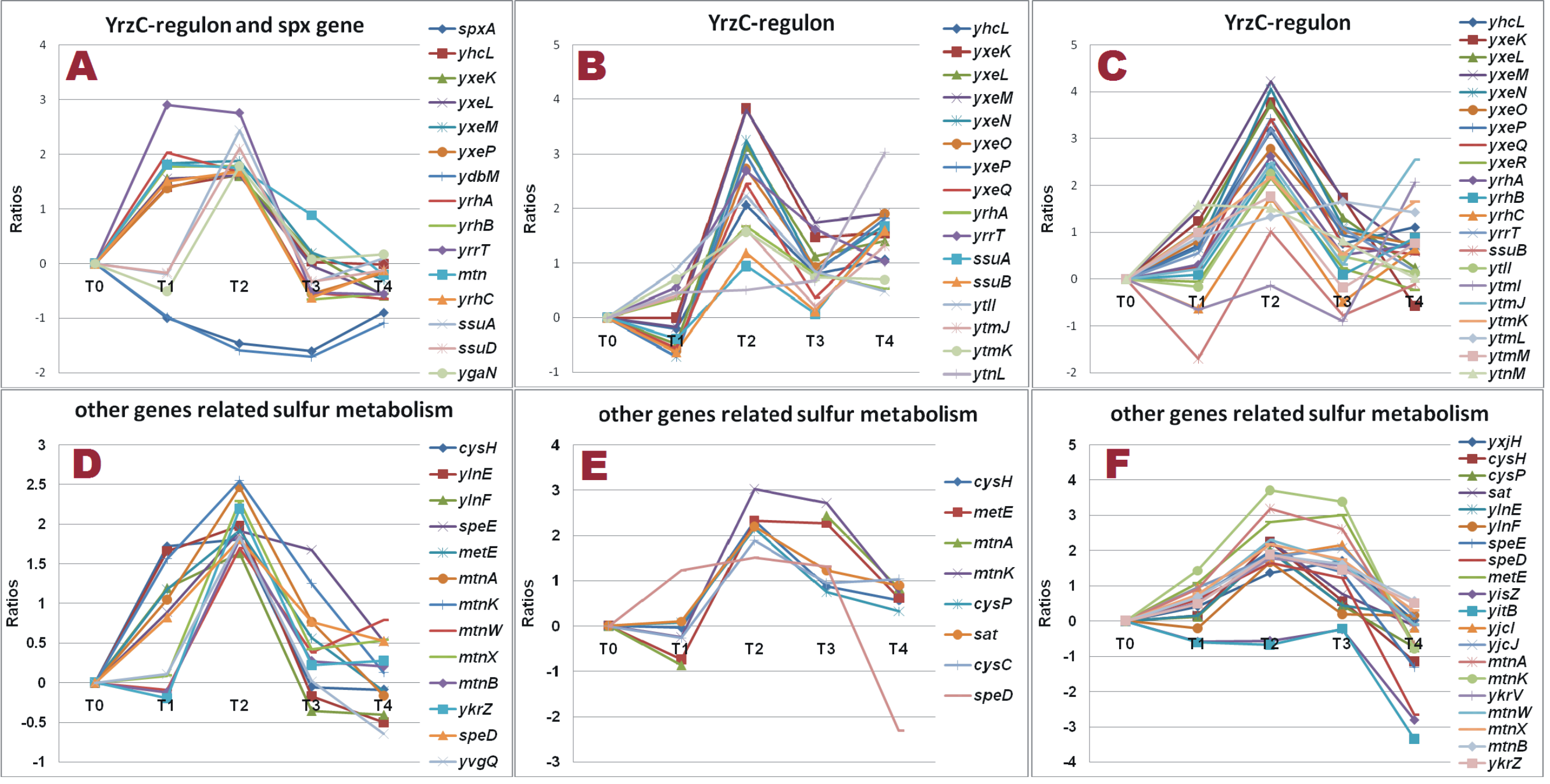

Supplement: Figure S2 — The expression profiles of genes involved in metabolism of sulfur. Expression pattern of genes of YrzC-regulon after treatments with Val (A), Glu (B), and Gln (C), other genes related to sulfur metabolism after treatments with Val (D), Glu (E), and Gln (F), yjcIJ (metIC), ykrV (mtnV), ykrZ (mtnZ, mtnD). (1.65 MB TIF) [file pone.0007073.s003.tif]

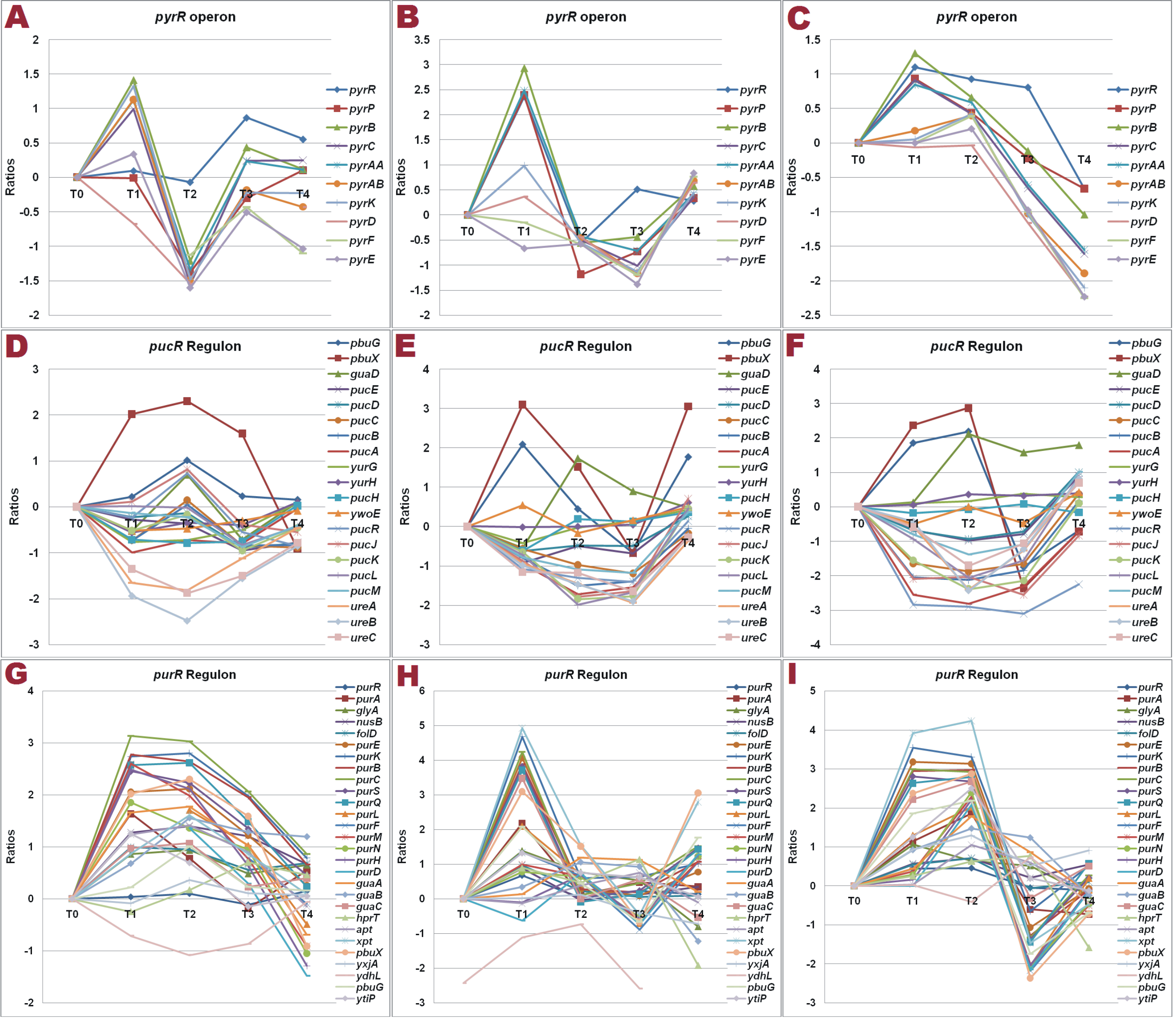

Supplement: Figure S3 — The expression profiles of genes related to nucleotide metabolism. Expression pattern of PyrR operon, PucR-regulon, and PurR-regulon in response to Val (ADG), Glu (BEH), and Gln (CFI). (2.72 MB TIF) [file pone.0007073.s004.tif]

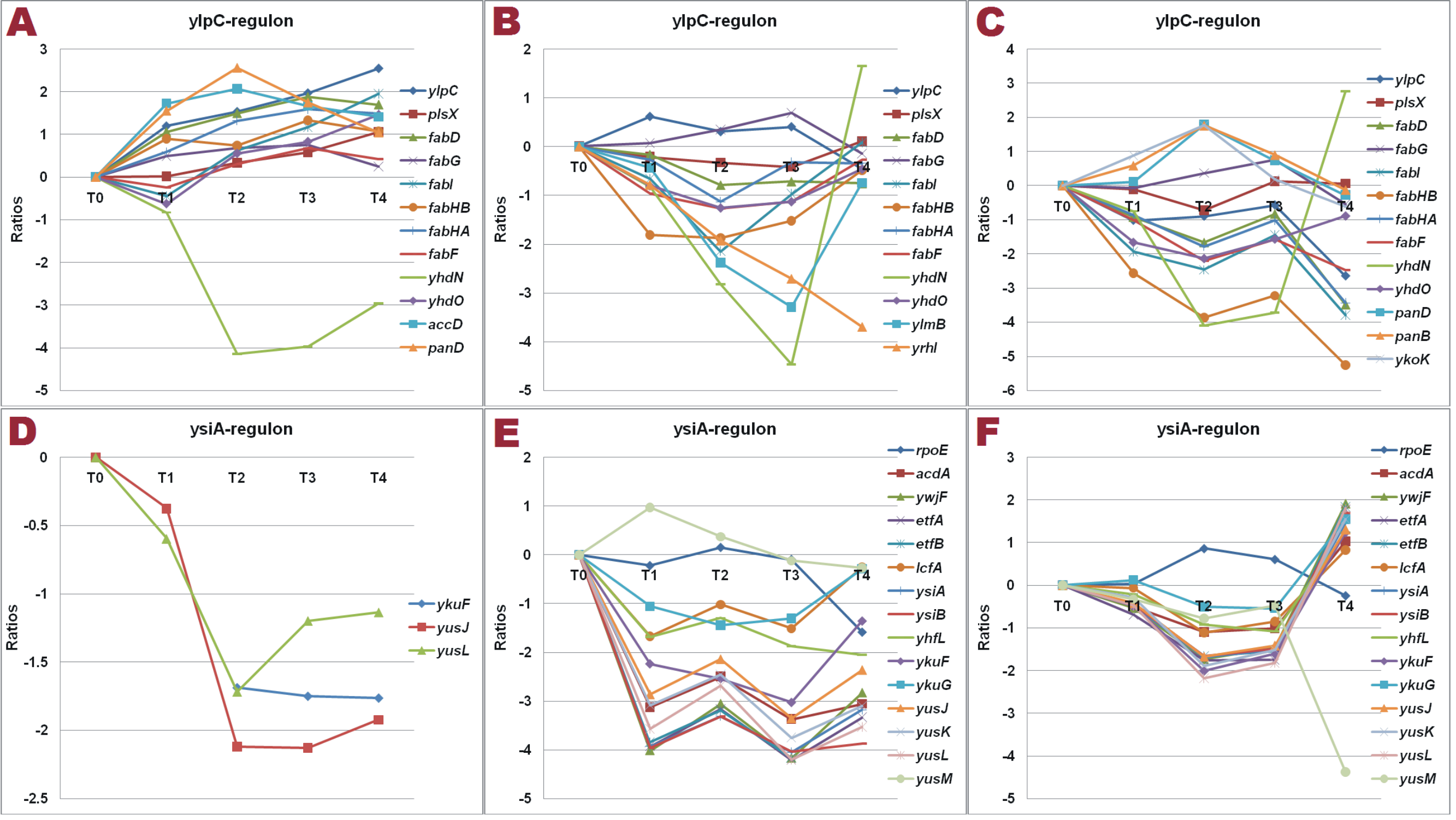

Supplement: Figure S4 — The expression profiles of genes involved in fatty acid metabolism. Expression pattern of the genes related to fatty acid biosynthesis in response to val(A), Glu (B), and Gln (C). Expression pattern of the genes related to fatty acid degradation in response to val(D), Glu (E), and Gln (F). (1.33 MB TIF) [file pone.0007073.s005.tif]
