# Supplementary material for: Time-Resolved Transcriptome Analysis of Bacillus subtilis Responding to Valine, Glutamate, and Glutamine
Source: PLoS One. 2009 Sep 18;4(9):e7073. doi: 10.1371/journal.pone.0007073 (PMC2743287; doi:10.1371/journal.pone.0007073)
Supplement: Text S1 — Replicate and Reproducibility. Reproducibility of replicate experiments was discussed. (0.14 MB DOC) [file pone.0007073.s011.doc]

## Supporting Information:

## Time-resolved Transcriptome Analysis of *Bacillus subtilis* Responding to Valine, Glutamate, and Glutamine

**Test S1 Replicate and reproducibility**

Two independent cultures were pulsed with valine. The raw data were read into R using the Limma function read.maimages, which recognized the mean intensity of each probe and the median of the corresponding background. To remove the intensity-level-dependent bias of log ratios, all probes except the control spots underwent the loess normalization. As the red channels (Cy5) all reflect gene expressions in the reference sample, which should have no between-array difference, we applied the ‘Rquantile’ normalization method from the limma package to ensure that the red (second) channel has the same empirical distribution across arrays. The log-scaled intensities in the green channels (i.e., expression values of the samples of interest), and the M (log2 ratio) values were output for subsequent analyses.

Log intensities in either channel demonstrated a high correlatioin between the two series of replicated measurements (above 0.9, p<1e-16), and correlation was also significant if examining the replicated log ratios. Additionally, we investigated the relationship between CVs (coefficients of variation) and mean values. This was done for log2R, log2 G, and M, respectively. It is seen that CVs of log intensities in either channel were nearly all below 20%, but CVs of Ms were much higher. However, the CV of M goes down with the absolute value of M. It means that higher M is associated with lower variance.

These two independent pulses were highly reproducible and the median coefficient of variation (CV, S.D. divided by the mean) for the signals of red and green channels at replicate time point of 5 min was below 6.1%.

Figure S1


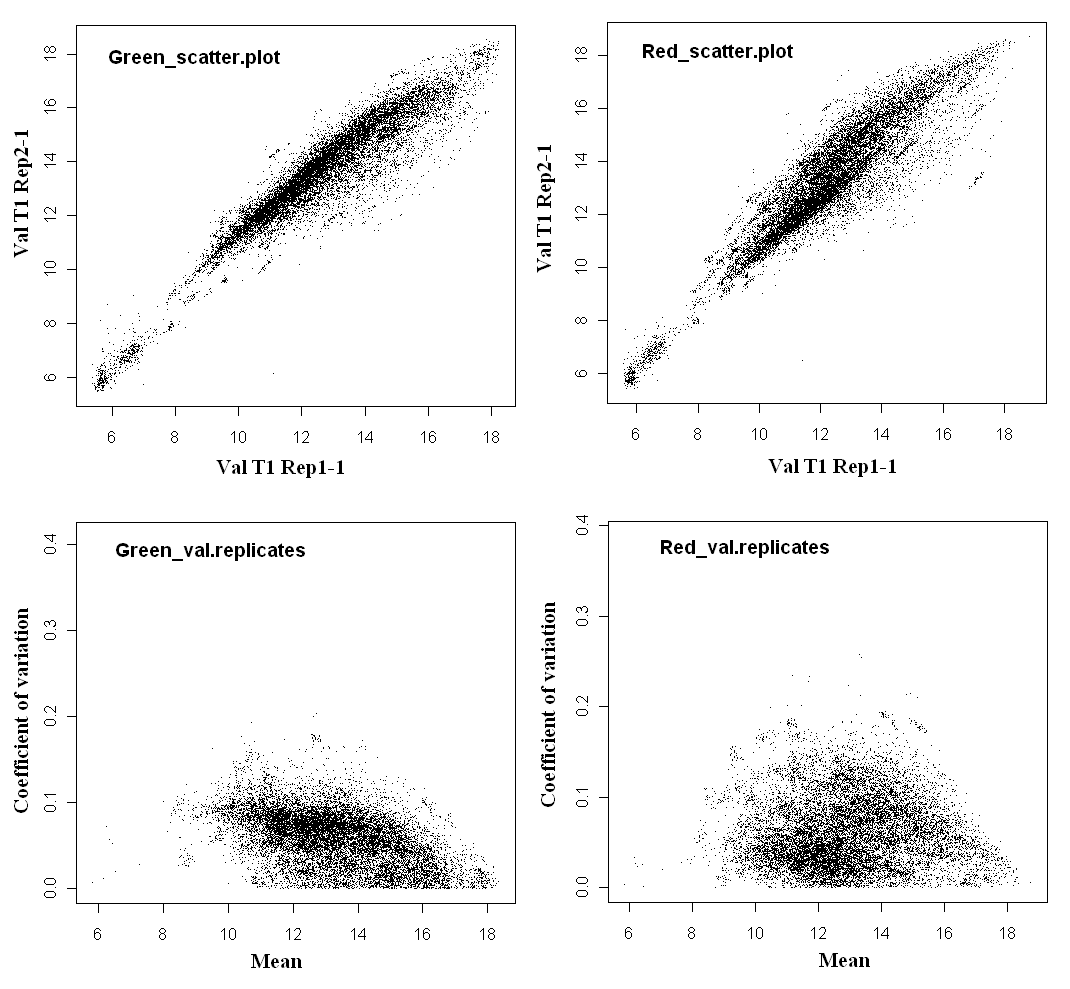


Table S1 The correlation values and median CV between biological replicates

|  | Correlation value | p-value | median(cv) % |
| --- | --- | --- | --- |
| Log2 R | 0.92 | <2.2e-16 | 6.12 |
| Log2 G | 0.94 | <2.2e-16 | 5.40 |
| M ( > threefold) | 0.82 | <2.2e-16 | 15.8 |
