# Supplementary material for: Time-Resolved Transcriptome Analysis of Bacillus subtilis Responding to Valine, Glutamate, and Glutamine
Source: PLoS One. 2009 Sep 18;4(9):e7073. doi: 10.1371/journal.pone.0007073 (PMC2743287; doi:10.1371/journal.pone.0007073)
Supplement: Text S2 — Expression Analysis of Some Genes in Response to Val, Glu, and Gln. The genes, involved in sporulation/germination, motility, cell-wall, cysteine/methionine metabolism, phosphate metabolism, and transcriptional factors, were discussed. (3.92 MB DOC) [file pone.0007073.s012.doc]

## Supporting Information:

## Time-resolved Transcriptome Analysis of *Bacillus subtilis* Responding to Valine, Glutamate, and Glutamine

**Test S2 Expression of some genes in response to val, glu, and gln**

**1. Expression of sporulation and germination genes**

There are 153 genes related to sporulation in DBTBS database, 37, 17, and 83 genes of among them showed clear repession after Val, Glu, and Gln treatments, respectively (Fig. S1 and Table S1).

**
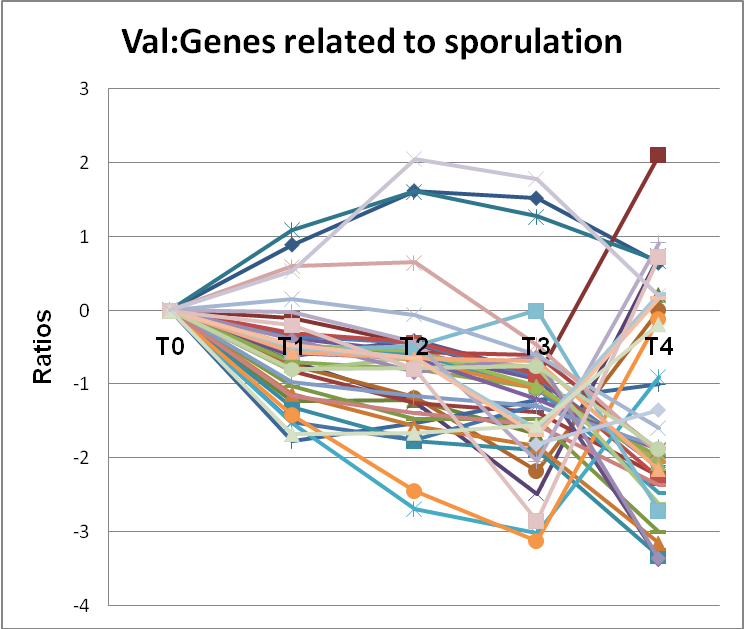
** **
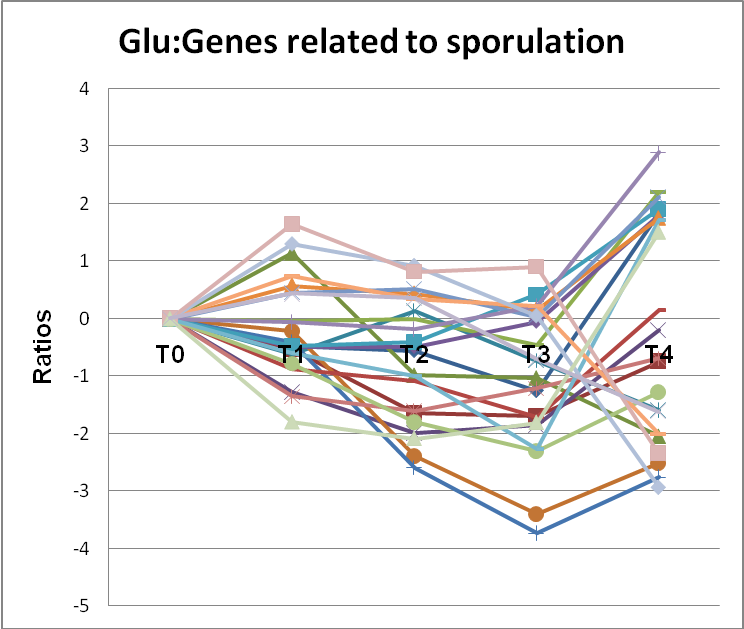
** **
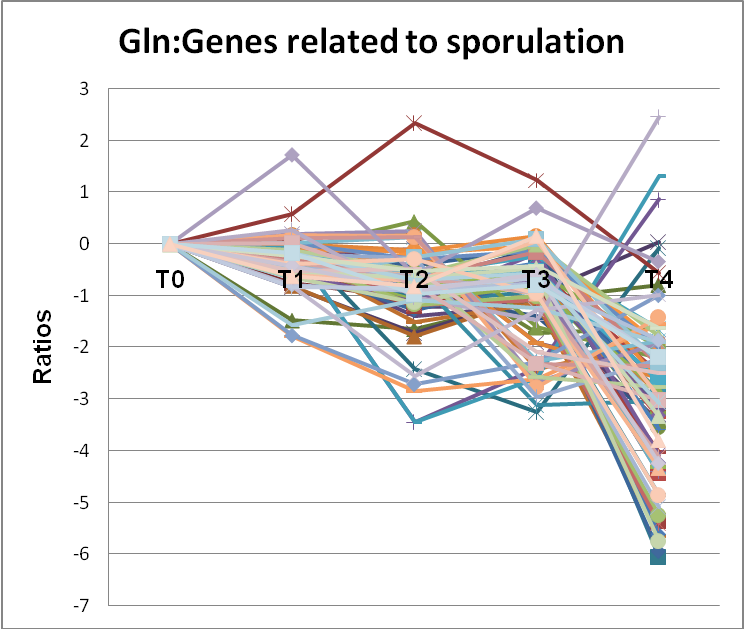
**

**Figure S1.** Expression pattern of genes ralated to sporulation after treatments with Val (A), Glu (B), and Gln (C)

**Table S1. AA-responsive genes related to Sporulation**

| Gene Name | Ratios | | | | Sig | Reg |
| --- | --- | --- | --- | --- | --- | --- |
|  | T1 | T2 | T3 | T4 |  |  |
|  |  |  |  |  |  |  |
| **Val-responsive genes** | | | | | | |
| spoIIIJ | 0.88 | 1.61 | 1.51 | 0.64 | SigA |  |
| spoIIE | -0.11 | -0.46 | -0.92 | 2.10 | SigA | Spo0A |
| spoVR | -1.23 | -1.21 | -1.67 | 0.21 |  |  |
| spoIVA | -0.68 | -1.24 | -2.48 | 0.74 | SigE | SpoIIID |
| spo0B | 1.09 | 1.61 | 1.27 | 0.66 | SigA |  |
| spoVID | -0.74 | -1.17 | -2.17 | 0.01 | SigE |  |
| cotA | -1.76 | -1.52 | -1.22 | -0.99 | SigK | GerE/SpoIIID |
| cotD | -0.82 | -1.26 | -1.37 | -2.24 | SigK | GerE/SpoIIID/Spo0A |
| cotV | -1.03 | -1.47 | -1.47 | -3.00 | SigK | GerE/SpoIIID |
| cotW | -0.62 | -0.40 | -0.85 | -3.37 | SigK | GerE/SpoIIID |
| cotY | -1.32 | -1.77 | -1.89 | -3.32 | SigK | GerE/YlbO |
| cotZ | -1.13 | -1.57 | -1.84 | -3.15 | SigK | GerE/YlbO |
| cotX | -1.51 | -1.75 | -1.28 |  | SigK | GerE/SpoIIID |
| spsA | -0.50 | -0.57 | -0.60 | -1.90 | SigK |  |
| spsB | -0.70 | -0.79 | -1.05 | -1.98 | SigK |  |
| spsD | -0.38 | -0.56 | -0.94 | -2.10 | SigK |  |
| spsE | -0.48 | -0.60 | -1.05 | -2.47 | SigK |  |
| spsF | -0.52 | -0.67 | -1.06 | -2.05 | SigK |  |
| spsG | -0.36 | -0.44 | -0.91 | -2.12 | SigK |  |
| spsI | -0.31 | -0.44 | -0.87 | -2.27 | SigK |  |
| spsJ | -0.48 | -0.56 | -1.02 | -2.06 | SigK |  |
| spsL | -0.58 | -0.65 | -1.20 | -1.89 | SigK |  |
| rapA | -1.52 | -2.69 | -3.01 | -0.90 | SigA | Spo0A/ComA |
| phrA | -1.41 | -2.44 | -3.12 | -0.11 | SigA | Spo0A/ComA |
| spoVFA | -0.97 | -1.16 | -1.30 | -1.85 | SigA/SigK |  |
| spoVFB | -1.17 | -1.39 | -1.55 | -2.37 | SigA/SigK |  |
| cotG | -0.82 | -0.63 | -0.78 | -2.62 | SigK | GerE/YlbO |
| cgeA | -0.39 | -0.84 | -0.63 | -3.36 | SigK | GerE/YlbO |
| cgeB | -0.54 | -0.49 | -0.01 | -2.71 | SigK | GerE/YlbO |
| cgeC | -0.56 | -0.65 | -0.68 | -2.15 | SigK | GerE |
| cgeE | 0.15 | -0.06 | -0.61 | -1.59 | SigK | GerE |
| kipA | 0.60 | 0.65 | -0.47 | -1.94 | SigK | KipR/TnrA |
| cotS | -0.80 | -0.78 | -0.75 | -1.88 | SigK |  |
| spoIIIAH | -0.03 | -0.43 | -2.04 | 0.91 | SigE | YlbO/SpoIIID |
| cotJB | -0.50 | -0.64 | -1.61 | 0.22 | SigE | SpoIIID |
| cotJC | -0.46 | -0.64 | -1.68 | 0.17 | SigE | SpoIIID |
| cotM |  |  | -1.81 | -1.34 | SigK | GerE |
| spoIIQ | -0.20 | -0.80 | -2.85 | 0.72 | SigF |  |
| usd | -1.68 | -1.66 | -1.55 | -0.18 | SigE |  |
| spo0M | 0.53 | 2.05 | 1.78 | 0.20 | SigH |  |
|  |  |  |  |  |  |  |
| **Glu-responsive genes** | | | | | | |
| yaaH | -0.48 | -0.56 | -1.25 | 1.84 | SigE |  |
| spoVG | -0.53 | -1.64 | -1.69 | -0.74 | SigH | AbrB |
| spoIIGA | 1.14 | -0.98 | -1.03 | -2.05 | SigA/sigF/SigG | Spo0A/RsfA/SpoVT |
| cotA | -1.27 | -1.99 | -1.84 | -0.19 | SigK | GerE/SpoIIID |
| rsfA | -0.62 | 0.14 | -0.73 | -1.59 | SigF/SigG | RsfA |
| rapA | -0.21 | -2.39 | -3.40 | -2.51 | SigA | ComA/Spo0A |
| phrA | -0.40 | -2.59 | -3.73 | -2.75 | SigA | ComA/Spo0A |
| spo0E | -0.88 | -1.10 | -1.72 | 0.15 | SigA | AbrB |
| tlpA | -0.03 | -0.01 | -0.45 | 2.21 | SigD |  |
| cgeD | -0.49 | -0.48 | -0.04 | 1.80 | SigK | GerE |
| cgeE | -0.47 | -0.41 | 0.42 | 1.90 | SigK | GerE |
| kipI | 0.58 | 0.44 | 0.16 | 1.75 | SigK | KipR/TnrA |
| kipA | 0.45 | 0.52 | 0.03 | 2.12 | SigK | KipR/TnrA |
| spoVS | -1.35 | -1.61 | -1.21 | -0.69 | SigH |  |
| csgA | -0.78 | -1.80 | -2.31 | -1.27 | SigG | SpoVT |
| tasA | -0.05 | -0.18 | 0.23 | 2.89 | SigA/SigR | Spo0A |
| bofC | -0.61 | -0.99 | -2.26 | 1.71 | SigB/SigF/SigG | SpoVT |
| phrF | 0.74 | 0.34 | 0.21 | -2.02 | SigH | ComA |
| rapC | 1.30 | 0.93 | 0.05 | -2.93 | SigA/SigH | CodY/ComA |
| rapF | 1.64 | 0.81 | 0.90 | -2.35 | SigH | ComA |
| spo0M | -1.80 | -2.09 | -1.80 | 1.51 | SigH |  |
| sdaAB | 0.44 | 0.35 | -0.70 | -1.62 |  |  |
|  |  |  |  |  |  |  |
| **Gln-responsive genes** | | | | | | |
| cotF | -0.56 | -0.77 | -0.08 | -4.24 | sigK | spoIIID |
| yabG | -0.76 | -1.04 | -0.71 | -3.06 | sigK |  |
| sspF | -1.48 | -1.64 | -1.07 | -0.79 | sigG |  |
| spoVG | -0.84 | -1.73 | -0.97 | 0.04 | SigH | AbrB |
| spoIIE | 0.07 | -2.42 | -3.25 | -0.08 | sigA | SpoOA |
| spoVR | -0.33 | -0.63 | -0.65 | -3.21 | sigE |  |
| spoIVA | -0.49 | -1.01 | -1.38 | -3.46 | sigE | spoIIID |
| spoIVB | 0.07 | -0.52 | -0.47 | -2.67 | sigF,sigG | SpoVT |
| spoIVFA | -0.51 | -0.26 | -1.73 | -1.63 | sigE | SpoIIID |
| spoVID | -0.29 | -1.22 | -1.18 | -3.44 | sigE |  |
| usd | -0.54 | -1.00 | -0.38 | -6.06 | sigE |  |
| cotA | -0.82 | -1.79 | -1.02 | -1.92 | sigK | GerE,SpoIIID |
| cotB | -0.25 | -0.41 | -0.10 | -3.17 | sigK | GerE |
| cotC | 0.58 | 2.34 | 1.23 | -0.56 | sigK | GerE,SpoIIID |
| cotE | -0.50 | -0.74 | -0.47 | -3.55 | sigE,sigK |  |
| cotV | -0.36 | -1.38 | -1.18 | -5.37 | sigK | GerE,SpoIIID |
| cotW | -0.27 | -1.15 | -0.91 | -4.86 | sigK | GerE,SpoIIID |
| cotX | -0.27 | -1.50 | -1.14 | -5.65 | sigK | GerE,SpoIIID |
| cotY | -0.47 | -1.28 | -0.71 | -5.95 | sigK | GerE,YlbO |
| cotZ | -0.47 | -1.21 | -0.70 | -5.37 | sigK | GerE,YlbO |
| spmA | -0.13 | 0.44 | -1.61 | -2.20 | sigE |  |
| spmB | -0.19 | -0.10 | -1.91 | -2.47 | sigE |  |
| spoIIIAA | -0.45 | -0.63 | -3.12 | -3.05 | sigE | YlbO,SpoIIID |
| spoIIIAB | 0.17 | 0.16 | -2.42 | -1.89 | sigE | YlbO,SpoIIID |
| spsA | -0.48 | -0.77 | -0.67 | -2.61 | sigK |  |
| spsB | -0.48 | -0.92 | -0.75 | -4.02 | sigK |  |
| spsC | -0.33 | -0.89 | -0.71 | -3.26 | sigK |  |
| spsD | -0.23 | -0.88 | -0.72 | -3.19 | sigK |  |
| spsE | -0.28 | -0.80 | -0.69 | -2.90 | sigK |  |
| spsF | -0.34 | -0.86 | -0.69 | -2.90 | sigK |  |
| spsG | -0.34 | -0.75 | -0.64 | -2.84 | sigK |  |
| spsI | -0.27 | -0.58 | -0.51 | -2.50 | sigK |  |
| spsJ | -0.26 | -0.53 | -0.52 | -2.01 | sigK |  |
| rapA | -0.04 | -3.45 | -2.40 | 0.86 | sigA | ComA,SpoOA |
| phrA | -0.05 | -3.45 | -2.60 | 1.30 | sigA | ComA,SpoOA |
| spoIIM | -0.01 | -0.11 | -1.92 | -2.59 | sigE | YlbO |
| spoVFA | -0.31 | -0.83 | -0.44 | -5.61 | sigA,sigK |  |
| spoVFB | -0.47 | -0.95 | -0.59 | -4.43 | sigA,sigK |  |
| SspA | -0.42 | -1.08 | -0.81 | -2.84 | sigG | SpoVT |
| SspB | -0.56 | -1.12 | -0.18 | -4.08 | sigG | SpoVT |
| sspD | -0.25 | -0.58 | -0.23 | -1.65 | sigG | SpoVT |
| sspC | -0.11 | -0.13 | 0.16 | -2.66 | sigG |  |
| spoVAA | -0.34 | -0.75 | -0.36 | -3.59 | sigG | SooVT |
| spoVAB | -0.18 | -0.72 | -0.43 | -3.36 | sigG | SooVT |
| spoVAC | -0.17 | -0.79 | -0.54 | -4.32 | sigG | SooVT |
| spoVAD | -0.13 | -0.77 | -0.41 | -3.30 | sigG | SooVT |
| spoVAE | -0.12 | -0.59 | -0.53 | -2.65 | sigG | SooVT |
| tgl | -0.47 | -0.18 | -0.04 | -1.93 | sigK |  |
| ypeB | 0.02 | -0.28 | -0.13 | -2.01 | sigG |  |
| cotG | -0.31 | -1.17 | -1.09 | -5.40 | sigK | GerE,YlbO |
| cgeA | -0.29 | -1.17 | -1.00 | -5.24 | sigK | GerE,YlbO |
| cgeB | -0.19 | -0.93 | -0.69 | -4.39 | sigK | GerE,YlbO |
| cgeC | -0.30 | -0.65 | -0.49 | -4.45 | sigK | GerE |
| kipI | -1.79 | -2.84 | -2.61 | -1.54 | sigK | KipR,TnrA |
| kipA | -1.77 | -2.72 | -2.29 | -1.00 | sigK | KipR,TnrA |
| cotS | -0.28 | -0.66 | -0.16 | -2.33 | sigK |  |
| cotSA | -0.47 | -0.60 | -0.01 | -1.78 | sigK |  |
| spoIIIAC | 0.19 | 0.25 | -2.67 | -1.92 | sigE | YlbO,SpoIIID |
| spoIIIAD | 0.02 | 0.13 | -2.26 | -1.88 | sigE | YlbO,SpoIIID |
| spoIIIAE | 0.16 | 0.14 | -2.77 | -1.41 | sigE | YlbO,SpoIIID |
| spoIIIAF | -0.19 | -0.27 | -2.96 | -2.23 | sigE | YlbO,SpoIIID |
| spoIIIAG | 0.11 | 0.13 | -2.31 | -2.36 | sigE | YlbO,SpoIIID |
| spoIIIAH | -0.12 | -0.50 | -2.56 | -2.77 | sigE | YlbO,SpoIIID |
| tasA | 1.73 | -0.60 | 0.69 | -0.35 | sigA | SinR,SpoOA |
| cotH | -0.67 | -0.25 | 0.10 | -2.31 | sigK | GerE |
| cotJA | -0.47 | -0.60 | -0.96 | -4.34 | sigE | SpoIIID |
| cotJB | -0.44 | -0.62 | -0.95 | -5.10 | sigE | SpoIIID |
| cotJC | -0.36 | -0.67 | -0.93 | -4.12 | sigE | SpoIIID |
| cotM | -0.64 | -1.13 | -0.60 | -5.75 | sigK | GerE |
| bofC | 0.27 | -1.04 | -1.48 | 2.45 | sigB,sigF,sigG | SpoVT |
| sspO | -1.56 | -1.08 | -1.24 | -1.89 | sigG |  |
| sspP | -0.58 | -0.71 | 0.06 | -2.97 | sigG |  |
| rapC | -0.79 | -0.90 | -0.75 | -1.86 | sigA,sigH | CodY,ComA |
| spoIIQ | 0.01 | -0.77 | -2.31 | -3.01 | sigF |  |
| cotP | -0.53 | -0.65 | -0.46 | -3.32 | sigK | GerE |
| spo0M | -0.82 | -2.56 | -1.28 | -0.98 |  |  |
| yknT | -0.18 | -0.70 | -0.56 | -3.07 | sigE |  |
| yobW | -0.38 | -0.30 | -0.97 | -4.86 | sigK |  |
| sspI | -0.84 | -0.84 | -0.66 | -2.18 | sigG |  |
| ykvU | -0.52 | -0.49 | -2.08 | -2.49 | sigE,sigG | SpoIIID |
| coxA | -0.24 | -0.52 | -0.42 | -1.62 | sigE |  |
| ytaA | -0.42 | -0.91 | -0.59 | -4.23 | sigK |  |
| sspG | -0.18 | -0.98 | -0.80 | -2.19 | sigK | GerE |
| sspK | -0.63 | -0.82 | 0.15 | -3.79 | sigG |  |

The expression of genes dependent on GerE, involved in late spore coat genes and germination, also produced significant *T* values after 24 h of valine and glutamine addition. The repression of the GerE-dependent genes (20 genes and 31 genes with respect to Val and Gln) was shown in Fig. S2 and Table S2. Only ribonuclease III gene (*rnc*) and one spore coat protein gene (*cotC*) were induced by valine and glutamine, respectively. Three genes (*cotA, cgeD, cgeE*) were overrepresented by glutamate. Furthermore, glutamate displayed less effect on the process of sporulation and germination compared with valine and glutamine.

**
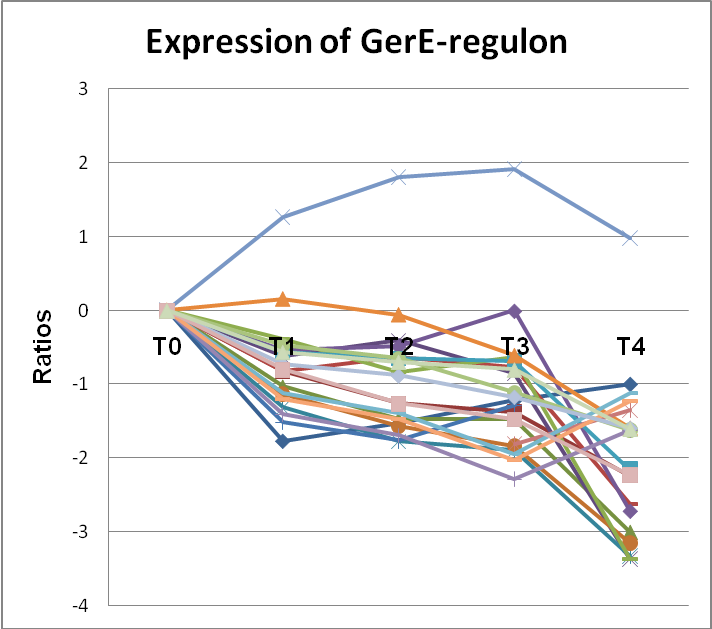

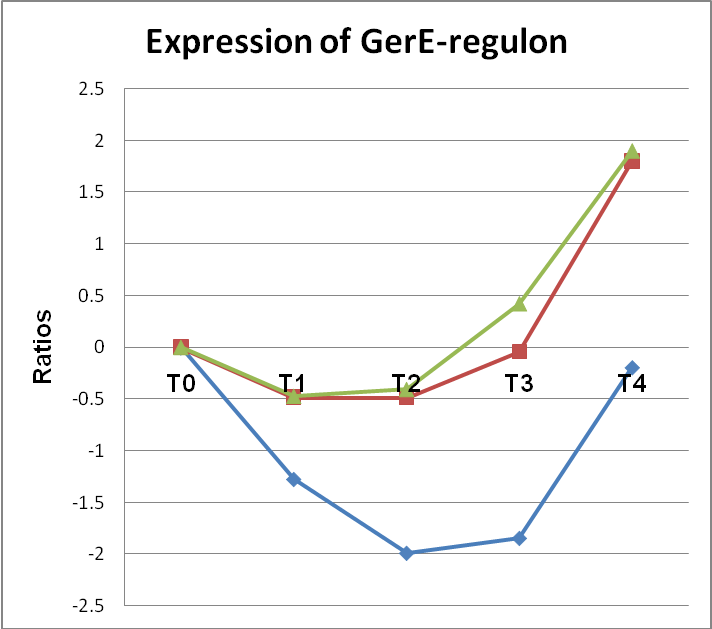

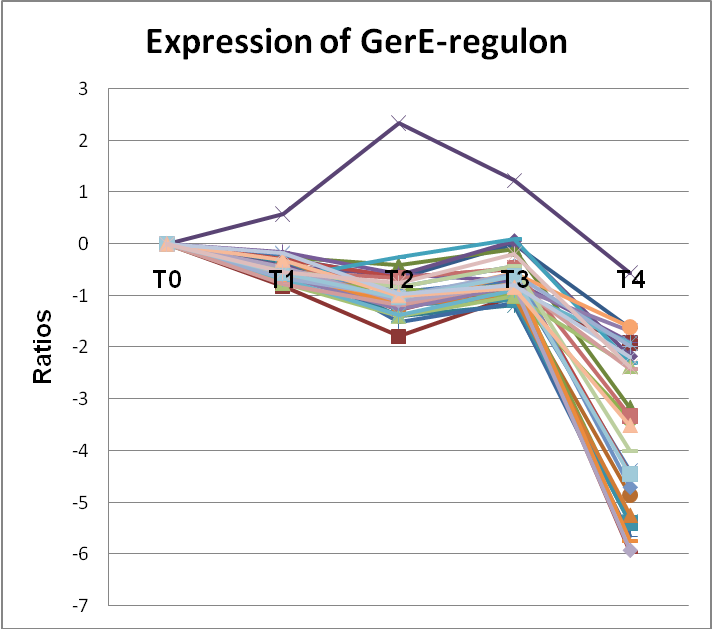
**

**Figure S2.** Expression pattern of genes of GerE-regulon after treatments with Val (A), Glu (B), and Gln (C)

**Table S2. AA-responsive genes of GerE-regulon**

| Gene Name | Ratios | | | | Sig | Reg |
| --- | --- | --- | --- | --- | --- | --- |
|  | T1 | T2 | T3 | T4 |  |  |
| **Val-responsive genes** | | | | | | |
| cotA | -1.76 | -1.52 | -1.22 | -0.99 | SigK | GerE/SpoIIID |
| cotD | -0.82 | -1.26 | -1.37 | -2.24 | SigK | GerE/SpoIIID/Spo0A |
| cotV | -1.03 | -1.47 | -1.47 | -3.00 | SigK | GerE/SpoIIID |
| cotW | -0.62 | -0.40 | -0.85 | -3.37 | SigK | GerE/SpoIIID |
| cotY | -1.32 | -1.77 | -1.89 | -3.32 | SigK | GerE/YlbO |
| cotZ | -1.13 | -1.57 | -1.84 | -3.15 | SigK | GerE/YlbO |
| cotX | -1.51 | -1.75 | -1.28 |  | SigK | GerE/SpoIIID |
| cotG | -0.82 | -0.63 | -0.78 | -2.62 | SigK | GerE/YlbO |
| cgeA | -0.39 | -0.84 | -0.63 | -3.36 | SigK | GerE/YlbO |
| cgeB | -0.54 | -0.49 | -0.01 | -2.71 | SigK | GerE/YlbO |
| cgeC | -0.56 | -0.65 | -0.68 | -2.15 | SigK | GerE |
| cgeE | 0.15 | -0.06 | -0.61 | -1.59 | SigK | GerE |
| rnc | 1.26 | 1.81 | 1.92 | 0.98 | SigA/SigK | GerE |
| cotM |  |  | -1.81 | -1.34 | SigK | GerE |
| yxeE | -0.48 | -0.65 | -1.11 | -1.62 | SigK | GerE |
| gerPF | -1.41 | -1.69 | -2.28 | -1.62 | SigK | GerE |
| gerPE | -1.13 | -1.39 | -1.94 | -1.12 | SigK | GerE |
| gerPD | -1.21 | -1.48 | -2.03 | -1.24 | SigK | GerE |
| yjcB | -0.74 | -0.88 | -1.18 | -1.60 | SigK | GerE |
| yjcC | -0.80 | -1.26 | -1.47 | -2.23 | SigK | GerE |
| yurS | -0.56 | -0.70 | -0.80 | -1.60 | SigL | GerE |
|  |  |  |  |  |  |  |
| **Glu-responsive genes** | | | | | | |
| cotA | -1.27 | -1.99 | -1.84 | -0.19 | SigK | GerE/SpoIIID |
| cgeD | -0.49 | -0.48 | -0.04 | 1.80 | SigK | GerE |
| cgeE | -0.47 | -0.41 | 0.42 | 1.90 | SigK | GerE |
|  |  |  |  |  |  |  |
| **Gln-responsive genes** | | | | | | |
| cotA | -0.82 | -1.79 | -1.02 | -1.92 | sigK | GerE,SpoIIID |
| cotB | -0.25 | -0.41 | -0.10 | -3.17 | sigK | GerE |
| cotC | 0.58 | 2.34 | 1.23 | -0.56 | sigK | GerE,SpoIIID |
| cotV | -0.36 | -1.38 | -1.18 | -5.37 | sigK | GerE,SpoIIID |
| cotW | -0.27 | -1.15 | -0.91 | -4.86 | sigK | GerE,SpoIIID |
| cotX | -0.27 | -1.50 | -1.14 | -5.65 | sigK | GerE,SpoIIID |
| cotY | -0.47 | -1.28 | -0.71 | -5.95 | sigK | GerE,YlbO |
| cotZ | -0.47 | -1.21 | -0.70 | -5.37 | sigK | GerE,YlbO |
| spoIIIC | -0.43 | -0.61 | 0.05 | -2.18 | sigE,sigK | GerE,SpoIIID |
| cotG | -0.31 | -1.17 | -1.09 | -5.40 | sigK | GerE,YlbO |
| cgeA | -0.29 | -1.17 | -1.00 | -5.24 | sigK | GerE,YlbO |
| cgeB | -0.19 | -0.93 | -0.69 | -4.39 | sigK | GerE,YlbO |
| cgeC | -0.30 | -0.65 | -0.49 | -4.45 | sigK | GerE |
| rnc | -0.71 | -0.88 | -1.01 | -3.39 | sigA,sigK | GerE |
| cwlH | -0.16 | -0.59 | -0.71 | -1.90 | sigK | GerE |
| cotH | -0.67 | -0.25 | 0.10 | -2.31 | sigK | GerE |
| cotM | -0.64 | -1.13 | -0.60 | -5.75 | sigK | GerE |
| yxeE | -0.57 | -0.82 | -0.42 | -4.70 | sigK | GerE |
| cotP | -0.53 | -0.65 | -0.46 | -3.32 | sigK | GerE |
| gerPF | -0.76 | -1.39 | -1.00 | -2.36 | sigK | GerE |
| gerPE | -0.62 | -1.26 | -0.81 | -1.69 | sigK | GerE |
| gerPD | -0.68 | -1.37 | -0.89 | -1.91 | sigK | GerE |
| gerPC | -0.61 | -1.05 | -0.56 | -1.61 | sigK | GerE |
| gerPB | -0.66 | -1.01 | -0.58 | -1.98 | sigK | GerE |
| gerPA | -0.77 | -1.18 | -0.76 | -2.43 | sigK | GerE |
| yjcB | -0.51 | -0.83 | -0.43 | -4.00 | sigK | GerE |
| yjcC | -0.45 | -1.10 | -0.78 | -5.92 | sigK | GerE |
| yoaN | -0.56 | -1.04 | -0.62 | -4.46 | sigK | GerE |
| yurS | -0.31 | -1.00 | -0.84 | -3.51 | sigK | GerE |
| sspG | -0.18 | -0.98 | -0.80 | -2.19 | sigK | GerE |
| yozR | -0.54 | -0.75 | -0.20 | -2.38 | sigK | GerE |

**2. Expression of motility-associated genes**

**Table S3. Genes involved in motility with threefold changes**

| Gene No. | Gene Name | Ratios | | | | Sig | Reg |
| --- | --- | --- | --- | --- | --- | --- | --- |
|  |  | T1 | T2 | T3 | T4 |  |  |
| **Val-responsive genes (25)** | | | | | | | |
| BG10243 | fliI | -0.77 | -0.74 | 0.06 | -1.93 | SigA/SigD | Spo0A |
| BG10245 | fliJ | 0.55 | 0.45 | 0.60 | -2.06 | SigA/SigD | Spo0A |
| BG10247 | fliK | -0.05 | -0.17 | 0.43 | -1.69 | SigA/SigD | Spo0A |
| BG10248 | flgD | -0.51 | -0.25 | 0.45 | -2.27 | SigA/SigD | Spo0A |
| BG10249 | flgG | 0.30 | -0.42 |  | -1.73 | SigA/SigD | Spo0A |
| BG10255 | cheB | 0.07 | 0.65 | 0.61 | -1.81 | SigA/SigD | Spo0A |
| BG10256 | cheA | -0.01 | 0.48 | 0.49 | -2.03 | SigA/SigD | Spo0A |
| BG10262 | fliR | 0.60 | 0.99 | 0.69 | -1.76 | SigA/SigD | Spo0A |
| BG10542 | flhA | 0.03 | 0.27 | 0.44 | -1.72 | SigA/SigD | Spo0A |
| BG10544 | flhF | 0.13 | 0.55 | 0.61 | -1.73 | SigA/SigD | Spo0A |
| BG10692 | cheW | -0.08 | 0.11 | 0.41 | -1.73 | SigA/SigD | Spo0A |
| BG10749 | cheC | 0.05 | 0.18 | 0.23 | -2.21 | SigA/SigD | Spo0A |
| BG10750 | cheD | 0.10 | 0.41 | 0.42 | -1.80 | SigA/SigD | Spo0A |
| BG10751 | sigD | 0.19 | 0.57 | 0.54 | -1.74 | SigA/SigD | Spo0A |
| BG10366 | ytxE | 1.18 | 2.03 | 1.59 | 0.84 |  |  |
| BG10399 | flgM | -0.37 | -0.70 | -0.43 | -2.05 | SigB/SigA | ComK |
| BG10655 | hag | -0.86 | -1.57 | -1.31 | -4.77 | SigD | CodY |
| BG10688 | motA | -1.34 | -1.97 | -1.41 | -2.68 | SigD |  |
| BG10689 | motB | -1.41 | -1.96 | -1.45 | -2.36 | SigD |  |
| BG10859 | mcpB | -0.01 | -0.37 | -0.50 | -1.82 | SigD |  |
| BG10861 | mcpA | -0.24 | -0.81 | -0.99 | -2.02 | SigD |  |
| BG10920 | yvyC | -0.60 | -0.53 | 0.03 | -2.03 | SigD |  |
| BG10921 | fliD | 0.26 | 0.18 | 0.74 | -1.79 | SigD |  |
| BG10922 | fliS | 0.53 | 0.30 | 0.63 | -1.63 | SigD |  |
| BG11533 | mcpC | -0.10 | -0.35 | -0.76 | -2.32 | SigD |  |
| **Glu-responsive genes (41)** | | | | | | | |
| BG10237 | flgB | 0.09 | -0.26 | 1.01 | 1.63 | SigA/SigD | Spo0A |
| BG10238 | flgC | 0.11 | -0.42 | 0.79 | 1.58 | SigA/SigD | Spo0A |
| BG10239 | fliE | 0.05 | -0.37 | 0.86 | 1.98 | SigA/SigD | Spo0A |
| BG10240 | fliF | 0.50 | -0.53 | 0.94 | 2.04 | SigA/SigD | Spo0A |
| BG10241 | fliG | 0.19 | -0.65 | 0.60 | 1.93 | SigA/SigD | Spo0A |
| BG10242 | fliH | 0.12 | -0.63 | 0.55 | 1.92 | SigA/SigD | Spo0A |
| BG10243 | fliI | 0.20 | -0.58 | 0.60 | 2.05 | SigA/SigD | Spo0A |
| BG10245 | fliJ | 0.02 | -0.48 | 0.88 | 2.19 | SigA/SigD | Spo0A |
| BG10247 | fliK | -0.19 | -0.31 | 0.94 | 2.28 | SigA/SigD | Spo0A |
| BG10248 | flgD | -0.41 | -0.16 | 1.05 | 2.50 | SigA/SigD | Spo0A |
| BG10249 | flgG | -0.56 | -0.26 | 0.82 | 2.33 | SigA/SigD | Spo0A |
| BG10250 | fliL | -0.21 | -0.02 | 1.07 | 2.20 | SigA/SigD | Spo0A |
| BG10251 | fliM | -0.06 | -0.17 | 1.19 | 2.09 | SigA/SigD | Spo0A |
| BG10252 | fliY | -0.24 | -0.07 | 1.14 | 1.98 | SigA/SigD | Spo0A |
| BG10255 | cheB | 0.02 | 0.16 | 1.61 | 2.59 | SigA/SigD | Spo0A |
| BG10256 | cheA | -0.08 | -0.02 | 1.08 | 2.34 | SigA/SigD | Spo0A |
| BG10258 | cheY | -0.43 | 0.07 | 1.47 | 2.92 | SigA/SigD | Spo0A |
| BG10259 | fliZ | 0.10 | 0.00 | 1.31 | 2.42 | SigA/SigD | Spo0A |
| BG10260 | fliP | 0.14 | 0.17 | 1.34 | 2.68 | SigA/SigD | Spo0A |
| BG10261 | fliQ | 0.00 | 0.10 | 1.28 | 2.61 | SigA/SigD | Spo0A |
| BG10262 | fliR | 0.10 | 0.04 | 1.47 | 2.74 | SigA/SigD | Spo0A |
| BG10542 | flhA | -0.25 | -0.06 | 1.05 | 2.80 | SigA/SigD | Spo0A |
| BG10543 | flhB | -0.04 | 0.13 | 1.26 | 2.67 | SigA/SigD | Spo0A |
| BG10544 | flhF | -0.15 | -0.02 | 1.17 | 2.78 | SigA/SigD | Spo0A |
| BG10692 | cheW | 0.03 | 0.07 | 1.11 | 2.32 | SigA/SigD | Spo0A |
| BG10750 | cheD | 0.45 | 0.29 | 1.28 | 2.37 | SigA/SigD | Spo0A |
| BG10751 | sigD | 0.30 | 0.20 | 1.11 | 2.18 | SigA/SigD | Spo0A |
| BG10366 | ytxE | 1.79 | 1.13 | 0.81 | -1.12 |  |  |
| BG10655 | hag | -1.25 | -1.00 | -0.70 | 2.28 | SigD | CodY |
| BG10688 | motA | -0.44 | -0.30 | -0.20 | 2.19 | SigD |  |
| BG10689 | motB | -0.51 | -0.45 | -0.57 | 2.01 | SigD |  |
| BG10823 | cheV | -0.84 | -0.74 | -0.26 | 2.41 | SigD |  |
| BG10859 | mcpB | -0.52 | 0.13 | 0.12 | 1.77 | SigD |  |
| BG10860 | tlpA | -0.03 | -0.01 | -0.45 | 2.21 | SigD |  |
| BG10861 | mcpA | -0.75 | -0.50 | -0.44 | 2.36 | SigD |  |
| BG10917 | flhO | 0.03 | -0.11 | -0.03 | 1.64 | sigE |  |
| BG10921 | fliD | 0.69 | 0.28 | 0.83 | 1.61 | SigD |  |
| BG10922 | fliS | 0.21 | 0.13 | 0.59 | 1.99 | SigD |  |
| BG10923 | fliT | 0.25 | 0.17 | 0.49 | 2.18 | SigD |  |
| BG11533 | mcpC | -0.54 | -0.59 | -0.19 | 2.58 | SigD |  |
| BG13066 | hemAT | -0.68 | -0.46 | -0.65 | 1.90 | SigD |  |
| **Gln-responsive genes (3)** | | | | | | | |
| BG10365 | ytxD | 0.79 | 2.02 | 1.28 | -0.95 |  |  |
| BG10366 | ytxE | 0.04 | 2.77 | 0.97 | -1.23 |  |  |
| BG10655 | hag | -0.87 | -1.87 | 0.58 | -0.92 | sigD | CodY |

Twenty-three of the 56 known flagellar and chemotaxis genes (*motAB, hag, fliDST, fla/che* operon, *mcpABC*) were expressed at lower levels 8 h after valine pulse; the majority of these genes are regulated by sigma factor SigD (Fig.S3A). Forty-one genes (*motAB, hag, fliDST, fla/che* operon, *mcpABC*) involved in motility were differently induced at 24 h following glutamate addition (Fig.S3B). Interestingly, we observed major changes in *motAB* and *ytxDE (motPS)* genes involved in rotary motor of bacterial flagellum upon amino acids addition (Fig.S3C).


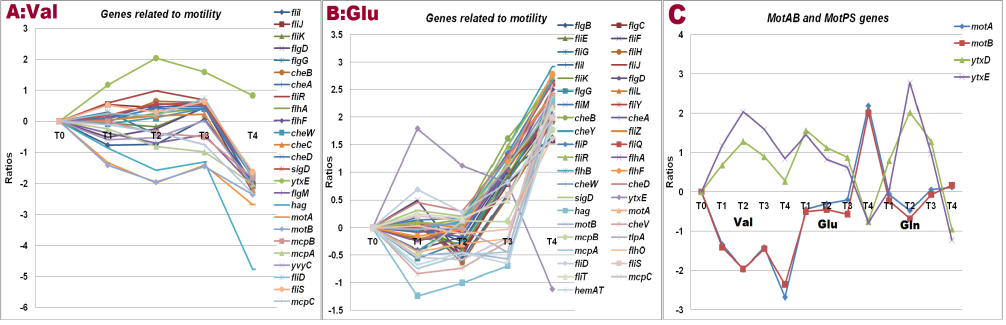


**Figure S3. The expression profiles of genes related to motolity**

The genes with threefold changes were shown after treatment of valine (A) and glutamate (B). (C) The different expression patterns of *motAB* and *motPS* genes under the conditions of three amino acids treatments.

**3. Expression of cell-wall-associated genes**

The SigW regulon of *B. subtilis* has been functionally implicated in cell-wall-associated processes and the adaptation to alkaline shock, salt shock, phage infection and certain antibiotics that affect cell wall biosynthesis. We found 22 of the 62 SigW regulon members to be significantly induced (at 30 min and 2 h) after Val addition, whereas *yfh* operon, *yxjJ, spxA* genes to be repressed; 23 and 28 genes to be down-expressed after Glu and Gln addition. Most of these overrepresented genes are unassigned-function genes excepting *xpaC, pspA, yndN, sppA, pbpX, spxA, pbpE, racX, sigW* genes. We also observed the significant induction of genes regulated by YvrH after treatment with valine and glutamate in T-profiler analysis. The YvrG-YvrH two-component system appears to be related to cell membrane and cell wall function. It is reported that the YvrGH system positively regulates the seven transcriptional units (*wprA, wapA-yxxG, dltABCDE, sunA, sunT-bdbA-yolJ-bdbB, yvrI-yvrHa,* and *sigX-rsiX*), and negatively regulates the *lytABC* operon. *wprA, wapA, lytB, and lytC* encode the main cell surface proteins of *B. subtilis*. *lytC* encodes an N-acetylmuramoyl-L-alanineamidase, and it is known as a major autolytic enzyme. Furthermore, SigX sigma factor relating to the cell surface homeostatic functions, was regulated by this system. Masakuni Serizawa *et al*. noted that the *yvrGHb* null mutant showed the unusual autolysis and higher susceptibility to the four kinds of antibiotics (aztreonam, cefepime, bacitracin, and fosfomycin). Indeed, *sunT, sunA*, *wapA-yxxG,* and *dlt* genes were upregulated, while *yvrI, sigX,* and *lytA* genes were downregulated (Fig.S4).


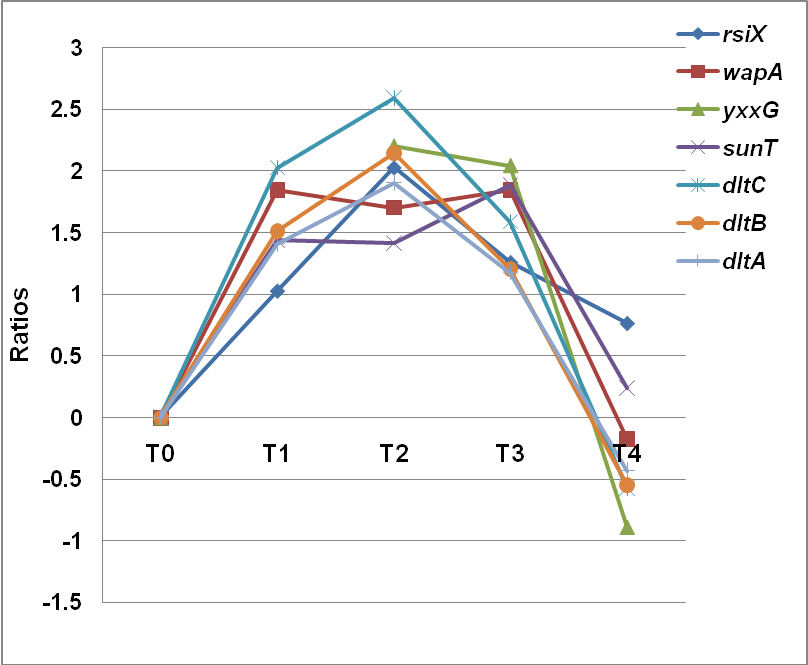

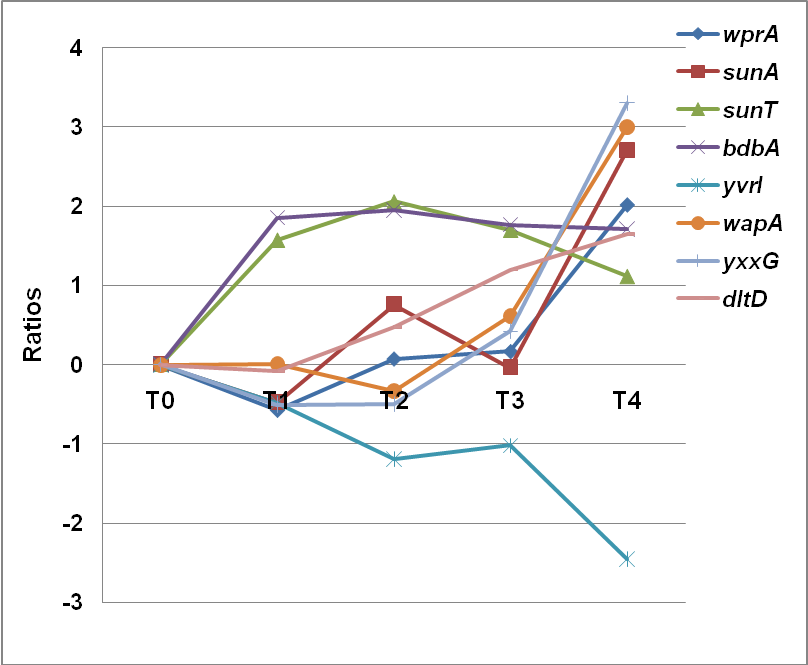

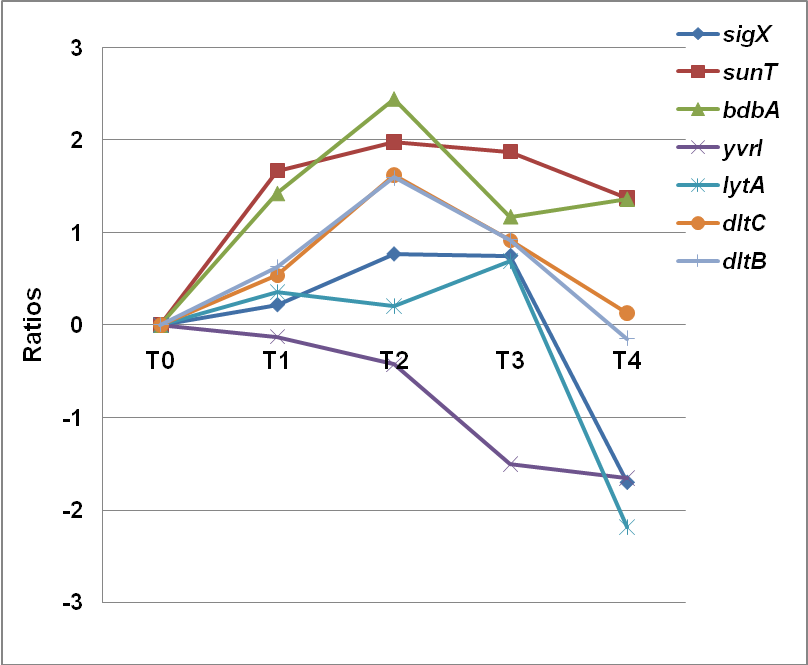


Figure S4. Expression pattern of genes of *yvrH*-regulon after treatments with Val (A), Glu (B), and Gln (C).

**4. Cysteine/Methionine metabolism and sulfur metabolism**

Sulfur is a crucial atom in cysteine and methionine, as well as in several coenzymes and cofactors such as thiamine, biotin, or coenzyme A (CoA). In *E. coli* and *B. subtilis*, among the ca 4100 and 4,500 genes in the genome, more than one hundred genes are directly involved in some step of sulfur metabolism. In this study, we observe that three amono acids all activate the sulfur metabolism, including the assimilation of sulfur, biosynthesis of cysteine and methionine, and methionine salvage process (Table S4).

**Table S4. AA-responsive g**enes related sulfur, methionine, and cysteine metabolism

| Gene Name | Ratios | | | | Sig | Reg |
| --- | --- | --- | --- | --- | --- | --- |
|  | T1 | T2 | T3 | T4 |  |  |
| **Val-responsive genes** | | | | | | |
| spxA | -1.00 | -1.46 | -1.60 | -0.90 | SigA/SigB/SigW/SigX/SigM | PerR/YodB |
| yhcL | 1.40 | 1.62 | 0.03 | -0.01 | SigA | YrzC |
| yxeK | 1.55 | 1.60 | 0.18 | -0.59 | SigA | YrzC/YjbD |
| yxeL | 1.55 | 1.61 | -0.04 | -0.57 | SigA | YrzC/YjbD |
| yxeM | 1.83 | 1.88 | 0.20 | -0.29 | SigA | YrzC/YjbD |
| yxeP | 1.38 | 1.72 | -0.55 | -0.17 | SigA | YrzC/YjbD |
| ydbM | -0.98 | -1.60 | -1.71 | -1.09 |  | YrzC |
| yrhA | 2.03 | 1.69 | -0.53 | -0.65 | SigA | YrzC |
| yrhB | 1.77 | 1.78 | -0.66 | -0.56 | SigA | YrzC |
| yrrT | 2.91 | 2.76 | -0.52 | -0.56 | SigA | YrzC |
| mtn | 1.81 | 1.74 | 0.88 | -0.21 | SigA | YrzC |
| yrhC | 1.51 | 1.68 | -0.62 | -0.12 | SigA | YrzC |
| ssuA | -0.19 | 2.43 | -0.36 | 0.10 | SigA | YjbD/YrzC |
| ssuD | -0.17 | 2.11 | -0.33 | -0.11 | SigA | YjbD/YrzC |
| ygaN | -0.51 | 1.79 | 0.08 | 0.17 | SigA | YjbD/YrzC |
| cysH | 1.72 | 1.81 | -0.06 | -0.09 | SigA |  |
| ylnE | 1.66 | 1.98 | -0.17 | -0.50 | SigA |  |
| ylnF | 1.19 | 1.63 | -0.36 | -0.41 | SigA |  |
| speE | 0.89 | 1.92 | 1.68 | 0.52 | SigF/SigG |  |
| metE | 1.18 | 1.92 | 0.56 | -0.11 |  |  |
| mtnA | 1.05 | 2.47 | 0.77 | -0.16 | SigA |  |
| mtnK | 1.56 | 2.55 | 1.25 | 0.13 | SigA |  |
| mtnW | -0.09 | 1.70 | 0.39 | 0.79 | SigA |  |
| mtnX | 0.09 | 2.29 | 0.42 | 0.53 | SigA |  |
| mtnB | -0.12 | 1.81 | 0.27 | 0.20 | SigA |  |
| ykrZ | -0.20 | 2.19 | 0.22 | 0.28 | SigA |  |
| speD | 0.82 | 1.81 | 0.77 | 0.52 | SigA | YqzB |
| yvgQ | 0.11 | 1.84 | 0.02 | -0.64 | SigA | YwfK |
| **Glu-responsive genes** | | | | | | |
| yhcL | -0.20 | 2.07 | 0.81 | 1.07 | SigA | YrzC |
| yxeK | 0.00 | 3.84 | 1.48 | 1.56 | SigA | YrzC/YjbD |
| yxeL | -0.49 | 3.14 | 1.13 | 1.40 | SigA | YrzC/YjbD |
| yxeM | -0.17 | 3.81 | 1.75 | 1.90 | SigA | YrzC/YjbD |
| yxeN | -0.72 | 3.25 | 0.86 | 1.69 | SigA | YrzC/YjbD |
| yxeO | -0.61 | 2.73 | 0.92 | 1.91 | SigA | YrzC/YjbD |
| yxeP | -0.72 | 2.99 | 0.80 | 1.83 | SigA | YrzC/YjbD |
| yxeQ | -0.55 | 2.44 | 0.36 | 1.61 | SigA | YrzC/YjbD |
| yrhA | 0.35 | 1.67 | 0.80 | 0.54 | SigA | YrzC |
| yrrT | 0.56 | 2.69 | 1.62 | 1.03 | SigA | YrzC |
| ssuA | -0.39 | 0.95 | 0.06 | 1.68 | SigA | YjbD/YrzC |
| ssuB | -0.64 | 1.18 | 0.12 | 1.60 | SigA | YjbD/YrzC |
| ytlI | 0.89 | 2.23 | 0.85 | 0.49 | SigA | YjbD/YrzC |
| ytmJ | 0.40 | 1.63 | 0.20 | 1.32 | SigA | YjbD/YrzC/YtlI |
| ytmK | 0.71 | 1.58 | 0.74 | 0.70 | SigA | YjbD/YrzC/YtlI |
| ytnL | 0.47 | 0.51 | 0.68 | 3.02 | SigA | YjbD/YtlI/YrzC |
| cysH | -0.04 | 2.33 | 0.87 | 0.56 | SigA |  |
| metE | -0.74 | 2.32 | 2.27 | 0.61 |  |  |
| mtnA | -0.86 |  | 2.43 | 0.83 | SigA |  |
| mtnK | -0.25 | 3.02 | 2.71 | 0.75 | SigA |  |
| cysP | 0.07 | 2.16 | 0.75 | 0.32 | SigA |  |
| sat | 0.10 | 2.19 | 1.22 | 0.89 | SigA |  |
| cysC | -0.26 | 1.89 | 0.95 | 1.04 | SigA |  |
| speD | 1.23 | 1.51 | 1.32 | -2.31 | SigA | YqzB |
| **Gln-responsive genes** | | | | | | |
| yhcL | 0.72 | 3.16 | 0.76 | 1.11 | sigA | YrzC |
| yxeK | 1.24 | 3.78 | 1.74 | -0.58 | sigA | YrzC,YjbD |
| yxeL | 1.05 | 3.74 | 1.31 | 0.25 | sigA | YrzC,YjbD |
| yxeM | 1.48 | 4.22 | 1.70 | 0.58 | sigA | YrzC,YjbD |
| yxeN | 0.67 | 4.06 | 1.12 | 0.72 | sigA | YrzC,YjbD |
| yxeO | 0.81 | 2.79 | 1.02 | 0.74 | sigA | YrzC,YjbD |
| yxeP | 0.69 | 3.42 | 0.94 | 0.65 | sigA | YrzC,YjbD |
| yxeQ | 0.26 | 3.41 | 0.71 | 0.53 | sigA | YrzC,YjbD |
| yxeR | -0.07 | 2.15 | 0.23 | -0.24 | sigA | YrzC,YjbD |
| yrhA | 0.31 | 2.62 | 0.52 | 0.74 | sigA | YrzC |
| yrhB | 0.10 | 2.38 | 0.10 | 0.89 | sigA | YrzC |
| yrhC | -0.63 | 1.75 | -0.49 | 0.65 | sigA | YrzC |
| yrrT | 0.55 | 3.20 | 1.08 | 0.18 | sigA | YrzC |
| ssuB | -1.70 | 1.01 | -0.78 | -0.11 | sigA | YjbD,YrzC |
| ytlI | -0.17 | 2.26 | 0.49 | 0.13 | sigA | YjbD,YrzC |
| ytmI | -0.65 | -0.13 | -0.89 | 2.06 | sigA | YjbD,YtlI,YrzC |
| ytmJ | 0.21 | 2.47 | 0.31 | 2.55 | sigA | YjbD,YtlI,YrzC |
| ytmK | 1.03 | 2.17 | 0.52 | 1.65 | sigA | YjbD,YtlI,YrzC |
| ytmL | 0.89 | 1.33 | 1.65 | 1.42 | sigA | YjbD,YtlI,YrzC |
| ytmM | 1.00 | 1.77 | -0.19 | 0.76 | sigA | YjbD,YtlI,YrzC |
| ytnM | 1.59 | 1.52 | 0.82 | 0.03 | sigA | YjbD,YtlI,YrzC |
| yxjH | 0.41 | 1.37 | 1.71 | 0.03 |  |  |
| cysH | 0.15 | 2.24 | 0.56 | -1.14 | sigA |  |
| cysP | 0.12 | 2.01 | 0.44 | -0.76 | sigA |  |
| sat | 0.68 | 2.26 | 0.75 | -0.14 | sigA |  |
| ylnE | 0.16 | 1.97 | 0.45 | 0.15 | sigA |  |
| ylnF | -0.22 | 1.67 | 0.19 | 0.16 | sigA |  |
| speE | 0.66 | 1.98 | 1.39 | -1.32 | sigF,sigG |  |
| speD | 0.60 | 1.65 | 1.22 | -2.66 | sigA | YqzB |
| metE | 1.08 | 2.82 | 3.01 | -0.66 |  |  |
| yisZ | -0.58 | -0.56 | -0.23 | -2.80 | sigK |  |
| yitB | -0.60 | -0.67 | -0.21 | -3.34 | sigK |  |
| yjcI | 0.95 | 1.81 | 2.16 | -0.20 | sigA |  |
| yjcJ | 0.95 | 1.80 | 2.06 | 0.15 | sigA |  |
| mtnA | 0.91 | 3.19 | 2.61 | -0.69 | sigA |  |
| mtnK | 1.43 | 3.71 | 3.38 | -0.78 | sigA |  |
| ykrV | 0.53 | 1.78 | 1.56 | -0.13 | sigA |  |
| mtnW | 0.68 | 2.30 | 1.66 | -0.10 | sigA |  |
| mtnX | 0.75 | 2.17 | 1.74 | 0.29 | sigA |  |
| mtnB | 0.68 | 1.87 | 1.61 | 0.57 | sigA |  |
| ykrZ | 0.48 | 1.89 | 1.45 | 0.50 | sigA |  |

**5. Phosphate metabolism**

In addition to the sulfur metabolism-related gene functions, we observed a significant expression change of the gene group regulated by the central phosphate-regulatory protein PhoP. Valine, glutamate, and glutamine induced the expression of *pst* operon (Fig. S5A) at 5 min, 30 min, and 24 h, respectively. In some organisms, such as *S. lividans* and *S. coelicolor*, certain carbon sources (fructose, galactose or mannose) can activate the expression of pst operon. *pit* gene also was up-regulated by three amino acids (Fig.S5B). In addition, all the *ybcPST* and *ybdABDE* genes of *skf* operon regulated by PhoP, Spo0A, and AbrB diplayed significant and transient (5-30 min) induction immediately after valine pulse (Fig.S5C), and produced the sporulation killing factor to lyze sister cells; all the *tuaABCDEFGH* genes of *tua* operon diplayed significant and transient (30-120 min) induction after glutamate pulse, and produced teichuronic acid (Fig.S5D). In *B. subtilis*, *ybhH* gene of SigE-regulon, encoding a sporulation protein, was induced during phosphate deprivation. In this study, *yhbH* gene was clearly repressed after treatment with valine and glutamine (Fig.S5E). These combined data revealed the obvious induction of some genes of PhoP-regulon may not result from phosphate starvation, may exist a connection between phosphate and amino acid regulation, which should be further investigated in the future.


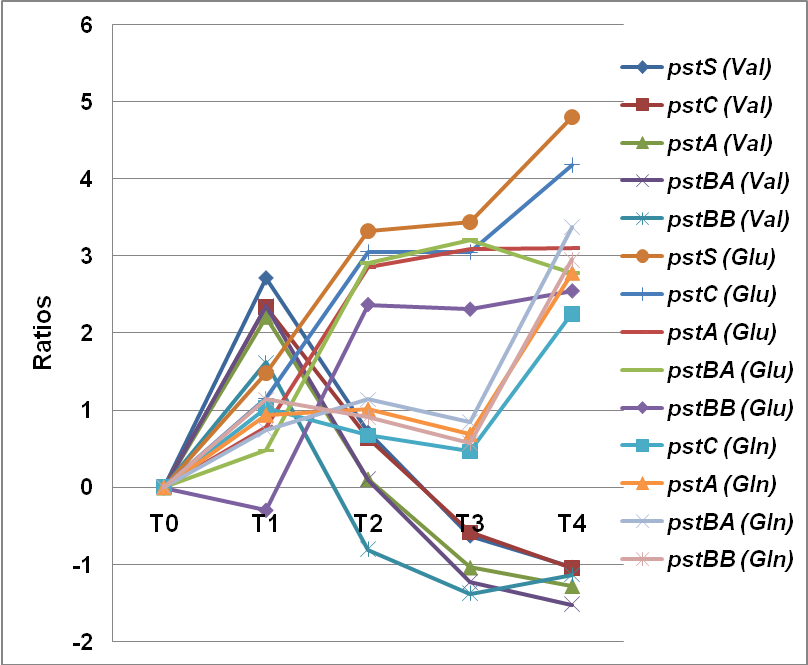

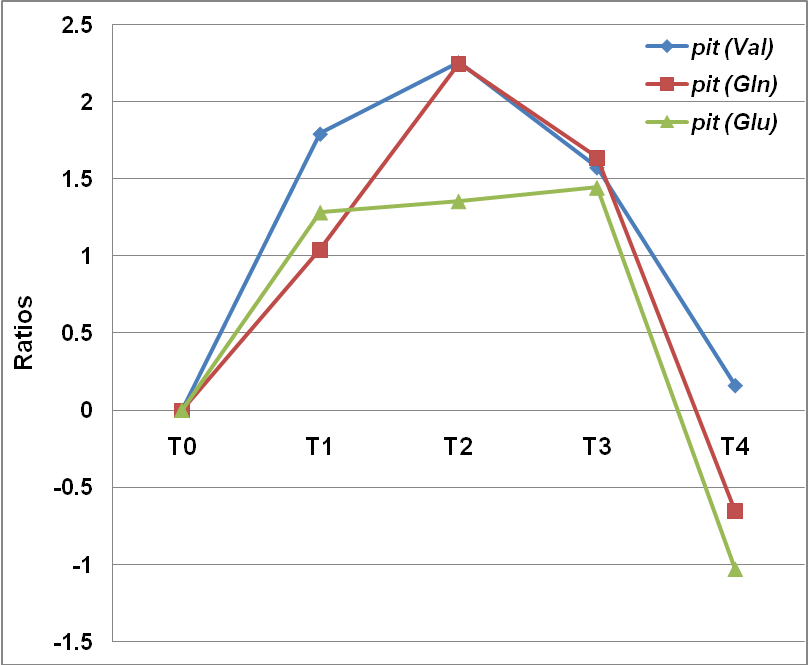

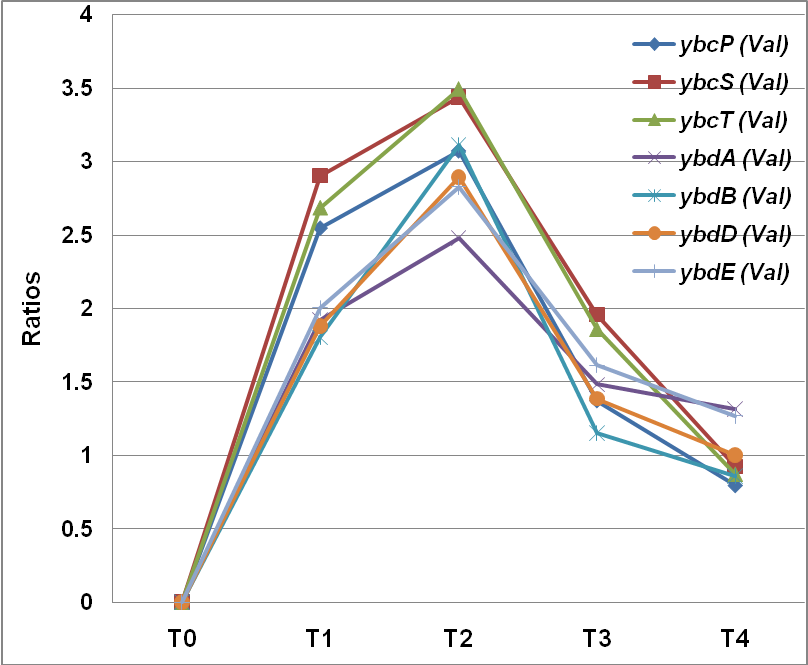

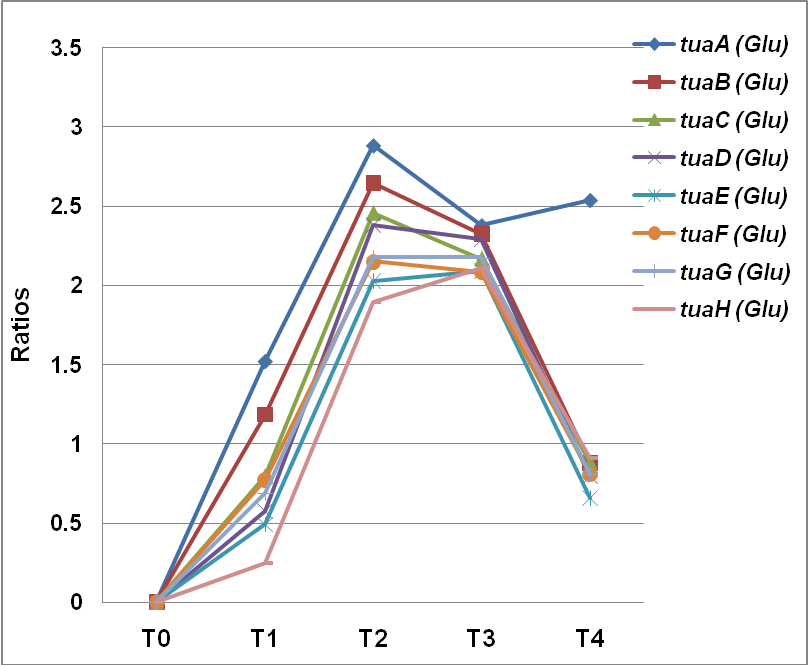

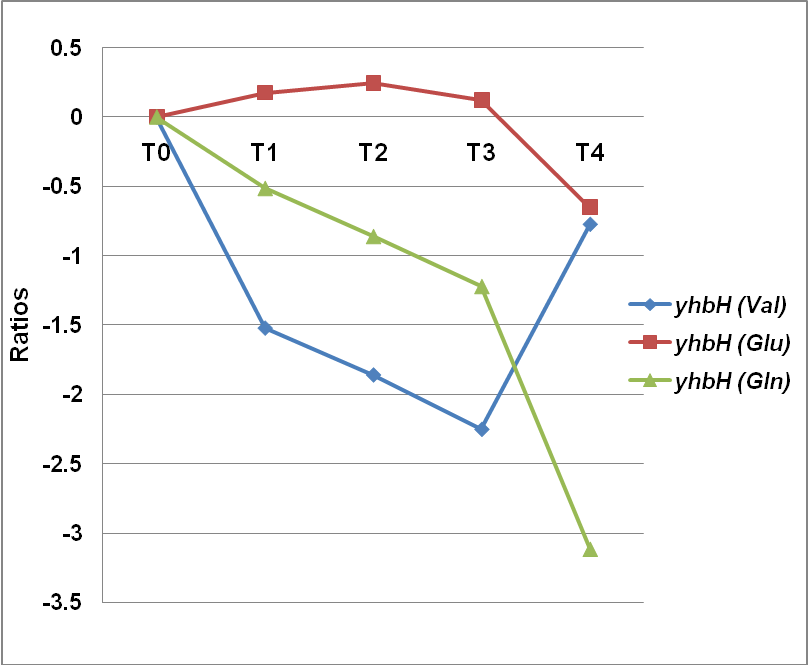


Figure S5. Expression pattern of genes of *pst* operon (A), *pit* gene (B), *ybc-ybd* operon (C), *tua* operon (D), and *yhbH* gene (E) after treatments with amino acids.

**6. Transcriptional Factor and two-component regulatory systems**

The *B. subtilis* genome probably encodes 317 transcription factors or transcriptional regulators (http://dbtbs.hgc.jp/). TFs are of special interest since they are capable of coordinating the expression of several or many downstream target genes and, hence, entire metabolic and developmental pathways. Of the approximately all potential TFs on the BSU microarray, 27, 46, and 53 showed marked (three-fold) changes in transcript abundance (Figure S6 and Table S5) in response to three amino acids (Val, Glu, and Gln).


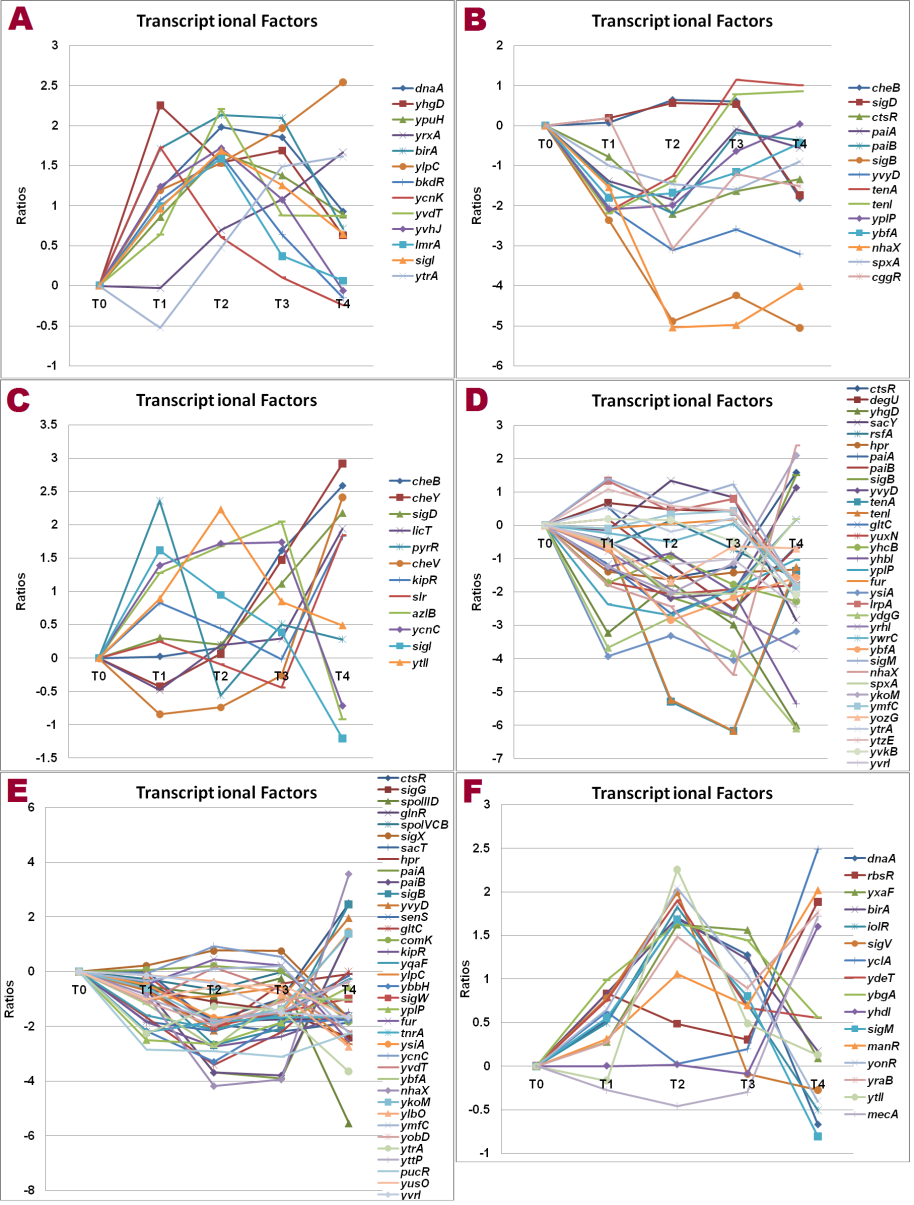


**Figure S6. Expression pattern of genes of TFs response to amino acids**

The expression profiles of TFs genes were shown after treatments with Val (AB), Glu (CD), and Gln (EF).

**Table S5. Transcriptional regulators with threefold changes**

| Gene Name | | Ratios | | | | Description |
| --- | --- | --- | --- | --- | --- | --- |
|  |  | T1 | T2 | T3 | T4 |  |
| **Val-responsive TF genes** | | | | | | |
| dnaA | | 1.20 | 1.98 | 1.85 | 0.93 | chromosomal replication initiation protein |
| yhgD | | 2.25 | 1.54 | 1.69 | 0.63 | unknown; similar to transcriptional regulator (TetR/AcrR family) |
| ypuH | | 0.86 | 1.66 | 1.38 | 0.89 | hypothetical protein |
| yrxA | | -0.03 | 0.70 | 1.08 | 1.67 | unknown; similar to transcriptional regulator |
| birA | | 1.71 | 2.13 | 2.09 | 0.71 | transcriptional regulator and biotin acetyl-CoA-carboxylase synthetase |
| ylpC | | 1.19 | 1.53 | 1.97 | 2.54 | fatty acid biosynthesis transcriptional regulator |
| bkdR | | 1.07 | 1.64 | 0.64 | -0.15 | transcriptional regulator (sigma-L-dependent) |
| ycnK | | 1.73 | 0.61 | 0.10 | -0.24 | unknown; similar to transcriptional regulator (DeoR family) |
| yvdT | | 0.64 | 2.21 | 0.88 | 0.87 | unknown; similar to transcriptional regulator (TetR/AcrR family) |
| yvhJ | | 1.24 | 1.72 | 1.08 | -0.06 | unknown; similar to transcriptional regulator |
| lmrA | | 0.99 | 1.59 | 0.37 | 0.06 | transcriptional regulator |
| sigI | | 0.96 | 1.69 | 1.26 | 0.65 | putative RNA polymerase sigma factor SigI |
| ytrA | | -0.52 | 0.47 | 1.49 | 1.61 | unknown; similar to transcriptional regulator (GntR family) |
| cheB | | 0.07 | 0.65 | 0.61 | -1.81 | chemotaxis-specific methylesterase |
| sigD | | 0.19 | 0.57 | 0.54 | -1.74 | RNA polymerase sigma factor SigD |
| ctsR | | -0.78 | -2.21 | -1.64 | -1.34 | transcriptional regulator |
| paiA | | -1.39 | -1.85 | -0.09 | -0.56 | transcriptional regulator |
| paiB | | -1.46 | -2.19 | -0.17 | -0.36 | transcriptional regulator |
| sigB | | -2.36 | -4.88 | -4.24 | -5.05 | RNA polymerase sigma factor SigB |
| yvyD | | -2.03 | -3.11 | -2.59 | -3.21 | unknown; similar to sigma-54 modulating factor of gram-negative bacteria |
| tenA | | -2.15 | -1.26 | 1.15 | 1.02 | transcriptional regulator |
| tenI | | -2.19 | -1.40 | 0.78 | 0.86 | transcriptional regulator TenI |
| yplP | | -2.09 | -1.99 | -0.64 | 0.04 | unknown; similar to transcriptional regulator (sigma-L-dependent) |
| ybfA | | -1.81 | -1.68 | -1.17 | -0.44 | hypothetical protein |
| nhaX | | -1.54 | -5.04 | -4.98 | -4.01 | putative regulatory gene for NhaC |
| spxA | | -1.00 | -1.46 | -1.60 | -0.90 | transcriptional regulator Spx |
| cggR | | 0.20 | -3.08 | -1.21 | -1.51 | transcriptional regulator |
| **Glu-responsive TF genes** | | | | | | |
| ctsR | | -0.45 | -1.58 | -1.24 | 1.58 | transcriptional regulator |
| degU | | 0.68 | 0.48 | 0.42 | -1.84 | two-component response regulator |
| yhgD | | -3.23 | -1.72 | -2.98 | -6.01 | unknown; similar to transcriptional regulator (TetR/AcrR family) |
| sacY | | -0.14 | 1.34 | 0.84 | -2.85 | transcriptional antiterminator |
| rsfA | | -0.62 | 0.14 | -0.73 | -1.59 | probable transcriptional regulatory protein |
| hpr | | -1.38 | -1.63 | -1.41 | -1.33 | transcriptional regulator (MarR family); |
| paiA | | 0.55 | -1.17 | -2.55 | -0.62 | transcriptional regulator |
| paiB | | 0.19 | -1.20 | -2.51 | -0.67 | transcriptional regulator |
| sigB | | -0.52 | -2.16 | -2.74 | 1.50 | RNA polymerase sigma factor SigB |
| yvyD | | -1.26 | -2.19 | -2.02 | 1.14 | hypothetical protein |
| tenA | | -0.53 | -5.29 | -6.18 | -1.37 | transcriptional regulator |
| tenI | | -0.50 | -5.23 | -6.17 | -1.25 | transcriptional regulator TenI |
| gltC | | -1.17 | -2.64 | -1.91 | 0.18 | transcriptional regulator (LysR family) |
| yuxN | | -1.70 | -2.06 | -1.91 | -1.79 | hypothetical protein |
| yhcB | | -1.74 | -0.91 | -1.78 | -2.28 | unknown; similar to trp repressor binding protein |
| yhbI | | -1.26 | -0.84 | -2.01 | -5.35 | unknown; similar to transcriptional regulator (MarR family) |
| yplP | | -2.37 | -2.70 | -1.91 | -1.03 | unknown; similar to transcriptional regulator (sigma-L-dependent) |
| fur | | -0.10 | 0.05 | 0.17 | -1.76 | transcriptional regulator (Fur family |
| ysiA | | -3.93 | -3.31 | -4.05 | -3.18 | unknown; similar to transcriptional regulator (TetR/AcrR family) |
| lrpA | | 1.34 | 0.41 | 0.79 | -1.83 | transcriptional regulator (Lrp/AsnC family); |
| ydgG | | -3.67 | -2.76 | -3.83 | -6.10 | unknown; similar to transcriptional regulator (MarR family) |
| yrhI | | -0.78 | -1.93 | -2.71 | -3.70 | unknown; similar to transcriptional regulator (TetR/AcrR family) |
| ywrC | | -0.24 | -0.50 | 0.05 | -1.73 | unknown; similar to transcriptional regulator (Lrp/AsnC family) |
| ybfA | | -0.71 | -2.85 | -2.17 | -1.56 | hypothetical protein |
| sigM | | 1.40 | 0.66 | 1.23 | -1.84 | RNA polymerase sigma factor SigM |
| nhaX | | -1.79 | -2.43 | -4.49 | 2.40 | putative regulatory gene for NhaC |
| spxA | | -0.54 | -2.06 | -2.05 | 0.18 | transcriptional regulator Spx |
| ykoM | | -1.24 | -2.04 | -1.14 | 2.10 | unknown; similar to transcriptional regulator (MarR family) |
| ymfC | | -0.10 | 0.32 | 0.43 | -1.84 | unknown; similar to transcriptional regulator (GntR family) |
| yozG | | -0.62 | -1.69 | -0.64 | -0.70 | unknown; similar to transcriptional regulator |
| ytrA | | 0.55 | -0.19 | 0.23 | -2.19 | unknown; similar to transcriptional regulator (GntR family) |
| ytzE | | 1.08 | 0.58 | 0.46 | -1.70 | unknown; similar to transcriptional regulator (DeoR family) |
| yvkB | | 0.19 | 0.15 | -0.48 | -2.08 | unknown; similar to transcriptional regulator (TetR/AcrR family) |
| yvrI | | -0.48 | -1.19 | -1.02 | -2.45 | hypothetical protein |
| cheB | | 0.02 | 0.16 | 1.61 | 2.59 | chemotaxis-specific methylesterase |
| cheY | | -0.43 | 0.07 | 1.47 | 2.92 | two-component response regulator |
| sigD | | 0.30 | 0.20 | 1.11 | 2.18 | RNA polymerase sigma factor SigD |
| licT | | -0.48 | 0.20 | 0.29 | 1.95 | transcriptional antiterminator(BglG family) |
| pyrR | | 2.36 | -0.56 | 0.51 | 0.28 | pyrimidine regulatory protein PyrR |
| cheV | | -0.84 | -0.74 | -0.26 | 2.41 | modulation of CheA activity in response to attractants (chemotaxis) |
| kipR | | 0.83 | 0.45 | -0.02 | 1.82 | transcriptional regulator (IclR family); |
| slr | | 0.25 | -0.10 | -0.44 | 1.85 | transcriptional regulator |
| azlB | | 1.28 | 1.68 | 2.05 | -0.92 | transcriptional regulator (Lrp/AsnC family); |
| ycnC | | 1.39 | 1.72 | 1.74 | -0.72 | unknown; similar to transcriptional regulator (TetR/AcrR family) |
| sigI | | 1.62 | 0.95 | 0.38 | -1.21 | putative RNA polymerase sigma factor SigI |
| ytlI | | 0.89 | 2.23 | 0.85 | 0.49 | unknown; similar to transcriptional regulator (LysR family) |
| **Gln-responsive TF genes** | | | | | | |
| ctsR | -0.38 | | -1.79 | -0.99 | 2.49 | transcriptional regulator |
| sigG | -0.65 | | -1.11 | -1.44 | -2.41 | sporulation sigma factor SigG |
| spoIIID | -0.53 | | -0.86 | -0.25 | -5.55 | transcriptional regulator |
| glnR | -2.20 | | -1.81 | -1.75 | -1.74 | negative regulation of the glutamine synthetase gene (glnA) |
| spoIVCB | -0.28 | | -0.65 | -0.01 | -1.63 | RNA polymerase sporulation-specific sigma factor (sigma-K) (N-terminal half) |
| sigX | 0.22 | | 0.77 | 0.75 | -1.70 | RNA polymerase sigma factor SigX |
| sacT | 0.01 | | -1.94 | -2.19 | -1.87 | transcriptional antiterminator |
| hpr | -0.60 | | -2.15 | -0.45 | -0.10 | transcriptional regulator (MarR family); |
| paiA | -0.67 | | -3.70 | -3.91 | 1.37 | negative regulation of sporulation, septation and degradative enzyme genes (aprE, nprE, phoA, sacB) |
| paiB | -0.67 | | -3.69 | -3.80 | 1.35 | negative regulation of sporulation and degradative enzyme genes |
| sigB | -0.44 | | -2.68 | -2.08 | 2.47 | RNA polymerase sigma factor SigB |
| yvyD | -0.69 | | -2.16 | -0.99 | 1.97 | unknown; similar to sigma-54 modulating factor of gram-negative bacteria |
| senS | -1.82 | | -2.17 | -1.48 | -0.14 | transcriptional regulator |
| gltC | -1.68 | | -3.40 | -2.21 | 0.01 | transcriptional regulator (LysR family) |
| comK | 0.07 | | 0.21 | 0.03 | -1.82 | competence transcription factor (CTF) |
| kipR | -1.83 | | -2.74 | -2.36 | -1.48 | transcriptional regulator (IclR family); |
| yqaF | -1.60 | | -2.12 | -1.59 | -0.28 | hypothetical protein |
| ylpC | -1.02 | | -0.89 | -0.57 | -2.65 | fatty acid biosynthesis transcriptional regulator |
| ybbH | -2.17 | | -3.30 | -1.84 | -1.77 | hypothetical protein |
| sigW | -0.12 | | -2.01 | -1.47 | -0.99 | RNA polymerase sigma factor SigW |
| yplP | -2.50 | | -2.64 | -1.86 | -0.56 | unknown; similar to transcriptional regulator (sigma-L-dependent) |
| fur | -0.48 | | 0.46 | 0.23 | -2.56 | transcriptional regulator (Fur family |
| tnrA | -2.20 | | -1.83 | -1.66 | -1.65 | transcriptional regulator |
| ysiA | -0.47 | | -1.69 | -1.50 | 1.46 | unknown; similar to transcriptional regulator (TetR/AcrR family) |
| ycnC | -0.07 | | 0.93 | 0.55 | -1.81 | unknown; similar to transcriptional regulator (TetR/AcrR family) |
| yvdT | -1.13 | | 0.15 | -0.71 | -2.19 | unknown; similar to transcriptional regulator (TetR/AcrR family) |
| ybfA | -1.17 | | -2.76 | -0.95 | -1.02 | hypothetical protein |
| nhaX | -0.83 | | -4.18 | -3.94 | 3.57 | putative regulatory gene for NhaC |
| ykoM | -0.61 | | -1.85 | -1.36 | 1.38 | unknown; similar to transcriptional regulator (MarR family) |
| ylbO | -0.12 | | -0.35 | -0.84 | -2.75 | hypothetical protein |
| ymfC | -0.35 | | 0.11 | 0.22 | -2.24 | unknown; similar to transcriptional regulator (GntR family) |
| yobD | -1.10 | | -1.97 | -1.11 | -0.39 | unknown; similar to transcriptional regulator (phage-related) (Xre family) |
| ytrA | -2.27 | | -1.27 | -1.41 | -3.65 | unknown; similar to transcriptional regulator (GntR family) |
| yttP | -0.97 | | -1.80 | -1.47 | -0.34 | hypothetical protein |
| pucR | -2.85 | | -2.91 | -3.11 | -2.26 | transcriptional regulator |
| yusO | -1.01 | | -0.54 | -0.49 | -1.68 | unknown; similar to transcriptional regulator (MarR family) |
| yvrI | -0.12 | | -0.43 | -1.50 | -1.65 | hypothetical protein |
| dnaA | 0.53 | | 1.69 | 1.28 | -0.67 | chromosomal replication initiation protein |
| rbsR | 0.84 | | 0.49 | 0.30 | 1.89 | transcriptional regulator (LacI family) |
| yxaF | 0.28 | | 1.63 | 1.56 | 0.09 | hypothetical protein |
| birA | 0.84 | | 1.70 | 1.22 | 0.17 | transcriptional regulator and biotin acetyl-CoA-carboxylase synthetase |
| iolR | 0.50 | | 1.83 | 0.71 | -0.51 | transcriptional regulator (DeoR family) |
| sigV | 0.78 | | 1.99 | -0.09 | -0.28 | RNA polymerase ECF(extracytoplasmic function)-type sigma factor (sigma-V); |
| yclA | 0.62 | | 0.03 | 0.20 | 2.49 | unknown; similar to transcriptional regulator (LysR family) |
| ydeT | 0.76 | | 1.90 | 0.67 | 0.55 | unknown; similar to transcriptional regulator (ArsR family) |
| ybgA | 0.99 | | 1.64 | 1.45 | 0.56 | unknown; similar to transcriptional regulator (GntR family) |
| yhdI | 0.00 | | 0.02 | -0.09 | 1.60 | unknown; similar to transcriptional regulator (GntR family) / aminotransferase (MocR-like) |
| sigM | 0.55 | | 1.68 | 0.80 | -0.81 | RNA polymerase sigma factor SigM |
| manR | 0.31 | | 1.06 | 0.70 | 2.02 | positive regulation of the mannose operon (manPA-yjdF) |
| yonR | 0.66 | | 2.04 | 1.04 | -0.41 | unknown; similar to transcriptional regulator (phage-related) (Xre family) |
| yraB | 0.27 | | 1.49 | 0.89 | 1.76 | unknown; similar to transcriptional regulator (MerR family) |
| ytlI | -0.17 | | 2.26 | 0.49 | 0.13 | unknown; similar to transcriptional regulator (LysR family) |
| mecA | -0.28 | | -0.46 | -0.30 | 1.71 | negative regulator of competence |

In *B. subtilis*, the *pai* operon, encoding *paiA* and *paiB*, is involved in negative control of sporulation as well as the production of extracellular and cell-associated proteases and other enzymes such as α-amylase and alkaline phosphatase. The overexpression of the two genes down-regulates the amount of transcripts for the extracellular metalloprotease, suggesting that this operon negatively controls the protease levels through transcriptional repression [1]. However, Farhad Forouhar *et al.* demonstrated that PaiA is a novel SSAT (*N*-acetyltransferase capable of acetylating both spermidine and spermine), not a transcriptional factor. The *pai* operon may be involved in polyamine homeostasis. Polyamines are small, aliphatic cations that have important roles in many biological processes, including DNA binding and stability, chromatin condensation, RNA binding and conformation, mRNA translation, protein binding, and other processes. The intracellular levels of these cations are tightly regulated, through biosynthesis, import, degradation, and export pathways. SSAT catalyzes the first reaction in both the degradation and the export pathways for polyamines [2]. *pai* operon was significantly downregulated at 5-30 min, 30-120 min (about 15-fold lower), and at 120 min after treatment with Val, Gln, and Glu, respectively (Fig.S7A). The other degradative enzyme genes (*aprE, nprE, phoA, amyE*) did exhibited a clear induction, whearas *aprE* and *nprE* were repressed by Glu (Fig.S7B). The amino acid availability may accelerate biosynthesis of polyamines, and suppress degradation and the export pathways for polyamines. The expression of *ctsR, nhaX*, and *sigB*, as stress regulatory proteins, was shown in Fig.S7C. CtsR (class three stress gene repressor) negatively regulates the expression of class III heat shock genes (*clpP, clpE* and the *clpC* operon). The expression of *ytrA, yvyD, yplP,* and *ybfA* were shown in Fig.S8. *yplP, ybfA* were probable unknown transcriptional regulators. ytrA negatively regulates ytrABCDEF operon, encoding a putative ATP-binding cassette (ABC) transport system involved in acetoin utilization of *B. subtilis*. It is known that *B. subtilis* produces acetoin as an external carbon storage compound using carbon-overflow pathways and then reuses it later during stationary phase and sporulation. The genes encoding the enzymes for acetoin production have been reported to form a single operon, *alsSD*. In addition, it has been proposed that there are at least two systems for acetoin catabolism: one encoded by the *acuABC* (ccpA negatively) genes and the other by the *acoABCL* genes (ccpA sigL)[3]. CcpA activates the expression of the genes *alsSD* that are required for the synthesis of acetoin, and CodY contributes to the activation of the acetate- and lactate-synthesis pathways. Additionally, the re-utilization enzymes for acetate and acetoin are repressed by both CcpA and CodY. It was reported that the strong induction of YvyD was caused by amino acid starvation [4]. Our transcriptional data appeared to show that *yvyD* and *ytrA* displayed reverse correlation of expression in response to amino acid addition (Fig.S8D). An intriguing explanation for this result could be that YvyD may be somehow involved in the re-utilization of by-products of carbon-overflow pathways, such as lactate, acetate and acetoin.


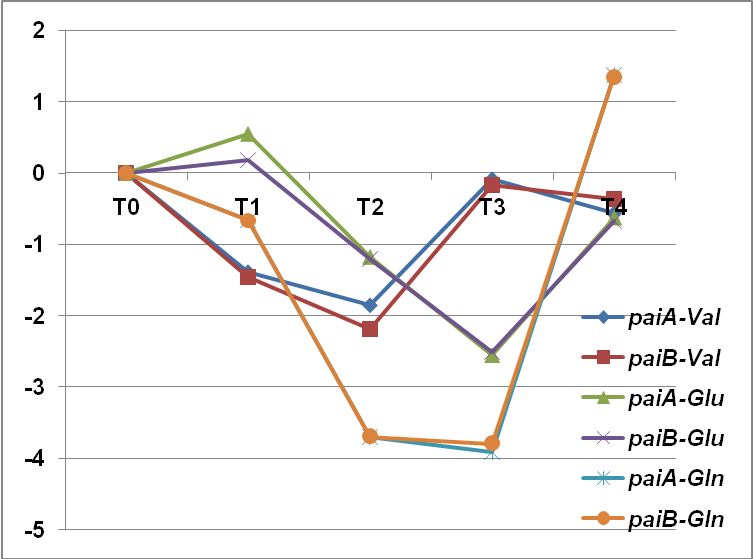

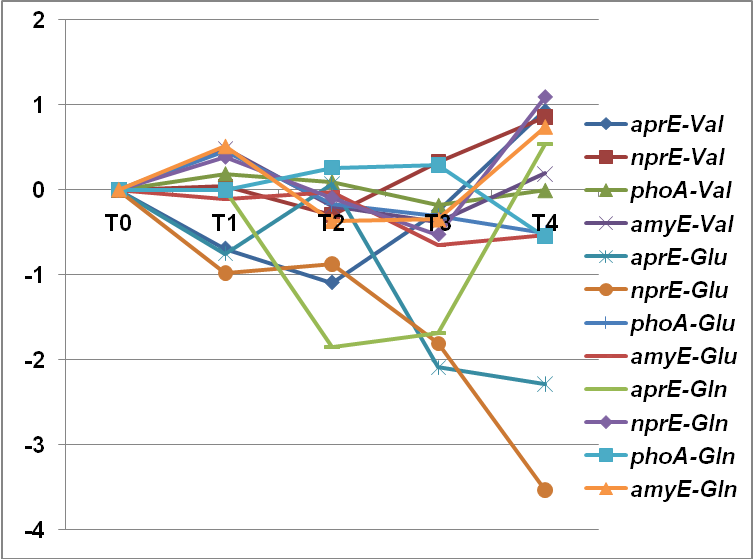

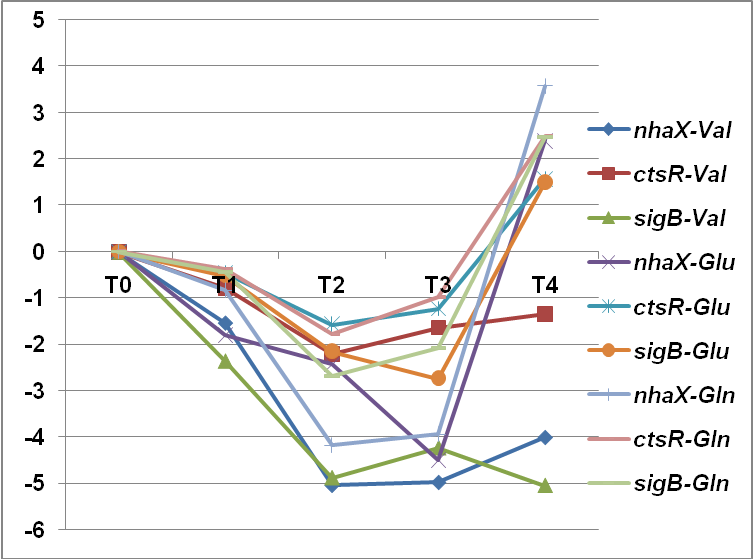


Figure S7. Expression pattern of the genes related to paiA/B (A), degradative enzyme genes (B), and stress regulatory proteins (C).


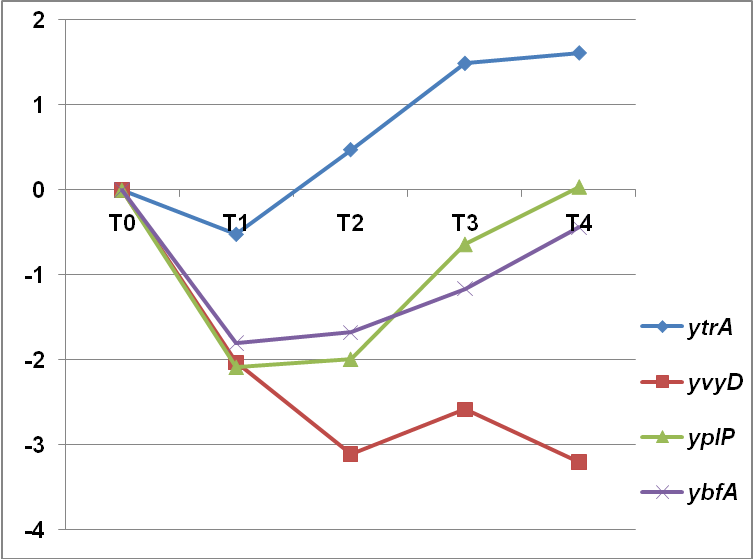

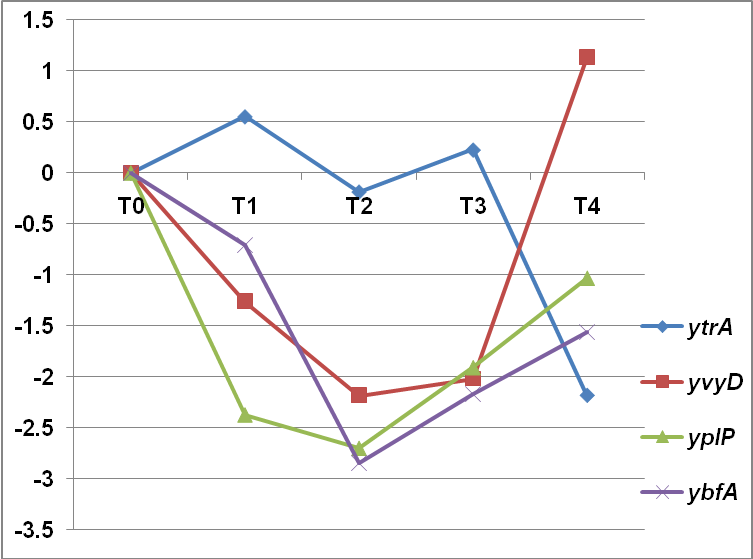

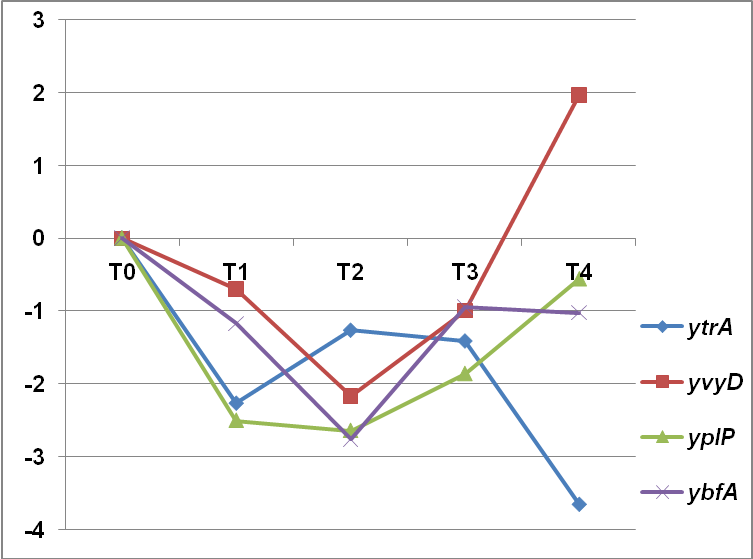

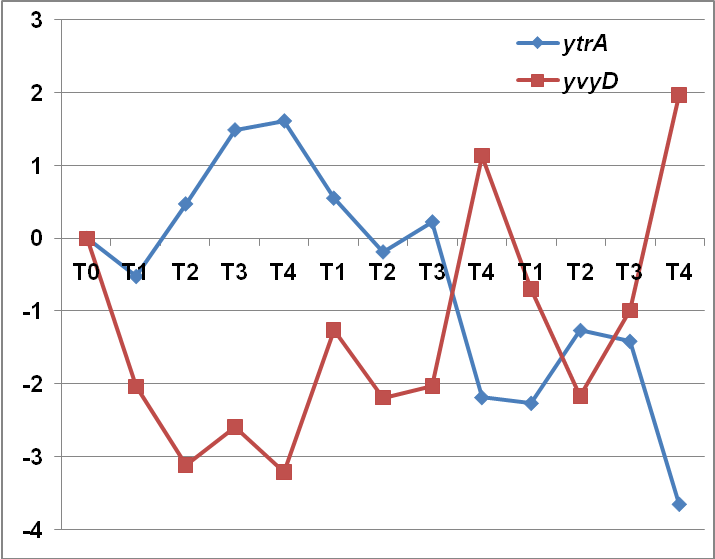


Figure S8. Expression pattern of ytrA, yvyD, yplP, and ybfA after treatments with Val (A), Glu (B), and Gln (C). (D) *yvyD* and *ytrA* displayed reverse correlation of expression in response to amino acid addition.

Seven TF genes were overrepresented by both Val and Glu, including *yhgD, cheB, tenA, tenI, spxA, sigI,* and *sigD*. *sigD* and *cheB* genes, involved in flagellar synthesis, motility, and chemotaxis, exhibited similar expression pattern (Fig. S9B). TenA and TenI have been implicated in regulating the production of extracellular proteases. As *paiA* and *paiB*, *tenAI* genes were also significantly repressed by Val and Glu (about 73-fold lower at 120 min) (Fig. S9A). Thiamin pyrophosphate is an essential cofactor in all living systems and is biosynthesized by a complex pathway. TenA, as thiaminase II, is involved in the salvage of the thiamin pyrimidine from base-degraded thiamin [5]. TenI shows significant structural homology to thiamin phosphate synthase, and exerts an opposite effect to reduce alkaline protease production. YhgD is a putative uncharacterized HTH-type transcriptional regulator of TetR/AcrR family. Spx is a global transcriptional regulator of the oxidative stress response in *B. subtilis*. The induction of *spxA* (*yjbD*) gene is associated with a variety of stress conditions including heat, salt, disulfide, and peroxide stress. SpxA governs the expression of genes functioning in sulfur metabolism. SpxA promotes negative control (by stimulating YrzC production) of operons that function in cysteine synthesis from alternative sulfur sources but stimulates *yrrT* operon expression in the absence of cysteine, thus promoting cysteine synthesis through S-adenosyl methionine catabolism [6]. *sigI* gene, which is a member of the class *VI* heat shock genes of the *B. subtilis* heat shock stimulon, encodes an alternative sigma factor of the σ^70^ family whose regulon is poorly defined. Recent study reported that overexpression of the *B. subtilis sigI* gene could specifically stimulate expression of both the actin homolog gene *mreBH* and the bacitracin resistance gene *bcrC (ywoA)* of *B. subtilis*. The *B. subtilis bcrC* gene encodes an undecaprenyl pyrophosphate phosphatase that is important for cell wall biosynthesis and for bacitracin resistance. *mreBH* gene encodes a bacterial homolog of actin that is important for cell morphogenesis. The critical role of SigI in regulation of *bcrC* and *mreBH* implies that one function of SigI is related to the maintenance of cell envelope integrity and homeostasis during heat stress [7]. In this study, *sigI* gene was induced by Val and Glu. As described, the yvrH-regulon, appears to be related to cell membrane and cell wall function, also was induced by Val and Glu. These data demonstrated that valine and glutamate activated some genes involved in cell membrane and cell wall function.

Four TF genes were overrepresented by both Val and Gln, including *dnaA, birA, ylpC*, and *yvdT*. As shown in Fig.S9, *dnaA* and *birA* genes were induced by three amino acids (about 2.6-fold higher by Glu). DnaA plays an important role in the initiation and regulation of chromosomal replication. Biotin (vitamin H) is an essential cofactor for a class of important metabolic enzymes, biotin carboxylases and decarboxylases. Bifunctional protein BirA acts both as a biotin-operon repressor and as the enzyme that synthesizes the corepressor, acetyl-CoA: carbon-dioxide ligase [8].

Four TF genes were overrepresented by both Val and Gln, including *dnaA, birA, ylpC*, and *yvdT*. As shown in Fig.S9, *dnaA* and *birA* genes were induced by three amino acids (about 2.6-fold higher by Glu).


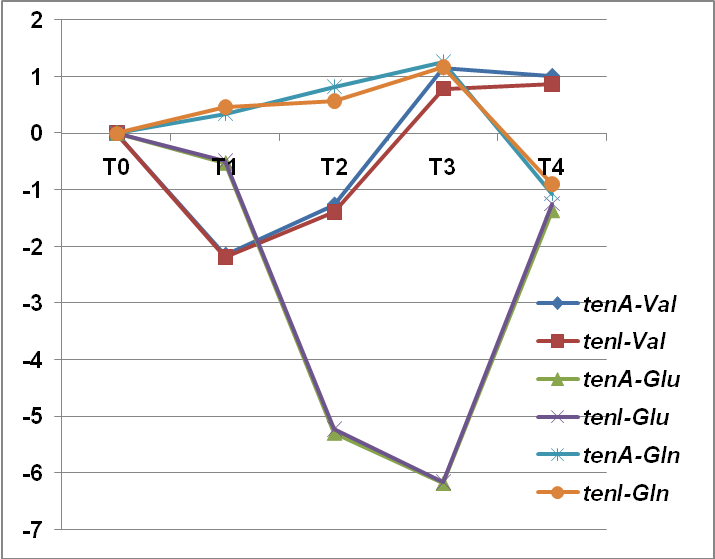

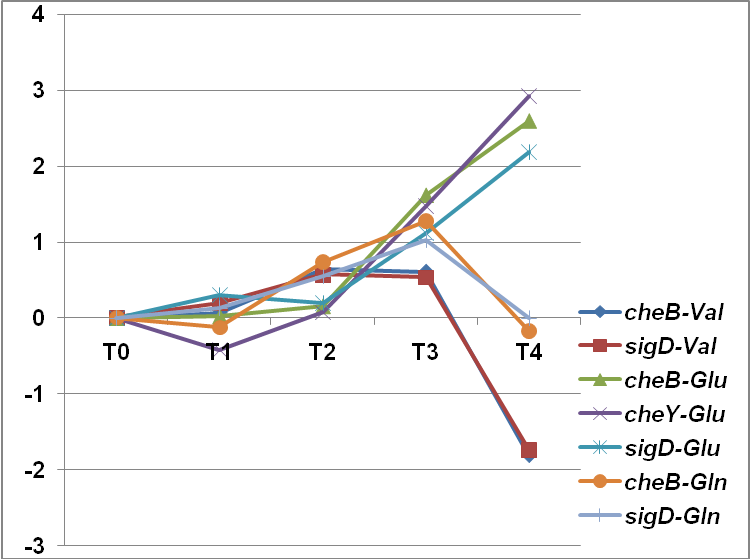

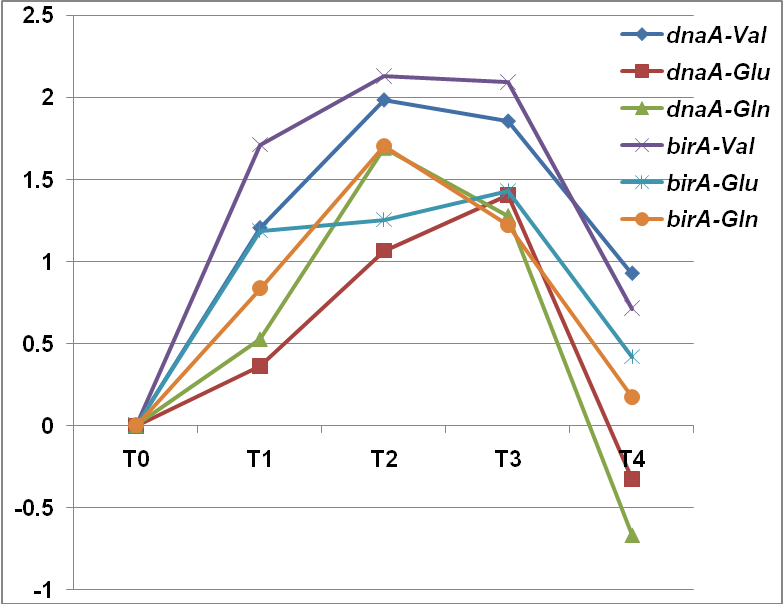

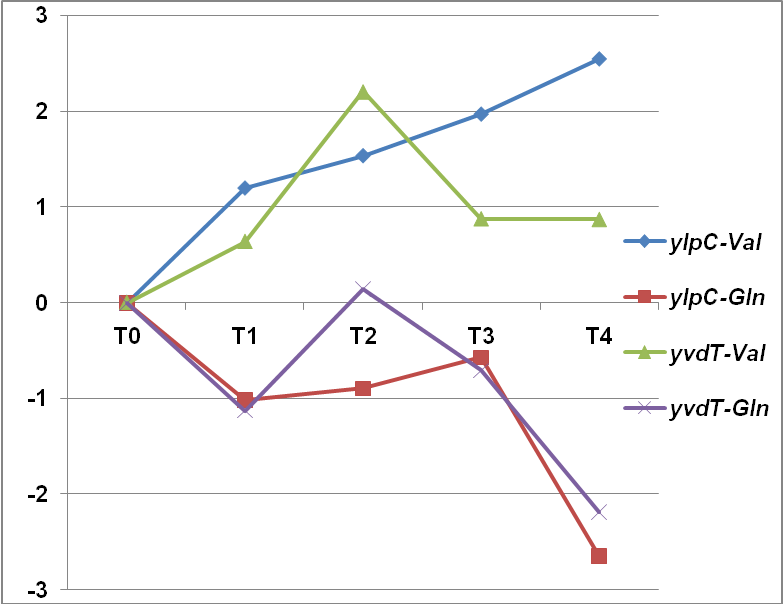


Figure S9. Expression pattern of the genes related to tenA/I (A), sigD and cheB (B), *dnaA* and *birA* genes (C), *ylpC* and *yvdT* (D) in response to amino acid addition.

Eleven TF genes were overrepresented by both Glu and Gln, including *hpr, gltC, fur, ysiA, ykoM, ymfC, yvrI, kipR, ycnC, ytlI,* and *sigM*. Most of these TF genes were downregulated by Glu and Gln. YtlI was involved in cysteine metabolism and sulfur metabolism. Hpr negatively regulate sporulation and extracellular proteases genes (*aprE, nprE, sin*). TnrA, GltC, and GlnR are the important transcriptional regulators, regulating nitrogen metabolism. It is not surprising that glutamate and glutamine exhibited significant effects on the expression of genes involved in nitrogen metabolism. Indeed, as shown in Fig.S10, genes of *gltA, gltC,* and *gltC* were significantly repressed by glutamate (31-, 6-, 52-fold). The metabolite 2-oxoglutarate stands at the crossroads between carbon metabolism and nitrogen metabolism. 2-oxoglutarate provides the carbon skeleton for the two most important nitrogen-containing compounds in the cell, glutamate and glutamine using glutamate synthase (GltA). Moreover, 2-oxoglutarate also provides entry point into central metabolism for the carbon skeletons of some amino acids (including arginine, ornithine, proline and histidine) through glutamate metabolism by glutamate dehydrogenase (RocG). Availability of glutamate inactivated carbon flux from TCA to amino acid by repressing *gltA, gltC, gltC,* and *yerD* (glutamate synthase). However, the activation of the synthesis of 2-oxoglutarate from glutamate by RocG was not observed. NAD synthetase (NadE) and ArgJ from glutamine and glutamatewere stimulated. Genes of *gltA, gltC, gltD, glnA, glnM, glnR, yerD,* and *tnrA* were significantly repressed by glutamine (30-, 10-, 40-, 9-, 5-, 5-, 5-fold). These results demonstrated that the metabolite flux from 2-oxoglutarate to glutamate to glutamine was obviously reduced. Meanwhile, the fluxes from glutamine to glutamate (*yccC* induced by 26-fold), to GMP (*guaA* 3.5-fold), to NAD^+^ (*nadE* 10-fold), and to purine metabolism (*purD* 4-fold) were increased.


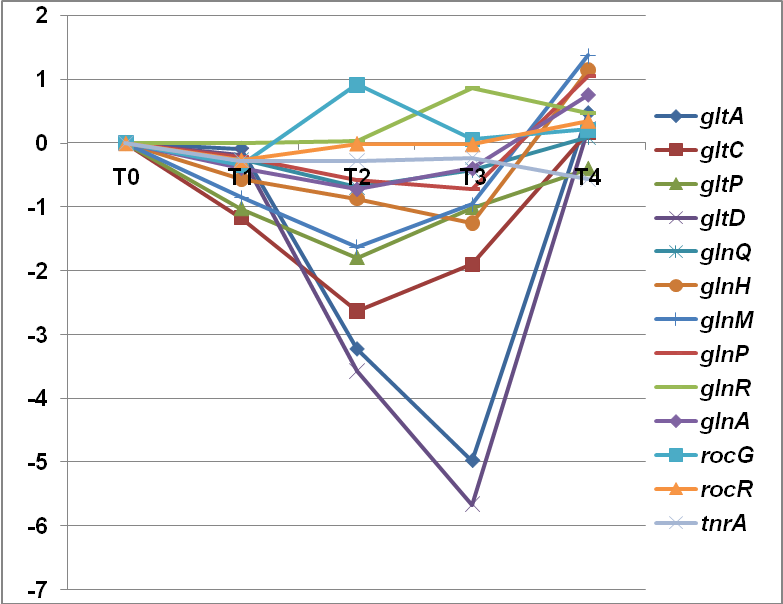

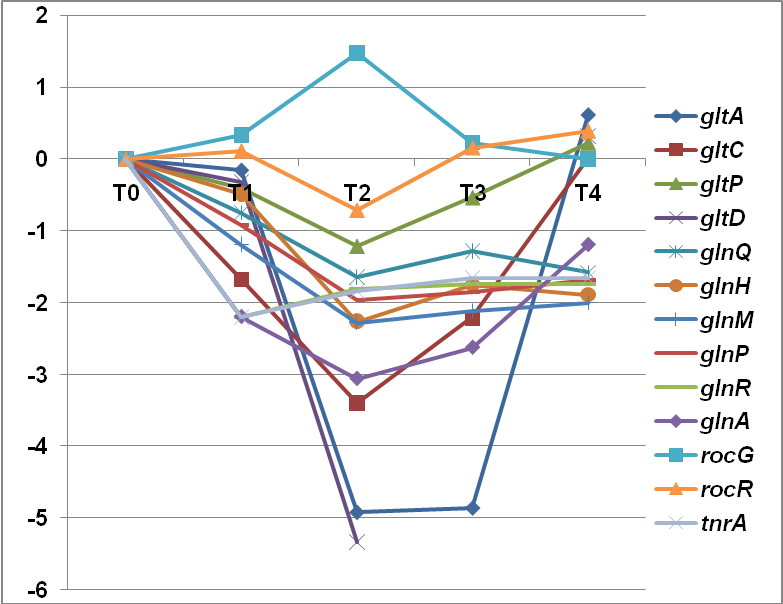


Figure S10. Expression pattern of the genes related to nitrogen metabolism in response to Glu (A), and Gln (B).

The YsiA (now renamed by FadR) regulon, involved to the fatty acid β-oxidation pathway. Fatty acid metabolism in *B. subtilis* is likely controlled by YlpC (FapR) (controlling fatty acid biosynthesis and phospholipids) and YsiA (controlling fatty acid degradation). YlpC presumably senses the in vivo concentration of malonyl-CoA, an increase of which inactivates this protein, whereas YsiA is most likely inactivated by an increase in the in vivo concentration of long chain acyl-CoAs [9,10]. YlpC (FapR) negatively regulates the expression of many genes involved in fatty acid and phospholipid metabolism (the fap regulon). Control of FapR by the cellular pools of malonyl-CoA provides a mechanism for sensing the status of fatty acid biosynthesis and to adjust the expression of the fap regulon accordingly. Valine induced *ylpC* and *yvdT*, whereas glutamine repressed *ylpC* and *yvdT*. The function of YvdT is still unclear.

Beside these TF genes, isoleucine and valine utilization (*bkd* operon) regulator *bkdR*, lincomycin operon (*lmrBA*) negative regulator *lmrA*, probable transcriptional regulators *yvdT, ypuH, yrxA, ycnK,* and *yvhJ* were induced by valine. In *B. subtilis*, at least four genes encoding multidrug resistance efflux transporters have been characterized, namely, *blt*, *bmr*, *lmrB*, and *bmr3 (mdr)*. The expression of all genes related to multidrug resistance was shown in Fig.S11. Although *lmrA* displayed some extent induction, *lmrB* was not repressed. Whereas, *bmrU* gene, in response to cellular invasion by certain lipophilic cationic compounds (drugs), was significantly downregulated by three amino acids.


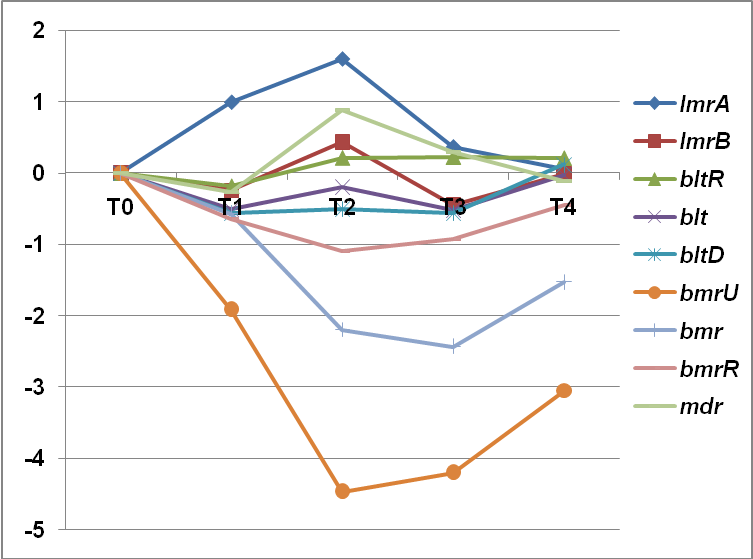

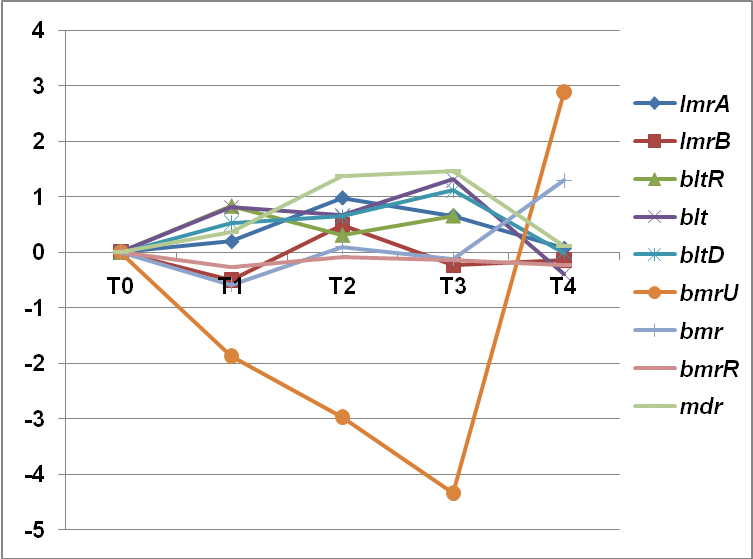

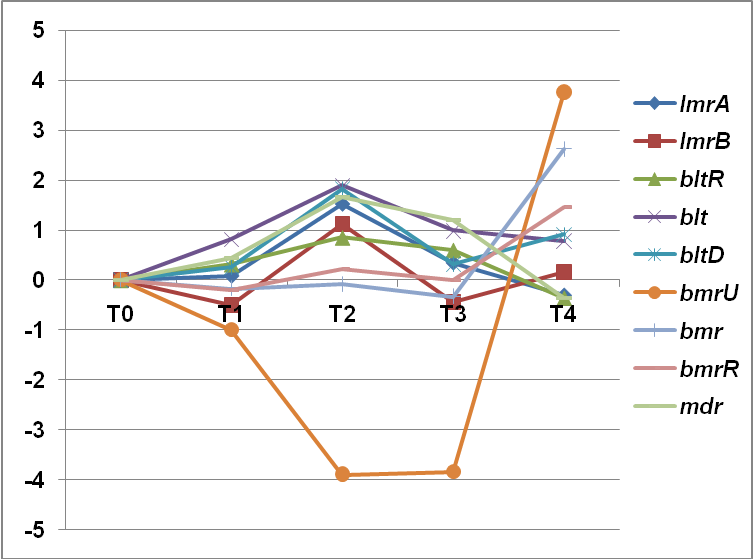


Figure S11. Expression pattern of all genes related to multidrug resistance after treatments with Val (A), Glu (B), and Gln (C).

cggR and the central glycolytic genes were obviousely repressed by valine. The central glycolytic genes repressor (CggR) controls the transcription of the *cggR-gapA-pgk-tpiA-pgm-eno* operon encoding encoding five key glycolytic enzymes that catalyse the five steps of the central carbon metabolism, *tpi* (triose phosphate isomerase), *gapA* (glyceraldehyde-3-phosphate dehydrogenase), *pgk* (phosphoglycerate kinase), *pgm* (phosphoglycerate mutase) and *eno* (enolase). The second gene, which putatively encodes a glyceraldehyde-3-phosphate dehydrogenase (GAPDH), *gapB*, very similar in sequence but is in the *gapB-speD* operon. It is reported that *gapA* and *gapB* are submitted to opposite regulation: *gapA* is transcribed at a basal level under gluconeogenic conditions of growth, and its transcription is enhanced in the presence of glucose or other glycolytic carbon sources; in contrast, *gapB* transcription is repressed in the presence of glycolytic carbon sources and strongly induced under gluconeogenic conditions of growth [11]. Stéphane Aymerich *et al.* found that fructose-1,6-biphosphate (FBP) acts as an inhibitor of CggR DNA-binding activity [12]. Ludwig *et al.* suggested that CggR could respond synergistically to two different signals, a catabolic one derived from the presence of sugars, and an anabolic one derived from amino acid metabolism, and the presence of sugars and amino acids synergistically results in the induction of the *gapA* operon [13]. In our study, *gapA* operon was significantly repressed by treatment with valine at 30 min (8-fold), was slightly repressed by glutamine at 30 min (2.5-fold), was not impacted by glutamate. Meanwhile, *gapB* transcription was obviously induced only by glutamate (34-fold) and glutamine (5-fold) (Fig. S12).


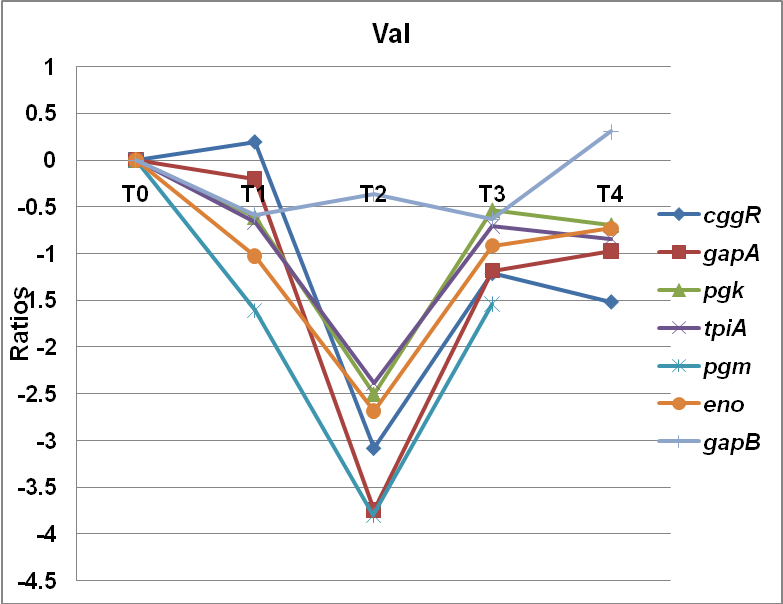

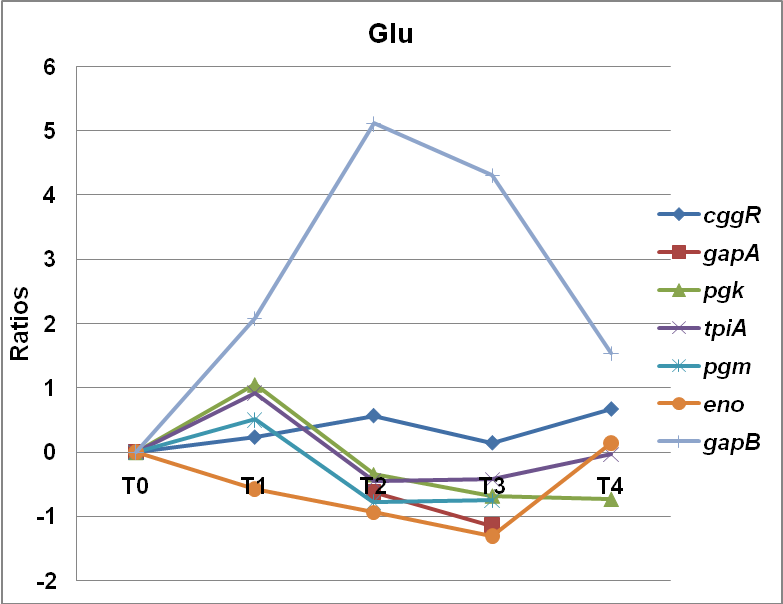

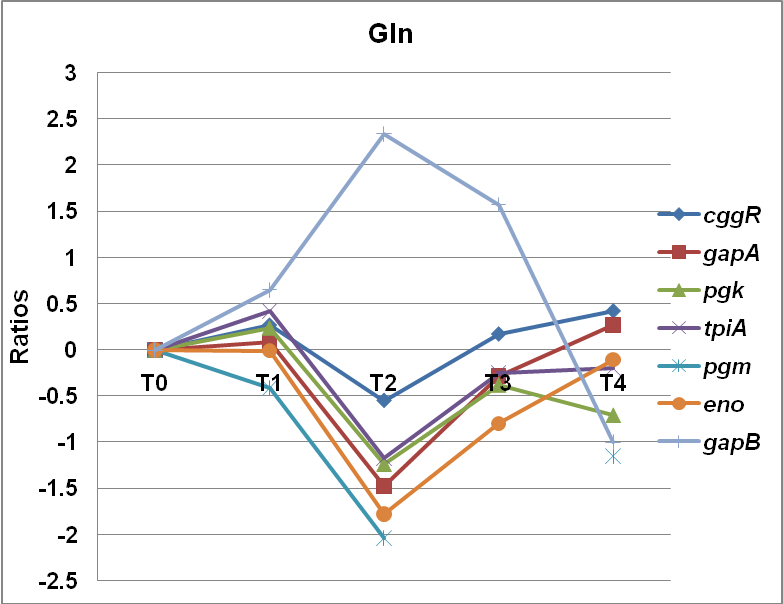


Figure S12. Expression pattern of the central glycolytic genes after treatments with Val (A), Glu (B), and Gln (C).

YdgG and YhbI were also two of TFs that were the most significantly repressed (69-fold and 40-fold) by glutamate. It was reported *ydgG* contained LexA binding sites within its promoter region, but was not significantly induced by MC or UV in wild-type cells. *ydgG* maybe not an additional SOS genes in *B*. *subtilis* [14]. So far, the exact roles of YdgG and YhbI in conferring transcriptional regulation remain mysterious, as two of transcriptional regulators of MarR family.

Most of TF genes were downregulated by glutamate, only 12 genes were upregulated, including *cheB, sigD, sigI, ycnC, slr, cheY, cheV, licT, azlB, kipR, ytlI,* and *pyrR*. PyrR is involved in repression of the *pyr* operon (pyrRPBCAAABKDFE) for pyrimidine biosynthesis.

Some function-unassigned transcriptional regulator genes *ybbH, yqaF, yobD, ybfA,* and *yttP* were reduced quickly to Gln addition, with *ybbH* being the most responsive (repression with 10-fold).

Genomic sequencing of various microorganisms has revealed the presence of many two-component regulatory systems in every species. In *Bacillus subtilis*, 36 sensor kinases and 35 response regulators have been found, among which each of 30 kinase-regulator pairs resides in an operon on the genome. The addition of amino acids displayed slight effect on the expression of these genes. Indeed, we observed repression of chemotaxis TCS (*cheA, cheB, cheW*) and *citST*, and *ybdKJ* and *yocF* were clearly induced, whereas *resE* and *cheY* also exhibited a moderate induction by valine, but did not fulfil the threefold threshold. The *citST* was involoved in transport of divalent metal ions/citrate complexes (*citM*), and was repressed in the presence of glucose by the general transcription factor CcpA. The signals regulating the kinases *ybdK* and *yocF* are still unknown. It was reported that *ybdK* of the putative *ybdJK* two-component system was regulated by ComK, which suggests that the YbdJ response regulator might affect competence-specific gene transcription. The glutamate clearly induced chemotaxis TCS (*cheA, cheB, cheY, cheV,* and *cheW*) gene and qurum-sensing sytem *comX* gene (Fig. S13). None of the genes encoding TCS systems induced or repressed after treatment with Gln was found using threefold criteria.


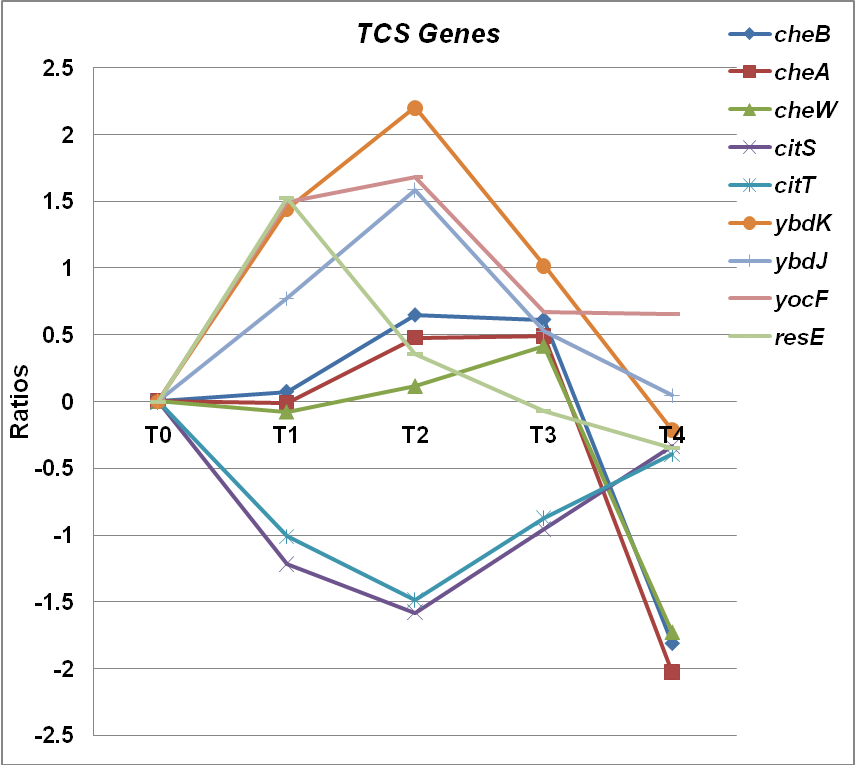

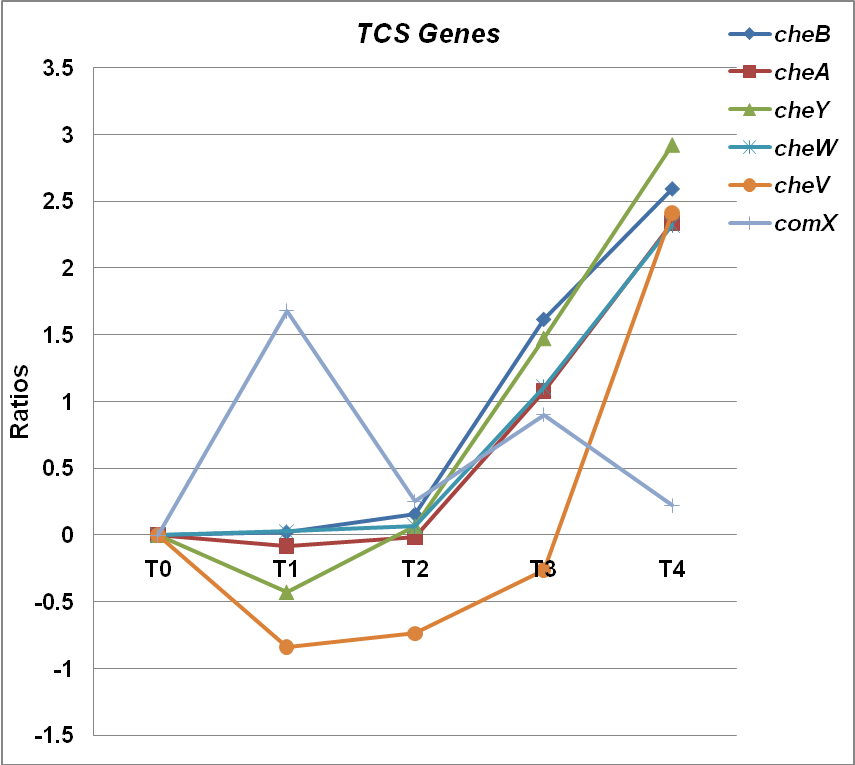


Figure S13. The expression of TCS genes with threefold changes after treatment of valine (A) and glutamate (B).

Reference

1. Honjo M, Nakayama A, Fukazawa K, Kawamura K, Ando K, et al. (1990) A novel *Bacillus subtilis* gene involved in negative control of sporulation and degradative-enzyme production. J Bacteriol 172:1783-1790.
2. Forouhar F, Lee IS, Vujcic J, Vujcic S, Shen J, et al. (2005) Structural and Functional Evidence for *Bacillus subtilis* PaiA as a Novel *N*^1^-Spermidine/Spermine Acetyltransferase. J Biol Chem 280:40328-40336.
3. Yoshida KI, Fujita Y, Ehrlich SD (2000) An Operon for a Putative ATP-Binding Cassette Transport System Involved in Acetoin Utilization of *Bacillus subtilis.* J Bacteriol 182: 5454-5461.
4. Kathrin D, Christine E, Gerhard M, Michael H (1998) The *yvyD* Gene of *Bacillus subtilis* is under Dual Control of ^B^ and ^H^. Journal of Bacteriology 180:6674-6680.
5. Jenkins AL, Zhang Y, Ealick SE, Begley TP (2008) Mutagenesis studies on TenA: a thiamin salvage enzyme from *Bacillus subtilis*. Bioorg Chem 36:29-32.
6. Choi SY, Reyes D, Leelakriangsak M, Zuber P (2006) The Global Regulator Spx Functions in the Control of Organosulfur Metabolism in *Bacillus subtilis**.* Journal of Bacteriology 188: 5741-5751.
7. Tseng CL, Shaw GC (2008) Genetic Evidence for the Actin Homolog Gene mreBH and the Bacitracin Resistance Gene bcrC as Targets of the Alternative Sigma Factor SigI of Bacillus subtilis. J Bacteriol 190:1561-1567.
8. Perkins JB, Bower S, Howitt CL, Yocum RR, Pero J (1996) Identification and characterization of transcripts from the biotin biosynthetic operon of *Bacillus subtilis.* J Bacteriol 178: 6361-6365.
9. Hiroshi M, Kazutake H, Yasutaro F (2007) Organization and Function of the YsiA Regulon of *Bacillus subtilis* Involved in Fatty Acid Degradation. J Biol Chem 282:5180-5194.
10. Chiara B, Alessandro G, Giorgio M, Mila R, Elena T, et al. (2007) *Bacillus subtilis* Gene Cluster Involved in Calcium Carbonate Biomineralization. J Bacteriol 189:228-235.
11. Fillinger S, Boschi-Muller S, Azza S, Dervyn E, Branlant G., eta l. (2000) Two glyceraldehyde-3-phosphate dehydrogenases with opposite physiological roles in a nonphotosynthetic bacterium. J Biol Chem 275:14031-14037.
12. Doan T, Aymerich S (2003) Regulation of the central glycolytic genes in Bacillus subtilis: Binding of the repressor CggR to its single DNA target sequence is modulated by fructose-1,6-bisphosphate. Mol Microbiol 47:1709-1721.
13. Ludwig H, Homuth G, Schmalisch M, Dyka FM, Hecker M, et al. (2001) Transcription of glycolytic genes and operons in Bacillus subtilis: evidence for the presence of multiple levels of control of the gapA operon. Mol Microbiol 41:409-422.
14. Nora A, Elke KS, Veena M, Laura EB, Susan PC, et al. (2005) Genetic Composition of the *Bacillus subtilis* SOS System. J Bacteriol 187:7655-7666.
